# Delay-Modulated Nonlinear Stochastic Mode Veering in Inertially Coupled Vibration Systems

**DOI:** 10.3390/e28090952

**Published:** 2026-08-24

**Authors:** Lili Zhang, Zikun Han, Qiubao Wang

**Affiliations:** 1 Department of Mathematics and Physics, Shijiazhuang Tiedao University, Shijiazhuang 050043, China; zllyou@126.com; 2 Department of Engineering Mechanics, Shijiazhuang Tiedao University, Shijiazhuang 050043, China; hanzikun@stdu.edu.cn

**Keywords:** mode veering, inertial coupling, vibration systems, delay differential equation, nonlinear backbone, stochastic spectral cloud

## Abstract

Mode veering is a modal-interaction phenomenon found in vibration systems. For inertially coupled structures, the combined influence of coupling delay, nonlinear restoring force, and stochastic coupling perturbation remain insufficiently understood. This work analyzes an inertially coupled two-coordinate prototype in which a discrete delay, a delayed cubic stiffness, and positive multiplicative stochastic modulation all enter through the same relative-coordinate coupling channel. We formulate the delayed linear spectrum through a quasi-polynomial characteristic equation. We also characterize the veering by the two positive-frequency characteristic-root branches descending from the mechanical modes. Coupling delay shifts the veering center, alters the minimum frequency gap, and moves the tracked rightmost roots toward the stability boundary. An analytical imaginary-axis-crossing criterion is derived to determine the delay-induced stability boundary of the deterministic linearized system, and the resulting boundary is independently validated by direct multi-start characteristic-root searches and Chebyshev-collocation approximation of the DDE generator. A fixed-reference modal-coordinate representation identifies the off-diagonal modal terms associated with branch exchange while retaining the full delayed characteristic equation. A first-harmonic treatment of the delayed cubic term can yield an amplitude-dependent nonlinear veering backbone. For the stochastic problem, frozen lognormal coupling samples and a time-dependent Ornstein–Uhlenbeck-driven multiplier are constructed from the same unit-mean positive lognormal marginal law. The former is used to quantify realization-wise spectral broadening, whereas the latter retains temporal correlation and is used to evaluate finite-time branch residence and pathwise delayed-work statistics. The pathwise energy balance reveals that the delayed relative-coordinate work rate is sign-indefinite. This provides a common energy-transfer mechanism through which delay, nonlinearity, and stochastic modulation reshape mode veering in the inertially coupled system.

## 1. Introduction

Mode veering is a modal-interaction phenomenon in which neighboring vibration branches repel each other, exchange modal character, and may localize vibratory energy as a structural or coupling parameter is varied. This phenomenon has long been observed in disordered or weakly coupled structures and continues to be relevant for interpreting closely spaced modes in practical vibration measurements [1,2]. Recent studies further demonstrate the practical importance of closely spaced and interacting vibration modes. Xie et al. [3] developed a two-stage modal-identification procedure specifically for structures with closely spaced modes, highlighting the difficulty of reliably separating neighboring modal components in measured responses. Laine et al. [4] investigated electromagnetic influences on torsional vibration in the frequency domain, while Akay et al. [5] experimentally examined smoothly nonlinear rotordynamic behavior. These studies indicate that modal interaction in practical vibration systems is often accompanied by additional electromechanical or nonlinear effects. Related developments have also appeared in tunable and coupled vibration systems. Rezaei et al. [6] investigated self-powered tunable mechanical oscillators, Farsadi et al. [7] considered frequency optimization of variable-stiffness composite panels in the presence of geometric nonlinearity, and Wang et al. [8] developed an electromechanically coupled dynamic model for a servo press. Collectively, these studies motivate the need to examine modal interactions in systems where stiffness variation, nonlinear response, and coupled energy-transfer paths coexist. Yet most classical veering diagrams are constructed from the real eigenfrequencies of instantaneous linear systems. Such diagrams do not directly explain what occurs when the coupling path is delayed, nonlinear, and randomly modulated.

Inertial coupling provides a concrete mechanical setting for this question. Inertial amplification and relative-motion mechanisms have been used to create low-frequency band gaps, enhance vibration attenuation, and design lightweight vibration absorbers [9,10,11]. More recent investigations have extended these concepts toward nonlinear absorbers and nonlinear metastructures. Yao et al. [12] proposed an inertia-amplified nonlinear absorber for robust pulse mitigation in lightweight structures. Bai et al. [13] reviewed recent developments in nonlinear vibration metamaterials, emphasizing the role of nonlinear mechanisms in tailoring vibration transmission, while Fang et al. [14] summarized advances in nonlinear acoustic and elastic metamaterials and metastructures. These developments show that inertial amplification and nonlinear coupling are increasingly being combined to manipulate vibration and wave-transfer characteristics. Most directly, Jacques and Adhikari demonstrated that pure inertial coupling, or combined stiffness–inertial coupling, can itself induce mode veering in a two-coordinate oscillator [15]. Thus, inertial coupling is not merely a mass-redistribution device. It can reorganize modal interaction and should be examined under more realistic coupling conditions.

One such condition is delay. Recent studies have demonstrated several ways in which deliberately introduced delay can be incorporated into vibration-absorption systems. Peichl et al. [16] considered the integrated design of structural parameters and a delayed resonator for efficient non-collocated vibration absorption. Liu and Cheng [17] developed a high-static–low-dynamic-stiffness delayed-resonator vibration absorber, whereas Liu et al. [18] investigated the $H_{\infty }$ optimization of a hybrid multiple-delayed-resonator absorber. These works demonstrate the potential of delay as an active design ingredient for vibration suppression, but their primary focus is vibration-absorber performance rather than the delay-induced evolution of complex characteristic-root veering. Mathematically, delayed force transmission leads to delay differential equations whose stability is governed by infinitely many characteristic roots. For this reason, a delay-sensitive veering analysis cannot rely solely on undamped real eigenfrequency branches. Rightmost-root computation and pseudospectral approximation are necessary to determine whether the veering region also lies close to a stability boundary [19,20].

A second condition is nonlinearity. Cubic restoring forces are standard local models for amplitude-dependent stiffness in nonlinear vibration. Nonlinear normal-mode or energy-transfer viewpoints are used to interpret modal interaction away from the infinitesimal-amplitude limit [21]. Recent work illustrates that nonlinear and stochastic effects can alter vibration-transfer mechanisms in different but complementary ways. Bai et al. [13] reviewed nonlinear vibration metamaterials, and Yao et al. [12] demonstrated an inertia-amplified nonlinear absorber in which nonlinear response directly participates in vibration mitigation. From a stochastic perspective, Breunung and Balachandran [22] investigated nonlinear vibration attenuation through stochastic response modification. Uncertainty has also been incorporated into more detailed structural models. Pandey et al. [23] addressed modeling and stochastic updating of nonlinear structural joints, Abdolahi and Malekzadeh [24] considered stochastic optimization and dynamic analysis under unpredictable initial conditions, and Chatterjee et al. [25] investigated stochastic dispersion and optimal design in locally resonant metamaterial nanobeams. These studies establish the importance of uncertainty in nonlinear vibration systems, but they do not address how stochastic modulation interacts with delay and nonlinear coupling to reshape the characteristic-root branches associated with mode veering. The probabilistic interpretation and time-domain simulation of such responses rely on random-vibration and stochastic-numerics tools [26]. Entropy-based time-series measures offer a complementary approach to distinguish deterministic structure from noise-driven variability in nonlinear data [27,28,29]. We do not adopt them as observables here, as the present question concerns delay spectra and branch-energy residence rather than scalar complexity classification. Thus, for an inertially coupled veering system, it is natural to ask how a delayed nonlinear relative-coordinate channel behaves when its effective coupling strength fluctuates.

Despite these developments, the combined mechanism remains insufficiently clarified. Existing inertial-coupling veering theory accounts for the deterministic instantaneous limit [15]. Delayed-resonator studies explain how time delay can be used for vibration absorption, but they do not focus on complex-root veering in an inertia-coupled oscillator [16,17]. Recent stochastic and nonlinear vibration studies provide tools for uncertainty, nonlinear attenuation, and stochastic updating, but they do not identify a pathwise energy-transfer mechanism for stochastic nonlinear branch exchange near veering [22,23,24]. Therefore, the unresolved issue is not whether delay, nonlinearity, or randomness is important individually, but how they jointly reshape modal veering when all three act through the same relative-coordinate coupling channel of an inertially coupled system.

We address this issue with a mechanism-oriented analysis of an inertially coupled vibration system with delayed relative-coordinate feedback. The model contains the deterministic inertial-coupling veering oscillator as a limiting case, but extends it by placing a discrete delay, a delayed cubic stiffness, and positive stochastic modulation in the same relative-coordinate coupling path. The scope is deliberately finite-dimensional and mechanism-oriented. The analysis follows a traceable chain from complex characteristic roots to modal branch-energy exchange, while keeping the Monte Carlo evidence within its finite-time statistical meaning. The main contributions are as follows:

1. A Lagrange–d’Alembert derivation of an inertially coupled vibration model in which linear delay, cubic delay, and positive stochastic modulation act through the same relative-coordinate channel.
2. A complex-root veering analysis showing how coupling delay shifts the veering center, changes the minimum frequency gap, and moves the rightmost characteristic roots toward instability.
3. An analytical imaginary-axis-crossing criterion for the delay-induced stability boundary, together with independent validation and numerical-convergence assessment using direct quasi-polynomial root searches and Chebyshev-collocation approximation.
4. A fixed-reference modal-coordinate representation that identifies the off-diagonal delayed, damped, and detuned coupling terms entering the branch-interaction and branch-energy diagnostics, without assuming diagonalization of the full DDE operator.
5. A first-harmonic nonlinear backbone showing how delayed cubic coupling produces amplitude-dependent veering deformation.
6. A frozen stochastic spectral-cloud analysis and time-domain Ornstein–Uhlenbeck Monte Carlo validation showing residence-based branch-energy exchange, together with robustness checks for time step, sample size, and threshold choices.

The remainder of the paper is organized as follows. Section 2 derives the model and establishes its deterministic inertial-coupling limit. Section 3 presents the linear delayed spectrum, modal representation, nonlinear backbone, stochastic spectral clouds, and time-domain Monte Carlo evidence. Section 4 discusses the vibration-mechanism interpretation and limitations, and Section 5 concludes the paper.

## 2. Model

**Figure 1:**
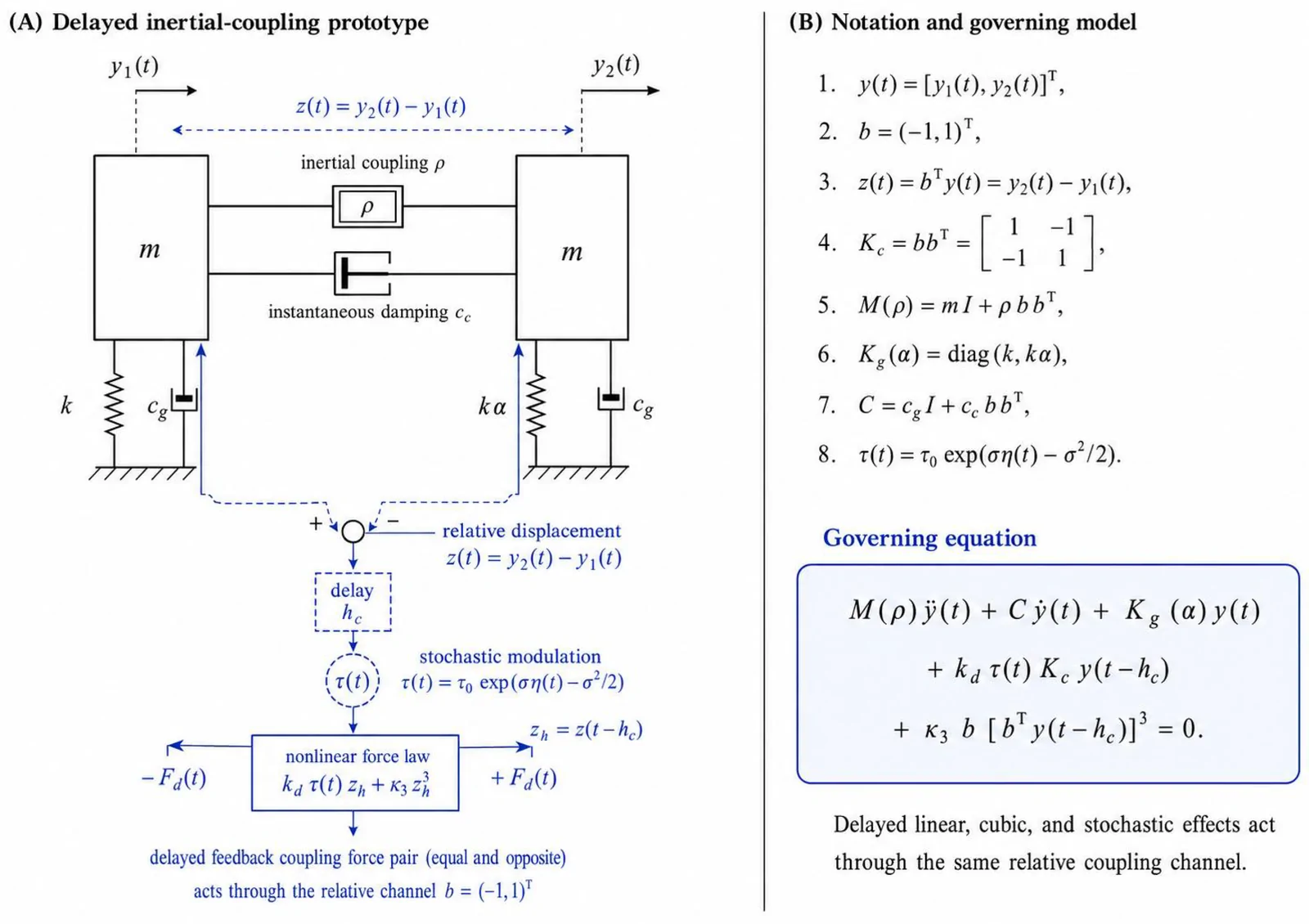
Inertially coupled prototype with delayed relative-coordinate feedback and governing notation. (**A**) The two primary masses are grounded by linear stiffness and damping. They are coupled through an instantaneous relative inertial channel. The delayed feedback acts on the relative displacement $z\left(t\right)=y_{2}\left(t\right)-y_{1}\left(t\right)$ after the delay $h_{c}$, and the stochastic modulation $\tau \left(t\right)=\tau_{0}\exp\left(\sigma \eta \left(t\right)-\sigma^{2}/2\right)$ multiplies the delayed linear part. The delayed linear and cubic forces form an equal-and-opposite pair through the relative channel. (**B**) The delay is introduced through the relative-coordinate-restoring channel, while the inertial coupling remains in $M\left(\rho \right)=mI+\rho bb^{T}$.

We begin by defining the mechanical prototype, fixing the sign convention, and deriving the delayed nonlinear stochastic equation used in the computations. The prototype and the notation are summarized in Figure 1.

### 2.1. Coordinates and Relative Coupling Channel

Let

(1) $$y\left(t\right)=\left[\begin{array}{c} y_{1}\left(t\right)\\ y_{2}\left(t\right) \end{array}\right],\;b=\left[\begin{array}{c} -1\\ 1 \end{array}\right],\;z\left(t\right)=b^{T}y\left(t\right)=y_{2}\left(t\right)-y_{1}\left(t\right).$$

The relative-displacement coupling matrix is

(2) $$K_{c}=bb^{T}=\left[\begin{array}{cc} 1 & -1\\ -1 & 1 \end{array}\right].$$

Then, $K_{c}y=bz.$ This convention fixes the force directions. If $z_{h}=z\left(t-h_{c}\right)>0$, the generalized delayed force

$$-k_{d}\tau \left(t\right)K_{c}y\left(t-h_{c}\right)=-k_{d}\tau \left(t\right)bz_{h}$$

has components ${\left(k_{d}\tau \left(t\right)z_{h},\;-k_{d}\tau \left(t\right)z_{h}\right)}^{T}$. Then, the delayed force pair is equal, opposite, and tends to reduce the delayed relative displacement. In addition, if we replace $k_{d}\tau \left(t\right)z_{h}$ by $\kappa_{3}z_{h}^{3}$, the cubic delayed force has the same direction convention.

### 2.2. Kinetic Energy, Elastic Energy, and Damping

The kinetic energy is

(3) $$T=\frac{1}{2}m\left({\dot{y}}_{1}^{2}+{\dot{y}}_{2}^{2}\right)+\frac{1}{2}\rho {\left({\dot{y}}_{2}-{\dot{y}}_{1}\right)}^{2}=\frac{1}{2}{\dot{y}}^{T}M\left(\rho \right)\dot{y},$$

with

(4) $$M\left(\rho \right)=mI+\rho bb^{T}=\left[\begin{array}{cc} m+\rho & -\rho \\ -\rho & m+\rho \end{array}\right].$$

For m>0 and ρ≥0, M(ρ) is positive definite, since its eigenvalues are *m* and m+2ρ. Thus, the inertial coupling changes the modal interaction without introducing an indefinite kinetic energy.

The grounding potential is

(5) $$V_{g}\left(y\right)=\frac{1}{2}ky_{1}^{2}+\frac{1}{2}k\alpha y_{2}^{2}=\frac{1}{2}y^{T}K_{g}\left(\alpha \right)y,$$

with

(6) $$K_{g}\left(\alpha \right)=\left[\begin{array}{cc} k & 0\\ 0 & k\alpha \end{array}\right].$$

The detuning parameter α is the unfolding parameter for mode veering. It can control the separation of the two grounded frequencies. The Rayleigh dissipation function is

(7) $$R=\frac{1}{2}c_{g}\left({\dot{y}}_{1}^{2}+{\dot{y}}_{2}^{2}\right)+\frac{1}{2}c_{c}{\left({\dot{y}}_{2}-{\dot{y}}_{1}\right)}^{2}=\frac{1}{2}{\dot{y}}^{T}C\dot{y},$$

where

(8) $$C=c_{g}I+c_{c}bb^{T}.$$

For $c_{g}\geq 0$ and $c_{c}\geq 0$, the damping matrix is positive semidefinite. Furthermore, it is positive definite when $c_{g}>0$.

### 2.3. Delayed Nonlinear Stochastic Coupling Force

Since

$$z_{h}=z\left(t-h_{c}\right)=b^{T}y\left(t-h_{c}\right),$$

the scalar force law in the relative channel is

(9) $$F_{d}\left(t\right)=k_{d}\tau \left(t\right)z_{h}+\kappa_{3}z_{h}^{3}.$$

Because the relative channel acts along $b={\left(-1,1\right)}^{T}$, the delayed generalized force applied to the two generalized coordinates is

(10) $$Q_{d}\left(t\right)=-bF_{d}\left(t\right)=-k_{d}\tau \left(t\right)K_{c}y\left(t-h_{c}\right)-\kappa_{3}b{\left[b^{T}y\left(t-h_{c}\right)\right]}^{3},$$

which is the precise mathematical form of the force pair shown in Figure 1.

Set the stochastic multiplier by

(11) $$\tau \left(t\right)=\tau_{0}\exp\left(\sigma \eta \left(t\right)-\frac{\sigma^{2}}{2}\right),$$

where η(t) is an Ornstein–Uhlenbeck process with stationary distribution N(0,1). Then,

$$\tau \left(t\right)>0,\;E\left[\tau \left(t\right)\right]=\tau_{0}$$

under stationary sampling. Thus, the stochastic term is a positive multiplicative modulation of the delayed coupling channel. The equilibrium y=0 is preserved without an external random force.

**Lemma 1** 
(Positive mean-preserving coupling multiplier)**.** *Assume that η(t) is sampled from its stationary distribution N(0,1) and define τ(t) by Equation (11). Then, τ(t)>0 almost surely and $E\left[\tau \left(t\right)\right]=\tau_{0}$. Moreover, the zero history is an invariant solution of the unforced stochastic delay equation.*

**Proof.**  The positivity follows from the exponential form. Since η∼N(0,1), $\exp\left(\sigma \eta \right)=\exp\left(\sigma^{2}/2\right)$, and $E\left[\tau \left(t\right)\right]=\tau_{0}\exp\left(-\sigma^{2}/2\right)\exp\left(\sigma^{2}/2\right)=\tau_{0}$. If y≡0 on the delay interval, then $z_{h}=0$, the delayed linear and cubic generalized forces vanish, and all terms in the governing equation are zero. □

### 2.4. Governing Equation

Let $L=T-V_{g}$. The Lagrange–d’Alembert equation with Rayleigh dissipation is

(12) $$\frac{d}{dt}\frac{\partial L}{\partial \dot{y}}-\frac{\partial L}{\partial y}+\frac{\partial R}{\partial \dot{y}}=Q_{d}\left(t\right).$$

Then, we have

(13) $$M\left(\rho \right)\ddot{y}\left(t\right)+C\dot{y}\left(t\right)+K_{g}\left(\alpha \right)y\left(t\right)=-k_{d}\tau \left(t\right)K_{c}y\left(t-h_{c}\right)-\kappa_{3}b{\left[b^{T}y\left(t-h_{c}\right)\right]}^{3}$$

with (3)–(10). It is equal to

(14) $$M\left(\rho \right)\ddot{y}\left(t\right)+C\dot{y}\left(t\right)+K_{g}\left(\alpha \right)y\left(t\right)+k_{d}\tau \left(t\right)K_{c}y\left(t-h_{c}\right)+\kappa_{3}b{\left[b^{T}y\left(t-h_{c}\right)\right]}^{3}=0.$$

This equation is the common starting point for the linear delayed spectrum, modal representation, nonlinear backbone, stochastic spectral clouds, and time-domain Monte Carlo simulations.

**Theorem 1** 
(Energy-derived delayed relative-coordinate model)**.** *Let m>0, ρ≥0, $c_{g},c_{c}\geq 0$, and the generalized delayed force be given by Equation (10). Then, the Lagrange–d’Alembert equation with Rayleigh dissipation can yield Equation (14). In addition, M(ρ) is positive definite and the relative-channel force pair is equal and opposite along the vector $b={\left(-1,1\right)}^{T}$.*

**Proof.**  We get $\partial T/\partial \dot{y}=M\left(\rho \right)\dot{y}$, $\partial V_{g}/\partial y=K_{g}\left(\alpha \right)y$, and $\partial R/\partial \dot{y}=C\dot{y}$ from Equations (3)–(7). Substituting these terms and Equation (10) into Equation (12) gives Equation (14). The eigenvalues of $M\left(\rho \right)=mI+\rho bb^{T}$ are *m* and m+2ρ, so M(ρ) is positive definite under the stated assumptions. Finally, $K_{c}y=b\left(b^{T}y\right)$, so the delayed generalized force has the two opposite components induced by the relative-coordinate direction *b*. □

Set $\kappa_{3}=0$, $\tau \left(t\right)=\tau_{0}$. The deterministic linear delayed backbone (14) is

(15) $$M\left(\rho \right)\ddot{y}\left(t\right)+C\dot{y}\left(t\right)+K_{g}\left(\alpha \right)y\left(t\right)+K_{d}y\left(t-h_{c}\right)=0,\;K_{d}=k_{d}\tau_{0}K_{c}.$$

Substituting $y\left(t\right)=\hat{y}e^{st}$, we have the characteristic equation

(16) $$\det\left[s^{2}M\left(\rho \right)+sC+K_{g}\left(\alpha \right)+K_{d}e^{-sh_{c}}\right]=0.$$

Equation (16) is used only as the linear delayed backbone. Nonlinear and stochastic analyses must instead use (14) or its amplitude-dependent and frozen-noise specializations.

### 2.5. Parameter Normalization and Numerical Values

The numerical experiments are formulated in nondimensional variables in order to isolate the effects of delayed relative-coordinate coupling, weak nonlinear stiffness, and stochastic coupling modulation without tying the benchmark to one particular engineering device. To make the physical interpretation explicit, the nondimensional parameters can be mapped back to dimensional mechanical quantities through the reference mass, time, and displacement scales $M_{0}$, $T_{0}$, and $L_{0}$, respectively.

Let dimensional quantities be denoted by a tilde and define

(17) $$\tilde{y}=L_{0}y,\;\tilde{t}=T_{0}t,\;{\tilde{h}}_{c}=T_{0}h_{c}.$$

Starting from the dimensional mechanical equation and dividing by the reference force scale $M_{0}L_{0}/T_{0}^{2}$ gives

(18) $$M=\frac{\tilde{M}}{M_{0}},\;C=\frac{T_{0}\tilde{C}}{M_{0}},\;K_{g}=\frac{T_{0}^{2}{\tilde{K}}_{g}}{M_{0}},$$

and

(19) $$k_{d}=\frac{T_{0}^{2}}{M_{0}}{\tilde{k}}_{d},\;\kappa_{3}=\frac{T_{0}^{2}L_{0}^{2}}{M_{0}}{\tilde{\kappa }}_{3}.$$

Equivalently, the dimensional scalar parameters corresponding to the parameterization used in this work are

(20) $$\begin{array}{cccc} \tilde{m} & =M_{0}m,\; & \tilde{\rho } & =M_{0}\rho ,\\ {\tilde{c}}_{g} & =\frac{M_{0}}{T_{0}}c_{g},\; & {\tilde{c}}_{c} & =\frac{M_{0}}{T_{0}}c_{c},\\ \tilde{k} & =\frac{M_{0}}{T_{0}^{2}}k,\; & {\tilde{k}}_{d} & =\frac{M_{0}}{T_{0}^{2}}k_{d},\\ {\tilde{\kappa }}_{3} & =\frac{M_{0}}{T_{0}^{2}L_{0}^{2}}\kappa_{3},\; & {\tilde{A}}_{z} & =L_{0}A_{z}. \end{array}$$

The corresponding dimensional delay, integration step, simulation horizon, and OU correlation rate are

(21) $${\tilde{h}}_{c}=T_{0}h_{c},\;\tilde{\Delta t}=T_{0}\Delta t,\;\tilde{T}=T_{0}T,\;\tilde{\theta }=\frac{\theta }{T_{0}}.$$

Likewise, a dimensionless characteristic root s=μ+iω corresponds to the dimensional root:

(22) $$\tilde{s}=\frac{s}{T_{0}},\;\tilde{\omega }=\frac{\omega }{T_{0}}.$$

The quantities α, $\tau_{0}$, and σ remain dimensionless: α is a grounding-stiffness ratio, $\tau_{0}$ is the nominal multiplicative coupling level, and σ controls the dimensionless lognormal modulation intensity. The Monte Carlo sample number $N_{\mathrm{MC}}$ is also dimensionless.

The nondimensional values used throughout the paper are listed in Table 1. These values define a reproducible mechanism-oriented benchmark; Equations (17)–(22) allow the same results to be translated consistently to a dimensional structural scale once $M_{0}$, $T_{0}$, and $L_{0}$ are specified for a particular application. Sensitivity checks for the stochastic time-domain statistics are reported in the Supplementary Materials.

The baseline time-domain Monte Carlo results use $N_{\mathrm{MC}}=256$ trajectories per grid point. For the quantitative energy-contribution audit reported below, the same time-domain parameters, Δt=0.02, T=100, and θ=0.40, are retained, while the ensemble size is increased to $N_{\mathrm{MC}}=1000$ to reduce Monte Carlo sampling uncertainty.

**Table 1:** Nondimensional benchmark parameters used in the numerical studies. Their dimensional counterparts follow from Equations (20) and (21).

Symbol	Value	Role	Used in
*m*	1.0	Reference mass	All figures
ρ	0.05	Inertial-coupling coefficient	All figures
*k*	1.0	Reference grounding stiffness	All figures
α	Scanned	Detuning stiffness ratio	Figure 2, Figure 3, Figure 4, Figure 5, Figure 6 and Figure 7
$c_{g}$	0.08	Grounding damping	Figure 2, Figure 3, Figure 4, Figure 5, Figure 6 and Figure 7
$c_{c}$	0.004	Relative-channel damping	Figure 2, Figure 3, Figure 4, Figure 5, Figure 6 and Figure 7
$k_{d}$	0.12	Delayed linear coupling coefficient	Figure 2, Figure 3, Figure 4, Figure 5, Figure 6 and Figure 7
$\tau_{0}$	1.0	Nominal coupling multiplier	Figure 2, Figure 3, Figure 4, Figure 5, Figure 6 and Figure 7
$h_{c}$	0–0.52	Coupling delay	Figure 2, Figure 3, Figure 4, Figure 5, Figure 6 and Figure 7
$\kappa_{3}$	0.20	Delayed cubic coupling coefficient	Figure 5, Figure 6 and Figure 7
$A_{z}$	0–0.55	Relative-displacement backbone amplitude	Figure 5 and Figure 6
σ	0–0.36	Stochastic coupling intensity	Figure 6 and Figure 7
θ	0.40	OU correlation-rate parameter	Time-domain MC
Δt	0.02	Time-domain integration step	Time-domain MC
*T*	100	Simulation horizon	Time-domain MC
$N_{\mathrm{MC}}$	256	Monte Carlo trajectories per grid point	Time-domain MC

The dimensional interpretation of the parameters follows directly from the mechanical prototype. The quantities $\tilde{m}$ and $\tilde{\rho }$ have units of mass and represent, respectively, the grounding mass scale and the inertial coupling associated with relative motion. The coefficients ${\tilde{c}}_{g}$ and ${\tilde{c}}_{c}$ have units of damping and represent grounding and relative-motion dissipation. The two grounding stiffnesses are $\tilde{k}$ and $\alpha \tilde{k}$, whereas ${\tilde{k}}_{d}\tau_{0}$ is the nominal delayed linear stiffness in the relative-coordinate channel. The cubic coefficient ${\tilde{\kappa }}_{3}$ has units of force per displacement cubed and acts through the same delayed relative-motion channel. Hence, the prototype can be interpreted as a generic inertially coupled attachment or delayed vibration-control element rather than as a calibrated model of one specific device.

**Assumption 1** 
(Benchmark parameter regime)**.** *Unless otherwise stated, the numerical study uses the dimensionless parameter set in Table 1, with m>0, ρ≥0, $c_{g},c_{c}\geq 0$, $k_{d}>0$, $\kappa_{3}\geq 0$, and $h_{c}\geq 0$. The DDE is interpreted as a retarded equation with a continuous prescribed history on $\left[-h_{c},0\right]$.*

### 2.6. Deterministic Inertial-Coupling Limit

The present model contains the deterministic inertial-coupling veering model as a limiting case. Setting

$$h_{c}=0,\;\kappa_{3}=0,\;\sigma =0,\;\tau \left(t\right)=\tau_{0}$$

in (14), we have

(23) $$M\left(\rho \right)\ddot{y}\left(t\right)+C\dot{y}\left(t\right)+\left[K_{g}\left(\alpha \right)+k_{d}\tau_{0}K_{c}\right]y\left(t\right)=0.$$

Suppressing damping, we get

(24) $$M\left(\rho \right)\ddot{y}\left(t\right)+K_{\det}\left(\alpha ,\tau \right)y\left(t\right)=0,\;K_{\det}\left(\alpha ,\tau \right)=K_{g}\left(\alpha \right)+k\tau K_{c},$$

where

$$\tau =\frac{k_{d}\tau_{0}}{k}.$$

Equivalently,

(25) $$K_{\det}\left(\alpha ,\tau \right)=k\left[\begin{array}{cc} 1+\tau & -\tau \\ -\tau & \alpha +\tau \end{array}\right],\;M\left(\rho \right)=mI+\rho bb^{T}.$$

This is the instantaneous stiffness–inertial coupled oscillator used as the deterministic reference. The subsequent analysis unfolds this baseline through coupling delay $h_{c}$, delayed cubic coupling $\kappa_{3}$, and positive multiplicative stochastic modulation.

## 3. Results

Unless otherwise stated, the plotted curves and statistics are computed from the governing equations rather than fitted to graphical trends. The central question is how delayed stochastic nonlinear relative-coordinate coupling reshapes complex-root veering, branch exchange, and finite-time branch residence in an inertially coupled oscillator.

### 3.1. Linear Delayed Veering and Delay-Sensitive Stability

Around the equilibrium y=0, the Fréchet derivative of the delayed cubic term is zero. Suppressing the cubic term and freezing the stochastic multiplier at its nominal mean, we have

(26) $$M\ddot{y}\left(t\right)+C\dot{y}\left(t\right)+K_{g}\left(\alpha \right)y\left(t\right)+K_{d}y\left(t-h_{c}\right)=0,\;K_{d}=k_{d}\tau_{0}K_{c}.$$

With $x\left(t\right)={\left(y^{T}\left(t\right),{\dot{y}}^{T}\left(t\right)\right)}^{T}$, this equation can be written as the retarded first-order DDE

(27) $$\dot{x}\left(t\right)=A_{0}\left(\alpha \right)x\left(t\right)+A_{1}x\left(t-h_{c}\right),$$

where

(28) $$A_{0}\left(\alpha \right)=\left[\begin{array}{cc} 0 & I\\ -M^{-1}K_{g}\left(\alpha \right) & -M^{-1}C \end{array}\right],\;A_{1}=\left[\begin{array}{cc} 0 & 0\\ -M^{-1}K_{d} & 0 \end{array}\right].$$

The characteristic equation is

(29) $$\Delta \left(s;\alpha ,h_{c}\right)=\det\left\{sI-A_{0}\left(\alpha \right)-A_{1}e^{-sh_{c}}\right\}=0,$$

or equivalently

(30) $$\det\{s^{2}M+sC+K_{g}\left(\alpha \right)+K_{d}e^{-sh_{c}}\}=0.$$

Because the delayed coupling matrix $K_{c}=bb^{T}$ has rank one, the characteristic determinant in Equation (30) can be reduced exactly to a single-delay quasi-polynomial of the form

(31) $$F\left(s;\alpha ,h_{c}\right)=P\left(s;\alpha \right)+de^{-sh_{c}}R\left(s;\alpha \right)=0,\;d=k_{d}\tau_{0},$$

where

(32) $$P\left(s;\alpha \right)=p_{4}s^{4}+p_{3}s^{3}+p_{2}s^{2}+p_{1}s+p_{0},$$

with

(33) $$\begin{array}{cc} p_{4} & =m(m+2\rho ),\\ p_{3} & =2\left[\left(m+\rho \right)c_{g}+mc_{c}\right],\\ p_{2} & =c_{g}^{2}+2c_{g}c_{c}+\left(m+\rho \right)k\left(1+\alpha \right),\\ p_{1} & =\left(c_{g}+c_{c}\right)k\left(1+\alpha \right),\\ p_{0} & =k^{2}\alpha , \end{array}$$

and

(34) $$R\left(s;\alpha \right)=2ms^{2}+2c_{g}s+k\left(1+\alpha \right).$$

**Proposition 1** 
(Imaginary-axis-crossing criterion)**.** *For a fixed detuning parameter α>0, a nonzero imaginary-axis characteristic root s=iω, ω>0, can occur only if*

(35) $$P_{R}^{2}\left(\omega \right)+P_{I}^{2}\left(\omega \right)=d^{2}\left[R_{R}^{2}\left(\omega \right)+R_{I}^{2}\left(\omega \right)\right],$$

*where*

(36) $$\begin{array}{cc} P_{R}\left(\omega \right) & =p_{4}\omega^{4}-p_{2}\omega^{2}+p_{0},\\ P_{I}\left(\omega \right) & =-p_{3}\omega^{3}+p_{1}\omega ,\\ R_{R}\left(\omega \right) & =k\left(1+\alpha \right)-2m\omega^{2},\\ R_{I}\left(\omega \right) & =2c_{g}\omega . \end{array}$$

*For every positive solution ω of Equation (35), the corresponding critical delays satisfy*

(37) $$\cos\left(\omega h_{c}\right)=-\frac{P_{R}R_{R}+P_{I}R_{I}}{d(R_{R}^{2}+R_{I}^{2})},$$

*and*

(38) $$\sin\left(\omega h_{c}\right)=\frac{P_{I}R_{R}-P_{R}R_{I}}{d(R_{R}^{2}+R_{I}^{2})}.$$

*Hence, the admissible critical-delay sequence can be obtained from*

(39) $$h_{c,n}=\frac{\mathrm{atan}2(S,C)+2n\pi }{\omega },\;n\in Z,$$

*where C and S denote the right-hand sides of Equations (37) and (38), respectively, with the phase chosen so that $h_{c,n}>0$.*

**Proof.**  Substituting s=iω into Equation (31) gives

$$P_{R}+iP_{I}+d\left[\cos\left(\omega h_{c}\right)-i\sin\left(\omega h_{c}\right)\right]\left(R_{R}+iR_{I}\right)=0.$$

Separating real and imaginary parts and eliminating the delay phase gives Equation (35). Solving the same two equations for $\cos(\omega h_{c})$ and $\sin(\omega h_{c})$ yields Equations (37) and (38). Because Equation (35) depends only on even powers of ω after squaring, setting $x=\omega^{2}$ converts it into a polynomial equation in *x*. Thus, the candidate crossing frequencies can be obtained without a direct search over the complex *s*-plane. □

For a simple imaginary-axis root, the crossing direction can be determined by implicit differentiation of Equation (31):

(40) $$\frac{ds}{dh_{c}}=\frac{d\;s\;e^{-sh_{c}}R\left(s\right)}{P^{\prime }\left(s\right)+de^{-sh_{c}}\left[R^{\prime }\left(s\right)-h_{c}R\left(s\right)\right]}.$$

The sign of Re(ds/dhc) at s=iω determines the local crossing direction. Since the system at $h_{c}=0$ has positive-definite mass and stiffness matrices and positive-definite damping for the parameter range considered here, its linearized deterministic reference state is asymptotically stable. The smallest positive critical delay associated with an outward simple crossing therefore provides the first analytical delay-stability boundary for a fixed α. The criterion concerns the deterministic linearized DDE and does not constitute a global stability result for the nonlinear stochastic system.

The two veering-related positive-imaginary roots are written as

(41) $$s_{j}\left(\alpha ,h_{c}\right)=\mu_{j}\left(\alpha ,h_{c}\right)+i\omega_{j}\left(\alpha ,h_{c}\right),\;j=1,2.$$

They are continued from the two instantaneous vibratory eigenvalues. The veering center and frequency gap are

(42) $$\alpha_{\mathrm{cov}}\left(h_{c}\right)=\underset{\alpha }{\arg\min}\;\left[\omega_{2}\left(\alpha ,h_{c}\right)-\omega_{1}\left(\alpha ,h_{c}\right)\right],\;g_{\omega }\left(h_{c}\right)=\min_{\alpha }\;\left[\omega_{2}\left(\alpha ,h_{c}\right)-\omega_{1}\left(\alpha ,h_{c}\right)\right].$$

We use the branch-tracked growth indicator:

(43) mtr(α,hc)=maxj=1,2Resj(α,hc).

**Definition 1** 
(Tracked veering pair and associated metrics)**.** *The pair $s_{1},s_{2}$ is called the tracked veering pair on a parameter interval $I_{\alpha }$, when it is obtained by continuous continuation from the two positive-imaginary vibratory roots at the lower end of $I_{\alpha }$, ordered by positive imaginary part, and realizes the minimum gap $\omega_{2}-\omega_{1}$ within the tested interval. The quantities $\alpha_{\mathrm{cov}}$, $g_{\omega }$, and $m_{\mathrm{tr}}$ are the veering-center, minimum-gap, and tracked-growth metrics of this pair.*

Because the DDE has infinitely many roots, $m_{\mathrm{tr}}$ is a tracked veering-branch indicator. It is used together with the independent rightmost-spectrum validation and is not a stand-alone proof of complete DDE stability.

**Proposition 2** 
(Local continuation of simple veering roots)**.** Fix a compact parameter rectangle in $\left(\alpha ,h_{c}\right)$. If a veering-related characteristic root $s_{*}$ satisfies $\Delta (s_{*};\alpha_{*},h_{c*})=0$ and $\partial_{s}\Delta \left(s_{*};\alpha_{*},h_{c*}\right)\neq 0$, then there exists a neighborhood of $\left(\alpha_{*},h_{c*}\right)$ in which this root is represented by a unique smooth branch $s(\alpha ,h_{c})$. Consequently, the quantities ωj=Imsj, μj=Resj, and the branch-tracked metrics $\alpha_{\mathrm{cov}}$, $g_{\omega }$, and $m_{\mathrm{tr}}$ are well-defined as long as the two tracked branches remain simple and separated from branch-switching ambiguity.

**Proof.**  For fixed matrices, $\Delta (s;\alpha ,h_{c})$ is analytic in *s* and smooth in α and $h_{c}$. The assertion follows from the implicit function theorem applied to $\Delta (s;\alpha ,h_{c})=0$. Then, the definitions of veering metrics follow by taking the imaginary and real parts of the continued branches and by minimizing the branch gap over the tested detuning interval. □

**Figure 2:**
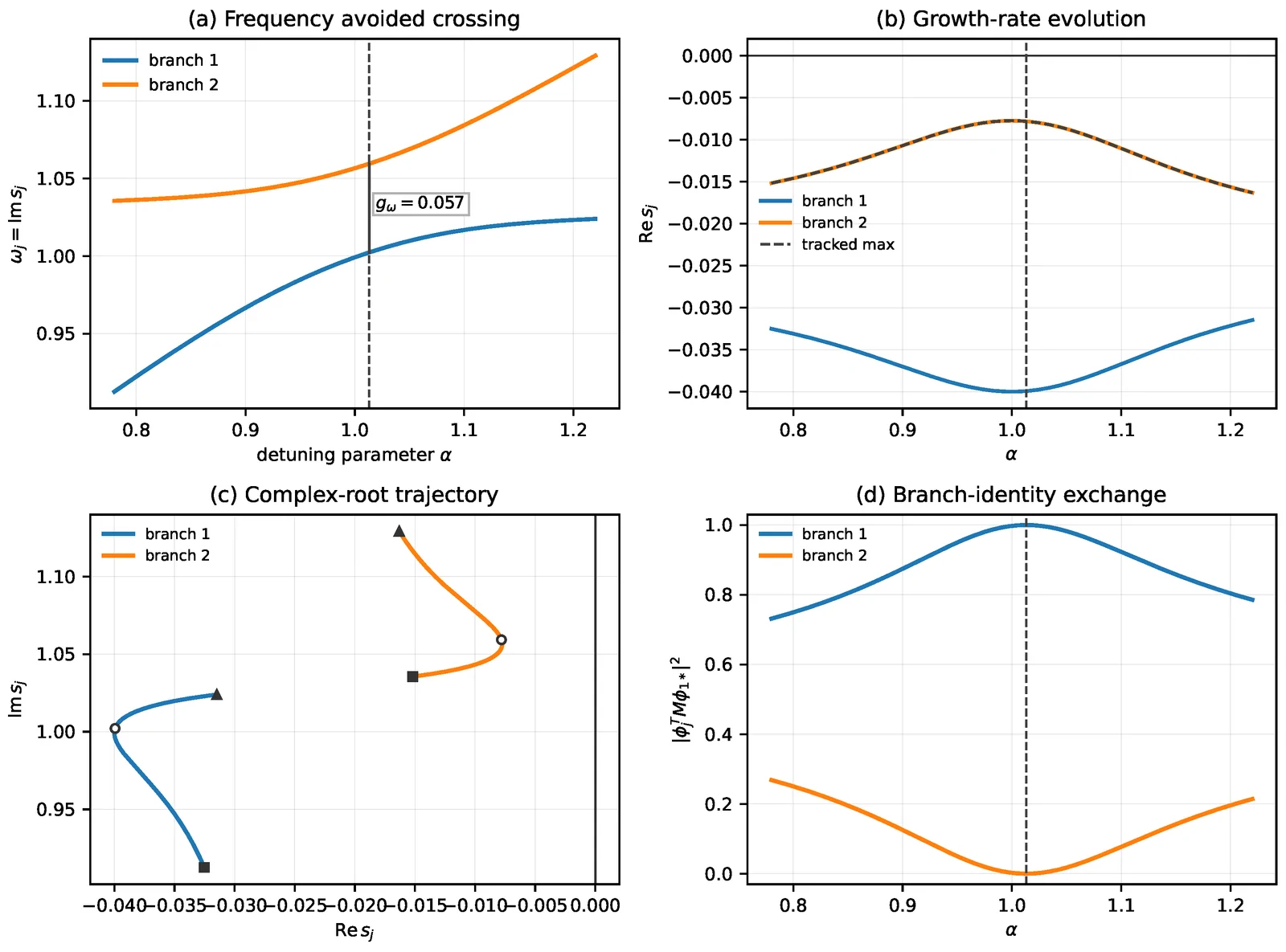
Deterministic complex-root veering at the representative delay $h_{c}=0.30$. The two positive-imaginary characteristic roots $s_{j}\left(\alpha \right)=\mu_{j}\left(\alpha \right)+i\omega_{j}\left(\alpha \right)$ are continued from the undelayed eigenvalues and tracked as functions of the detuning parameter α. The frequency gap attains its minimum at $\alpha_{\mathrm{cov}}\approx 1.0122$, with $g_{\omega }\approx 0.0571$, while the real parts quantify the corresponding tracked growth margins. The complex-root locus and branch-identity indicator show that the avoided frequency crossing is accompanied by exchange of modal character, rather than being only a graphical crossing of two frequency curves. The vertical dashed line in panels (**a**,**b**,**d**) marks the veering center $\alpha_{\mathrm{cov}}$, while the black dashed curve in panel (**b**) denotes the tracked growth indicator mtr=maxj=1,2Resj.

Figure 2 traces the veering structure through complex characteristic roots of the delayed equation. At $h_{c}=0.30$, the two imaginary parts form an avoided crossing, and the branch-identity indicator confirms exchange of modal character near the minimum gap. The real parts of the same branches provide the tracked growth margin associated with the veering core, rather than a separate stability diagnostic.

**Figure 3:**
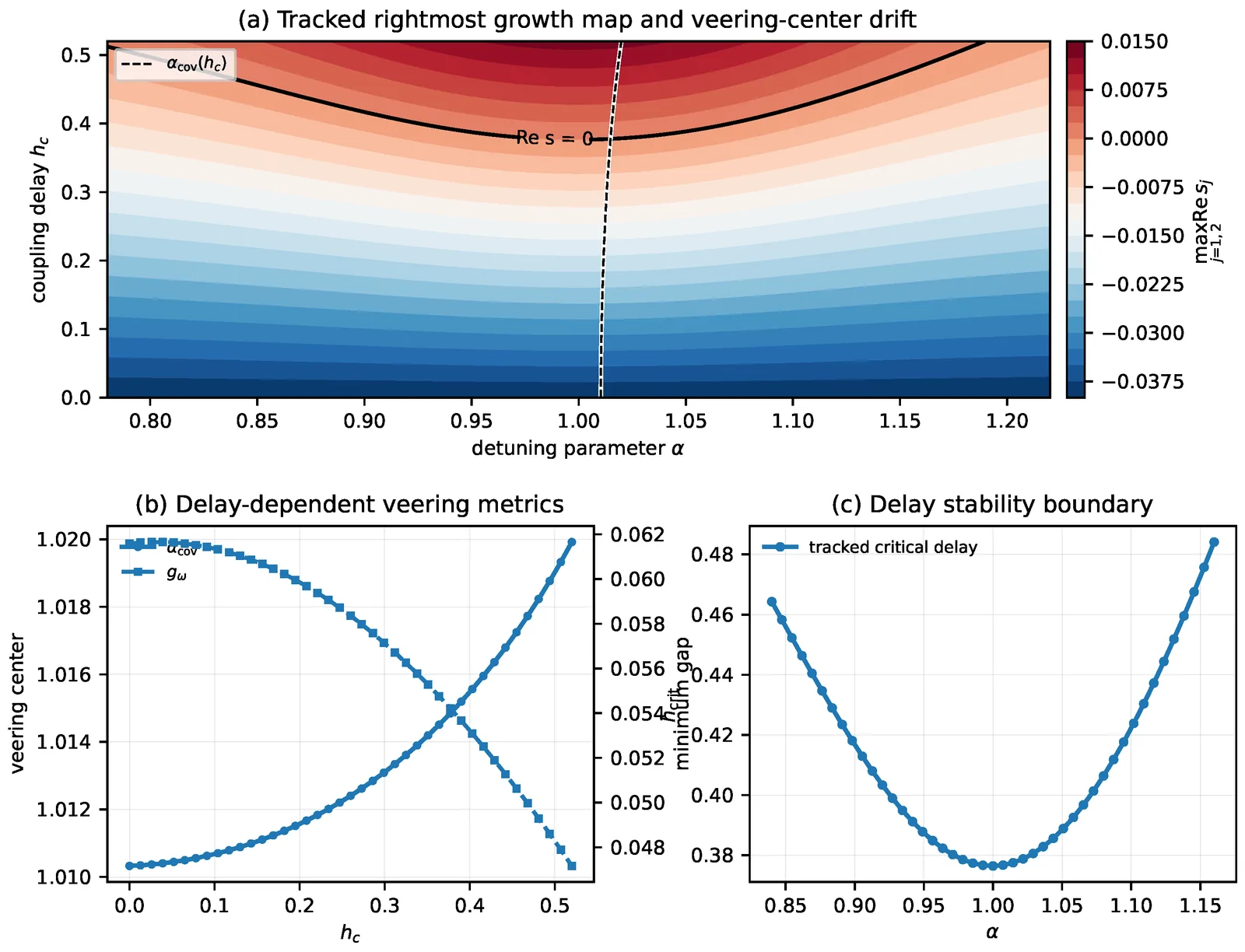
Delay-induced drift of the veering core and erosion of the spectral stability margin. Panel (**a**) maps the tracked growth indicator mtr(α,hc)=maxj=1,2Resj(α,hc) over the $\left(\alpha ,h_{c}\right)$ plane and overlays the zero-growth contour together with the veering-center trajectory $\alpha_{\mathrm{cov}}\left(h_{c}\right)$. Panel (**b**) shows the delay dependence of the veering center $\alpha_{\mathrm{cov}}\left(h_{c}\right)$ and the minimum frequency gap $g_{\omega }\left(h_{c}\right)$. Panel (**c**) reports the critical-delay boundary $h_{\mathrm{crit}}\left(\alpha \right)$ obtained from the imaginary-axis-crossing criterion.

Figure 3 extends the delay-dependent veering analysis over the $\left(\alpha ,h_{c}\right)$ plane. Increasing delay shifts the veering core toward larger detuning and reduces the minimum frequency gap. Over $h_{c}\in \left[0,0.52\right]$, $\alpha_{\mathrm{cov}}$ increases from approximately 1.0103 to 1.0199, while $g_{\omega }$ decreases from approximately 0.0616 to 0.0472. The tracked growth indicator $m_{\mathrm{tr}}$ approaches and crosses zero as the delay is increased. The critical-delay curve in Figure 3c is determined from the analytical imaginary-axis crossing criterion derived above. Independent multi-start quasi-polynomial root searches and Chebyshev-collocation calculations are used subsequently to validate this analytical boundary and the associated rightmost-root classification.

**Figure 4:**
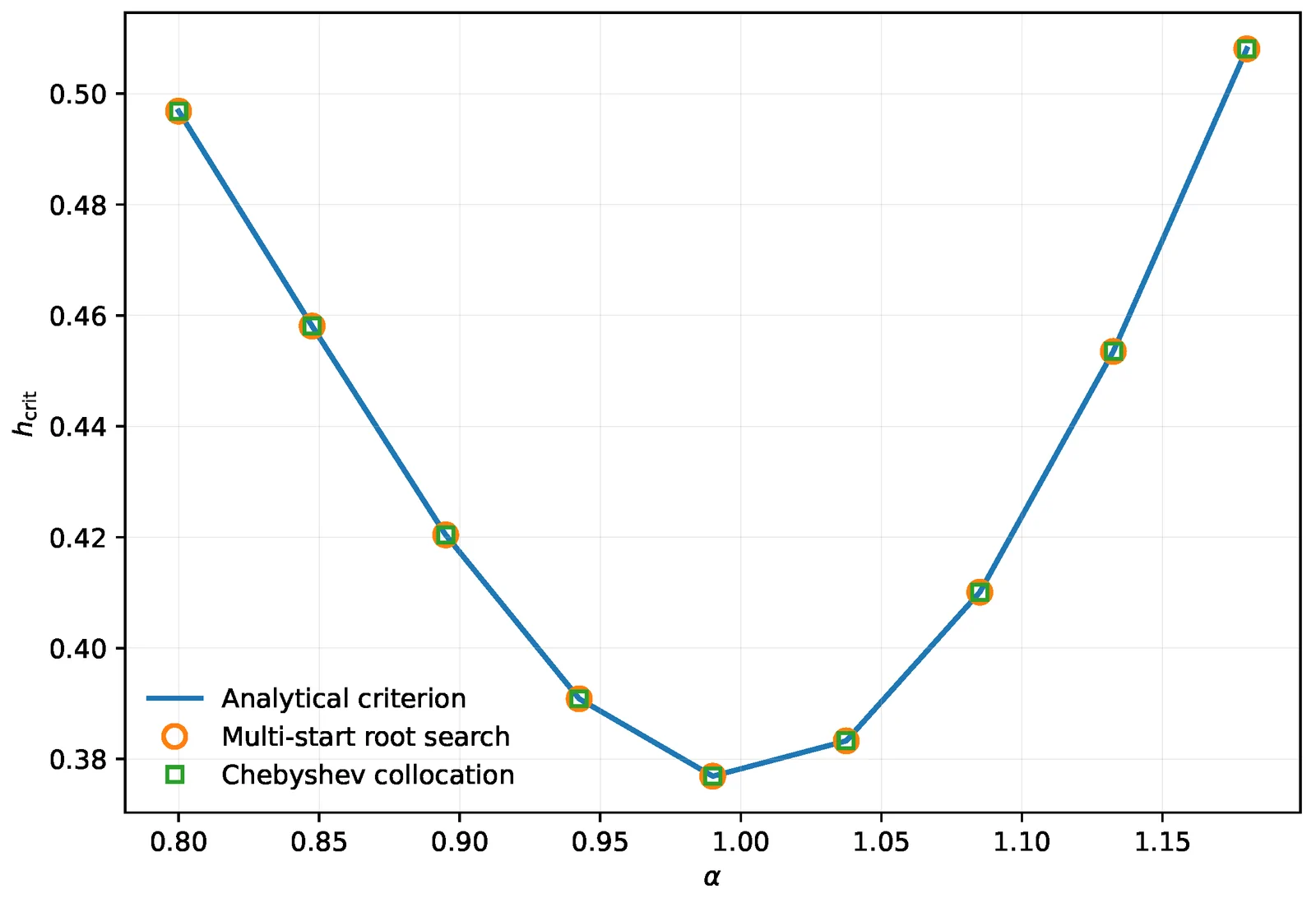
Validation of the analytical delay-stability boundary. The critical delay obtained from the imaginary-axis-crossing criterion is compared with independent direct multi-start quasi-polynomial root searches and Chebyshev-collocation approximations of the DDE generator over 0.80≤α≤1.18. The three results are practically indistinguishable over the comparison interval.

Figure 4 shows that the analytical and numerical critical-delay boundaries agree to high precision. Over the complete comparison grid, the maximum relative deviation from the analytical boundary is $5.63\times 10^{-10}$ for the direct multi-start calculation and $1.74\times 10^{-10}$ for the Chebyshev-collocation calculation. Thus, the loss of spectral stability detected numerically corresponds to the imaginary-axis crossing predicted by the analytical criterion within the investigated parameter range.

To assess the numerical convergence and reproducibility of the two validation procedures, additional calculations were performed at α=0.82, 1.00, and 1.16. For the Chebyshev approximation, the collocation order was increased through N=3,4,5,6,8,10,12,16. For the direct root search, four progressively enlarged multi-start settings were considered: MS1 used 27 seeds over Res∈[−0.5,0.15] and Ims∈[0,4]; MS2 used 85 seeds over Res∈[−1.0,0.20] and Ims∈[0,8]; MS3 used 175 seeds over Res∈[−1.5,0.25] and Ims∈[0,12]; and MS4 used 297 seeds over Res∈[−2.0,0.30] and Ims∈[0,16].

**Figure 5:**
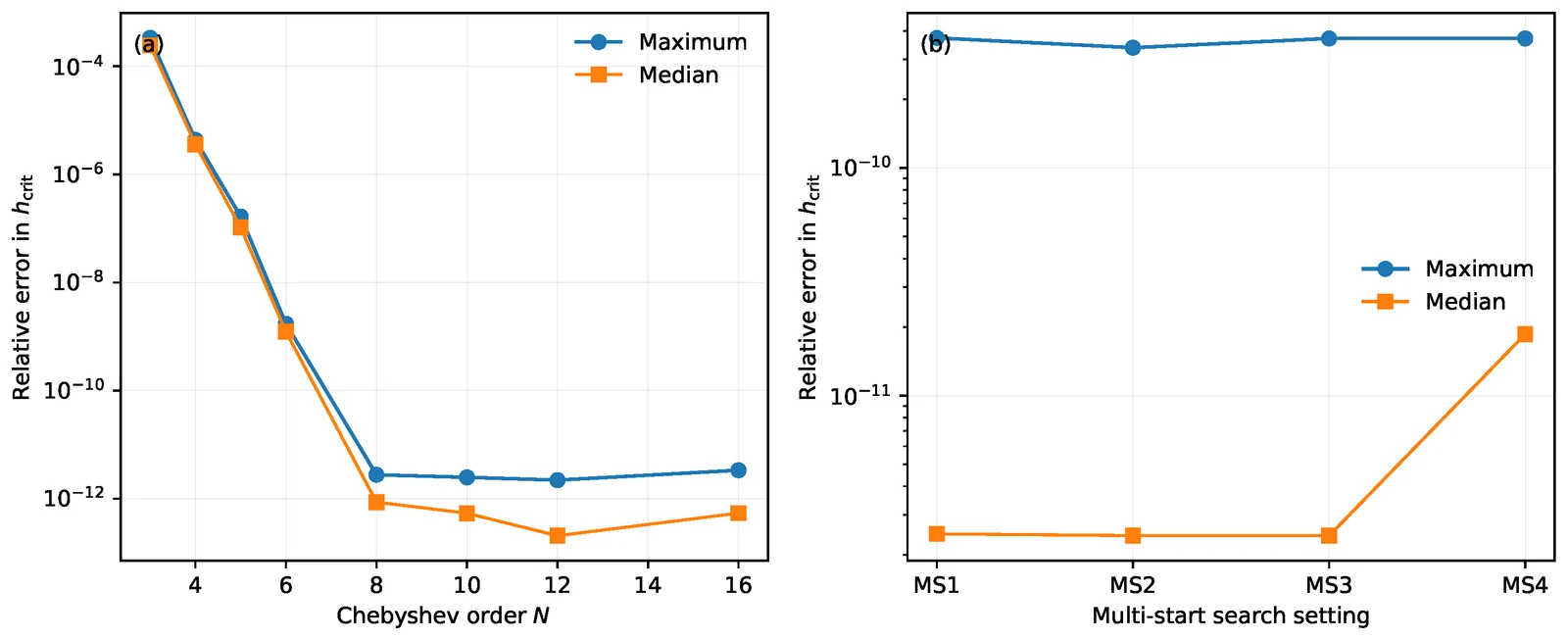
Numerical convergence and search-setting robustness of the characteristic-root calculations. (**a**) Maximum and median relative errors in $h_{\mathrm{crit}}$ for the Chebyshev-collocation approximation as the collocation order *N* is increased. (**b**) Maximum and median relative errors obtained with the progressively enlarged multi-start settings MS1–MS4. The analytical imaginary-axis-crossing result is used as the independent reference in both panels.

As shown in Figure 5a, the Chebyshev approximation exhibits rapid convergence with increasing collocation order. The maximum relative error in $h_{\mathrm{crit}}$ decreases from $3.35\times 10^{-4}$ at N=3 to $4.34\times 10^{-6}$ at N=4, $1.66\times 10^{-7}$ at N=5, and $1.72\times 10^{-9}$ at N=6. At N=8, the maximum error reaches $2.76\times 10^{-12}$, after which it remains at approximately the $10^{-12}$ level, indicating that the remaining discrepancy is dominated by numerical root-location and floating-point tolerances.

The multi-start calculation is assessed differently because it does not possess a discretization order analogous to the Chebyshev approximation. Figure 5b therefore examines robustness with respect to the search window and seed density. For all four settings, the maximum relative error remains below $3.8\times 10^{-10}$, while the maximum spread of $h_{\mathrm{crit}}$ among the four settings is only $1.32\times 10^{-11}$. Thus, further enlargement of the complex search region and the number of initial guesses does not materially alter the identified rightmost-root crossing.

**Numerical Finding 1**  (Analytical and numerical stability-boundary consistency)**.** *For the parameter set in Table 1, increasing $h_{c}$ shifts the veering center to larger detuning, decreases the minimum frequency gap, and eventually produces an imaginary-axis characteristic-root crossing. The analytical crossing condition determines the critical delay of the deterministic linearized DDE, while direct multi-start root searches and Chebyshev-collocation calculations independently reproduce the same boundary. The Chebyshev approximation converges rapidly with increasing collocation order, and the multi-start result remains insensitive to substantial enlargement of the seed-search configuration. These statements concern the local spectral boundary of the deterministic linearized DDE and do not constitute a global stability theorem for the nonlinear stochastic system.*

### 3.2. Fixed-Reference Modal-Coordinate Mechanism

The modal formulation is used to expose the branch-interaction mechanism, not to introduce a lossy truncation. Let $\alpha_{*}$ be the veering-core detuning for the conservative undelayed reference operator. Define

(44) $$\left[K_{g}\left(\alpha_{*}\right)+K_{d}\right]\phi_{j_{*}}=\omega_{j_{*}}^{2}M\phi_{j_{*}},\;\Phi_{*}^{T}M\Phi_{*}=I.$$

Since the physical prototype has two generalized coordinates,

$$y\left(t\right)=\Phi_{*}q\left(t\right),\;q\left(t\right)=\Phi_{*}^{T}My\left(t\right),$$

defines a nonsingular change in coordinates in the two-dimensional physical-coordinate space. This coordinate transformation is algebraically exact at the level of the physical equations because no physical coordinate is discarded. However, it should not be interpreted as a complete modal decomposition of the infinite-dimensional DDE state space or as a diagonalization of the delay operator. Substituting $y\left(t\right)=\Phi_{*}q\left(t\right)$ into Equation (26) and premultiplying by $\Phi_{*}^{T}$ gives

(45) $$M_{q}\ddot{q}\left(t\right)+C_{q}\dot{q}\left(t\right)+K_{q}\left(\alpha \right)q\left(t\right)+K_{d,q}q\left(t-h_{c}\right)=0,$$

where

(46) $$M_{q}=\Phi_{*}^{T}M\Phi_{*},\;C_{q}=\Phi_{*}^{T}C\Phi_{*},\;K_{q}\left(\alpha \right)=\Phi_{*}^{T}K_{g}\left(\alpha \right)\Phi_{*},\;K_{d,q}=\Phi_{*}^{T}K_{d}\Phi_{*}.$$

The corresponding characteristic matrix is

(47) $$D_{q}\left(s;\alpha ,h_{c}\right)=\Phi_{*}^{T}\left[s^{2}M+sC+K_{g}\left(\alpha \right)+K_{d}e^{-sh_{c}}\right]\Phi_{*}.$$

Only the conservative reference operator $K_{q}\left(\alpha_{*}\right)+K_{d,q}$ is diagonal by construction. In general, $C_{q}$, $K_{q}\left(\alpha \right)$ away from $\alpha_{*}$, and $K_{d,q}$ are not simultaneously diagonal, so $D_{q}\left(s;\alpha ,h_{c}\right)$ retains off-diagonal terms. In particular, the delayed matrix $K_{d,q}$ is not assumed to be diagonal. These retained off-diagonal terms are the coordinate-level quantities used here to expose branch interaction near veering. Importantly, the fixed-reference transformation does not replace the infinite-dimensional spectral problem of the DDE. The characteristic roots are still determined by the full quasi-polynomial

(48) $$\det\left[s^{2}M+sC+K_{g}\left(\alpha \right)+K_{d}e^{-sh_{c}}\right]=0,$$

and the exponential factor $e^{-sh_{c}}$, together with the associated infinite characteristic spectrum, is retained. The role of $\Phi_{*}$ is therefore to provide a physically interpretable coordinate representation, rather than a complete modal diagonalization of the delayed system.

**Proposition 3** 
(Fixed-reference modal-coordinate equivalence)**.** Let $\Phi_{*}\in R^{2\times 2}$ be the mass-normalized reference modal matrix defined above. Then, $\Phi_{*}$ is nonsingular, and the coordinate transformation

$$y=\Phi_{*}q,\;q=\Phi_{*}^{T}My,$$

*maps the two physical-coordinate equations to Equation (45) without discarding either physical coordinate. Moreover,*

(49) $$D_{q}\left(s;\alpha ,h_{c}\right)=\Phi_{*}^{T}D\left(s;\alpha ,h_{c}\right)\Phi_{*},$$

*so*

(50) $$\det D_{q}=\det{\left(\Phi_{*}\right)}^{2}\det D.$$

Because $\det(\Phi_{*})\neq 0$, the physical-coordinate and fixed-reference modal-coordinate formulations have identical characteristic roots. This algebraic equivalence does not imply that $D_{q}$ is diagonal or that the infinite-dimensional DDE generator has been reduced to two spectral modes.

**Proof.**  The positive definiteness of *M* and the mass normalization $\Phi_{*}^{T}M\Phi_{*}=I$ imply that $\Phi_{*}$ is nonsingular. Substitution of $y=\Phi_{*}q$ into the physical-coordinate DDE followed by premultiplication by $\Phi_{*}^{T}$ gives Equation (45). The characteristic matrices are therefore related by the nonsingular congruence $D_{q}=\Phi_{*}^{T}D\Phi_{*}$, from which $\det D_{q}=\det{\left(\Phi_{*}\right)}^{2}\det D$ follows. Hence, both formulations have the same zeros of the characteristic determinant. The transformed matrices nevertheless retain their off-diagonal delayed, damped, and detuned terms, and the exponential delay dependence is unchanged. □

**Figure 6:**
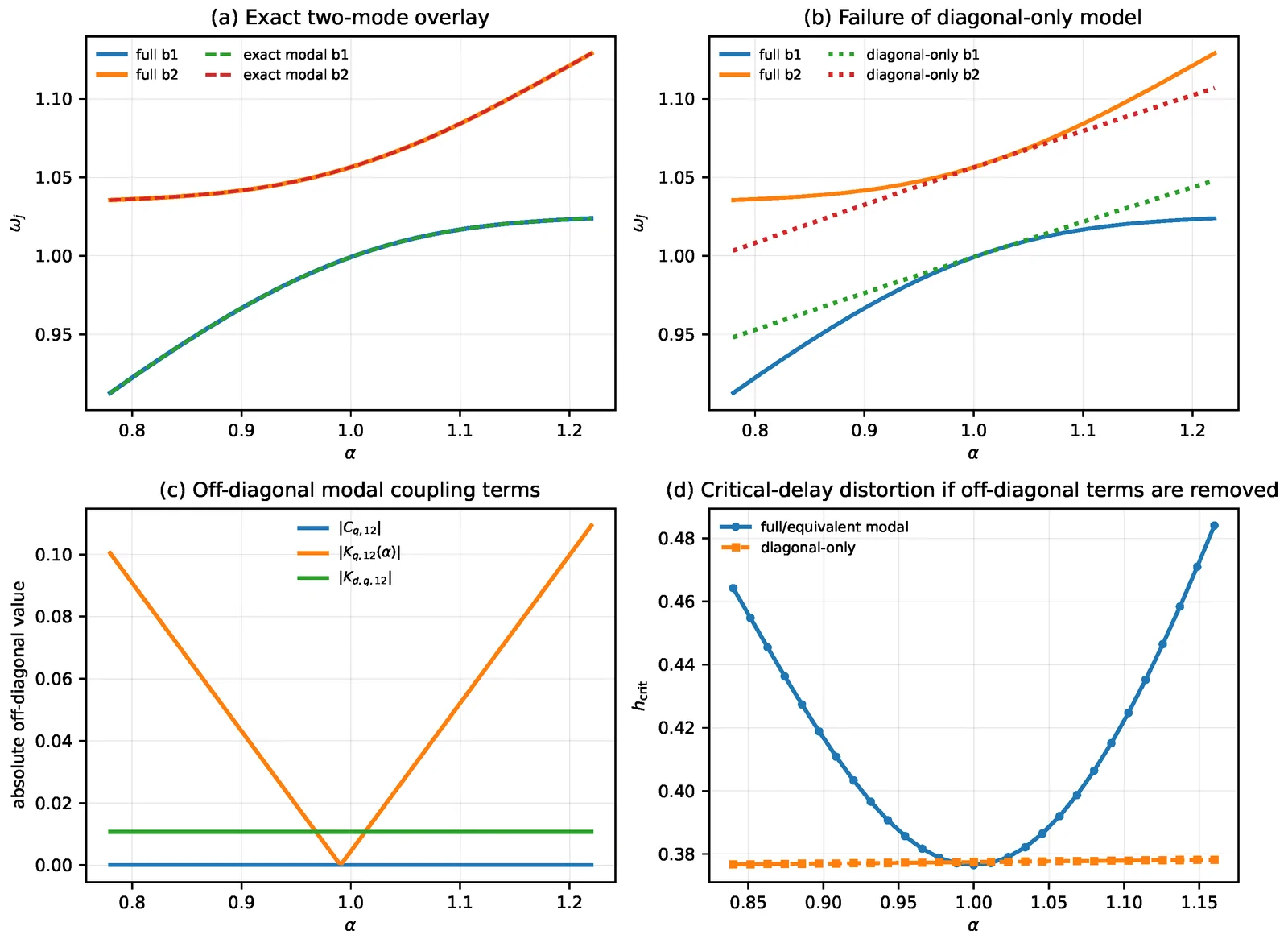
Fixed-reference modal-coordinate representation and the role of off-diagonal modal coupling. The two mass-normalized reference modal vectors define a nonsingular coordinate transformation of the two physical generalized coordinates; this transformation does not constitute a modal diagonalization of the infinite-dimensional DDE. Panels (**a**,**b**) compare the physical-coordinate characteristic roots, the algebraically equivalent fixed-reference modal-coordinate formulation, and a deliberately diagonalized diagnostic approximation. Panel (**c**) displays the off-diagonal delayed, damped, and detuned modal terms retained in the reference coordinates. Panel (**d**) shows that artificially deleting these terms distorts the tracked critical-delay boundary, demonstrating their role in branch interaction near veering.

Figure 6 verifies that the fixed-reference modal-coordinate formulation reproduces the physical-coordinate characteristic roots to round-off accuracy, as required by the nonsingular coordinate transformation. This agreement should not be interpreted as evidence that the DDE has been diagonalized into two independent modes. In contrast, the deliberately diagonal-only diagnostic approximation visibly distorts both the root branches and the critical-delay curve. The comparison therefore shows that the off-diagonal delayed, damped, and detuned terms retained in the reference coordinates are essential for representing branch interaction near veering.

The time-domain branch-energy diagnostics use the same coordinates. With $q=\Phi_{*}^{T}My$ and $\dot{q}=\Phi_{*}^{T}M\dot{y}$, we define

(51) $$E_{j}\left(t\right)=\frac{1}{2}{\dot{q}}_{j}^{2}\left(t\right)+\frac{1}{2}\omega_{j_{*}}^{2}q_{j}^{2}\left(t\right),\;R_{j}\left(t\right)=\frac{E_{j}\left(t\right)}{E_{1}\left(t\right)+E_{2}\left(t\right)+\varepsilon_{E}},\;j=1,2.$$

These $E_{j}$ are reference-modal energy indicators. They are not conserved energies of the damped delayed nonlinear stochastic system. To quantify the relative participation of the two reference-modal branches, we further define the time-integrated normalized energy fractions

(52) $${\bar{\eta }}_{j}=\frac{\int_{T_{\mathrm{warm}}}^{T}E_{j}\left(t\right)\;dt}{\int_{T_{\mathrm{warm}}}^{T}\left[E_{1}\left(t\right)+E_{2}\left(t\right)\right]dt},\;j=1,2,$$

so that

(53) $${\bar{\eta }}_{1}+{\bar{\eta }}_{2}=1.$$

A complementary branch-energy ratio is defined as

(54) $$R_{21}=\frac{\int_{T_{\mathrm{warm}}}^{T}E_{2}\left(t\right)\;dt}{\int_{T_{\mathrm{warm}}}^{T}E_{1}\left(t\right)\;dt}.$$

These quantities are used only as reference-modal energy indicators for comparing branch participation. Since the fixed-reference coordinate transformation does not diagonalize the complete delayed system, $E_{1}$ and $E_{2}$ should not be interpreted as an exact additive decomposition of the total energy of the infinite-dimensional DDE.

### 3.3. Weakly Nonlinear Delayed Veering

The delayed cubic term acts through the same relative coordinate as the linear delayed force. In modal coordinates, with $r=\Phi_{*}^{T}b$,

(55) $$z_{h}=b^{T}y\left(t-h_{c}\right)=r^{T}q\left(t-h_{c}\right),\;\kappa_{3}rz_{h}^{3}=\kappa_{3}r{\left[r^{T}q\left(t-h_{c}\right)\right]}^{3}.$$

The first-harmonic backbone prescribes

(56) $$z_{h}=A_{z}\cos\Omega \left(t-h_{c}\right).$$

Since $\cos^{3}\theta =\left(3/4\right)\cos\theta +\left(1/4\right)\cos3\theta$, we obtain

(57) $$z_{h}^{3}\approx \frac{3}{4}A_{z}^{2}z_{h}.$$

Thus, the delayed effective stiffness is

(58) $$K_{d,\mathrm{eff}}\left(A_{z}\right)=k_{\mathrm{eff}}\left(A_{z}\right)K_{c},\;k_{\mathrm{eff}}\left(A_{z}\right)=k_{d}\tau_{0}+\frac{3}{4}\kappa_{3}A_{z}^{2}.$$

**Lemma 2** 
(First-harmonic effective delayed stiffness)**.** *Under the first-harmonic ansatz $z_{h}=A_{z}\cos\Omega \left(t-h_{c}\right)$, the cubic delayed physical force satisfies*

$$\kappa_{3}bz_{h}^{3}=\frac{3}{4}\kappa_{3}A_{z}^{2}bz_{h}+\frac{1}{4}\kappa_{3}A_{z}^{3}b\cos3\Omega \left(t-h_{c}\right).$$

*At the backbone level, retaining only the fundamental component is equivalent to replacing $k_{d}\tau_{0}K_{c}$ with $K_{d,\mathrm{eff}}\left(A_{z}\right)=k_{\mathrm{eff}}\left(A_{z}\right)K_{c}$, with $k_{\mathrm{eff}}$ given by Equation (58).*

**Proof.**  The identity is obtained obviously, since $\cos^{3}\theta =\left(3/4\right)\cos\theta +\left(1/4\right)\cos3\theta$ and $bz_{h}=K_{c}y\left(t-h_{c}\right)$. Dropping the third harmonic leaves a delayed force proportional to $K_{c}y\left(t-h_{c}\right)$, which adds the term $\left(3/4\right)\kappa_{3}A_{z}^{2}K_{c}$ to the delayed stiffness. □

To quantify the amplitude range over which the first-harmonic approximation remains suitable for quantitative interpretation, we explicitly evaluate the harmonic component discarded in Equation (58). Under the ansatz

$$z_{h}=A_{z}\cos\Omega \left(t-h_{c}\right),$$

the delayed relative-coordinate force can be written as

(59) $$F_{d}=\left(k_{d}\tau_{0}+\frac{3}{4}\kappa_{3}A_{z}^{2}\right)A_{z}\cos\Omega \left(t-h_{c}\right)+\frac{1}{4}\kappa_{3}A_{z}^{3}\cos3\Omega \left(t-h_{c}\right).$$

Accordingly, the amplitudes of the retained fundamental component and the neglected third harmonic are

(60) $$F_{1}=\left(k_{d}\tau_{0}+\frac{3}{4}\kappa_{3}A_{z}^{2}\right)A_{z},\;F_{3}=\frac{1}{4}\kappa_{3}A_{z}^{3}.$$

We therefore define the neglected-harmonic ratio

(61) $$\varepsilon_{3}\left(A_{z}\right)=\frac{F_{3}}{F_{1}}=\frac{\kappa_{3}A_{z}^{2}/4}{k_{d}\tau_{0}+3\kappa_{3}A_{z}^{2}/4}$$

as an explicit indicator of the truncation error associated with the first-harmonic representation. The corresponding cycle-RMS relative force error is

(62) $$\varepsilon_{\mathrm{RMS}}\left(A_{z}\right)=\frac{F_{3}}{\sqrt{F_{1}^{2}+F_{3}^{2}}},$$

which provides a complementary waveform-level measure of the neglected contribution.

For the parameters used in this study, $k_{d}=0.12$, $\tau_{0}=1.0$, and $\kappa_{3}=0.20$, the neglected-harmonic ratio is approximately 3.37% at $A_{z}=0.30$, 9.15% at $A_{z}=0.55$, 10.34% at $A_{z}=0.60$, and 20.07% at $A_{z}=1.10$. Using a 10% neglected-harmonic level as an operational criterion for quantitative interpretation gives

$$A_{z}≃0.586.$$

To retain a conservative weakly nonlinear range, the quantitative first-harmonic spectral results reported below are therefore restricted to

(63) $$0\leq A_{z}\leq 0.55.$$

The 10% level is used here as a practical truncation-error criterion rather than as a rigorous nonlinear-stability threshold.

The criterion in Equation (63) quantifies only the magnitude of the neglected third harmonic. It does not exclude higher-harmonic responses, subharmonic solutions, secondary bifurcations, or other strongly nonlinear dynamics. Therefore, the following results are interpreted as a first-harmonic amplitude-dependent effective-stiffness backbone rather than as a complete nonlinear DDE bifurcation diagram.

The corresponding backbone roots solve

(64) $$\det\{s^{2}M+sC+K_{g}\left(\alpha \right)+K_{d,\mathrm{eff}}\left(A_{z}\right)e^{-sh_{c}}\}=0.$$

Figure 8 records the hardening of the delayed relative-coordinate channel produced by the positive cubic term under the first-harmonic approximation. Within the revised quantitative weakly nonlinear range, the representative backbone curves are shown for $A_{z}=0$, 0.30, and 0.55. Over this range, $k_{\mathrm{eff}}$ increases from approximately 0.1200 to 0.1654. At the representative delay $h_{c}=0.30$, the veering center $\alpha_{\mathrm{cov}}$ shifts from approximately 1.013 to 1.025, while the minimum frequency gap $g_{\omega }$ increases from approximately 0.0571 to 0.0928. The backbone frequency branches therefore move upward and the veering gap broadens as the delayed cubic stiffness strengthens the effective coupling. Panel (d) further shows that the critical-delay boundary shifts downward with increasing amplitude over the plotted detuning range, indicating that the amplitude-dependent delayed coupling also modifies the delay-stability margin. These curves should be interpreted as a first-harmonic amplitude-dependent effective-stiffness backbone rather than as a complete nonlinear DDE bifurcation diagram.

**Figure 7:**
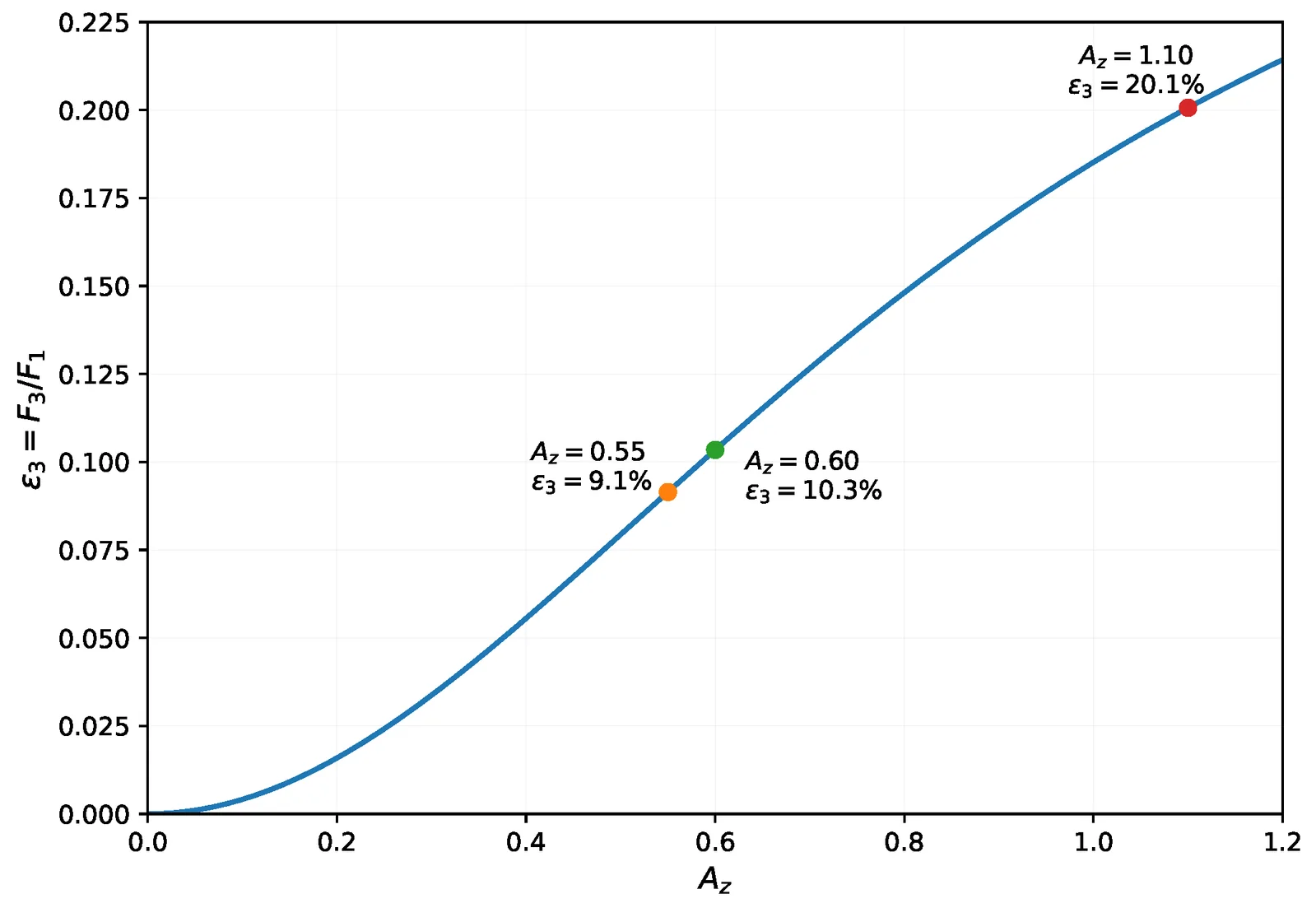
Amplitude dependence of the neglected third-harmonic ratio $\varepsilon_{3}=F_{3}/F_{1}$ for the delayed cubic coupling. The discarded harmonic contribution increases monotonically with $A_{z}$. For the present parameters, $\varepsilon_{3}=9.15\%$ at $A_{z}=0.55$, while the 10% level is reached at $A_{z}≃0.586$. The larger-amplitude values are shown only to illustrate the progressive deterioration of the first-harmonic approximation and are not used for the quantitative backbone results in the revised analysis.

**Figure 8:**
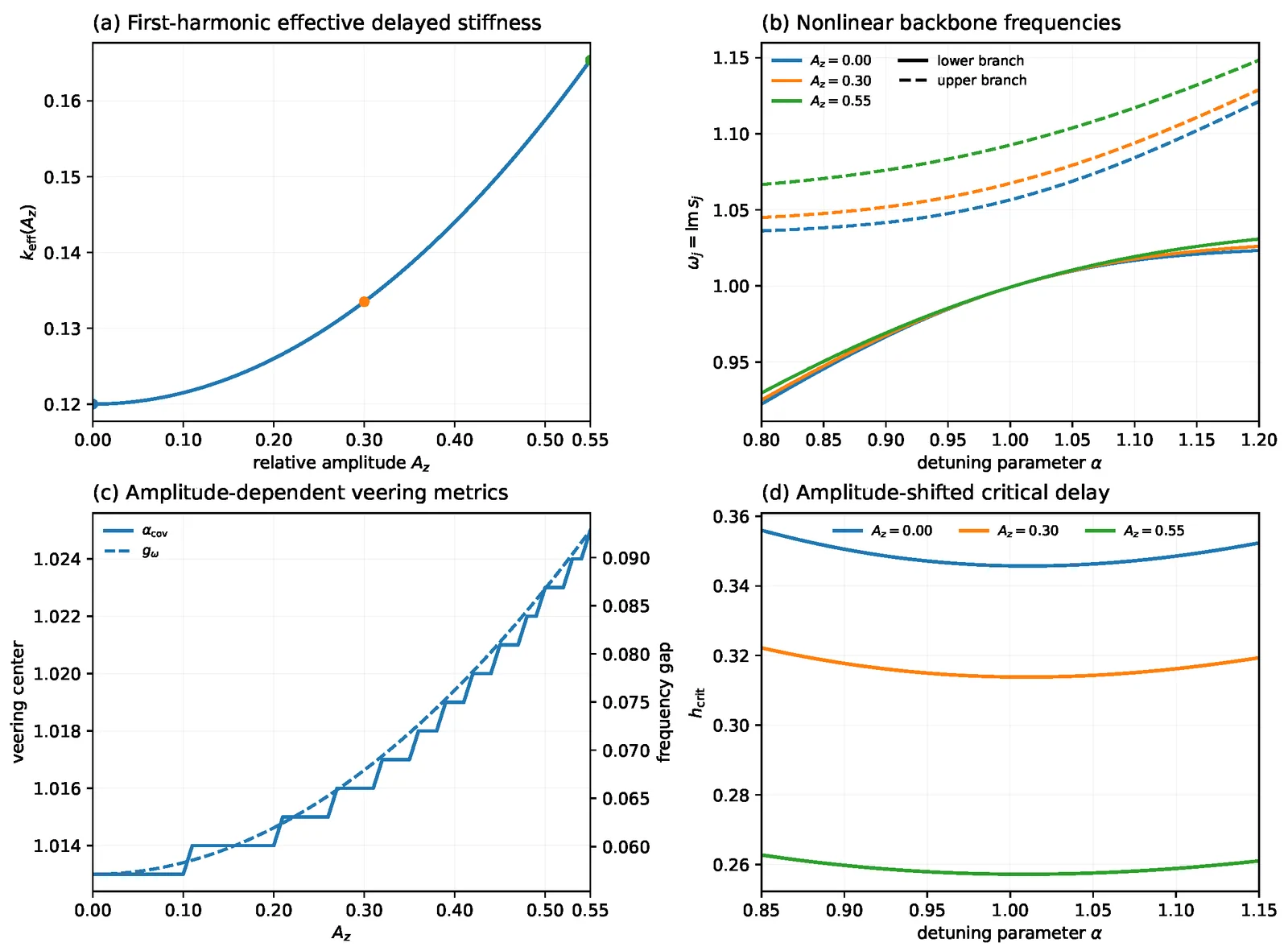
Weakly nonlinear first-harmonic backbone induced by the delayed cubic relative-coordinate coupling. Under the first-harmonic approximation $z_{h}^{3}\approx \left(3/4\right)A_{z}^{2}z_{h}$, the delayed effective stiffness becomes $k_{\mathrm{eff}}\left(A_{z}\right)=k_{d}\tau_{0}+\left(3/4\right)\kappa_{3}A_{z}^{2}$. The figure shows how this amplitude-parametrized stiffness shifts the veering center, enlarges the minimum frequency gap, and modifies the tracked critical-delay boundary. The results should be read as a first-harmonic effective-stiffness backbone, not as a complete nonlinear DDE bifurcation diagram. The orange and green markers in panel (**a**) indicate the representative amplitudes $A_{z}=0.30$ and $A_{z}=0.55$, respectively, which are also used for the comparisons in panels (**b**) and (**d**).

### 3.4. Frozen Stochastic Spectral Clouds

For the frozen stochastic spectral calculation, the positive multiplier is sampled as

(65) $$\gamma_{\sigma }=\exp\left(\sigma \xi -\frac{\sigma^{2}}{2}\right),\;\xi ∼N\left(0,1\right).$$

This is a lognormal random variable with $\gamma_{\sigma }>0$ and $E\gamma_{\sigma }=1$.

**Lemma 3** 
(Frozen lognormal coupling realization)**.** *For every σ≥0, $\gamma_{\sigma }=\exp\left(\sigma \xi -\sigma^{2}/2\right)$, ξ∼N(0,1) is positive almost surely and has unit mean. Therefore, the frozen stochastic spectral problem preserves the nominal delayed coupling level in expectation and avoids negative delayed stiffness realizations.*

**Proof.**  The first assertion follows from positivity of the exponential. The moment-generating function of ξ gives $Ee^{\sigma \xi }=e^{\sigma^{2}/2}$, and hence $E\gamma_{\sigma }=e^{-\sigma^{2}/2}e^{\sigma^{2}/2}=1$. □

The frozen multiplier is also directly connected to the time-dependent stochastic formulation introduced later. For the stationary Ornstein–Uhlenbeck process used in the time-domain model, η(t)∼N(0,1) at any fixed time. Hence,

$$\gamma \left(t\right)=\exp\left(\sigma \eta \left(t\right)-\frac{\sigma^{2}}{2}\right)$$

has the same one-time marginal distribution as $\gamma_{\sigma }$. The two stochastic formulations therefore use the same positive unit-mean instantaneous coupling law. Their distinction lies in temporal structure: $\gamma_{\sigma }$ is held fixed within each spectral realization, whereas γ(t) evolves along a trajectory and retains temporal correlation.

Combining it with the first-harmonic backbone, we have

(66) $$K_{d,\mathrm{eff}}\left(\gamma_{\sigma },A_{z}\right)=\left(k_{d}\tau_{0}\gamma_{\sigma }+\frac{3}{4}\kappa_{3}A_{z}^{2}\right)K_{c}.$$

For each realization, the two veering-related roots are recomputed from

(67) $$\det\{s^{2}M+sC+K_{g}\left(\alpha \right)+K_{d,\mathrm{eff}}\left(\gamma_{\sigma },A_{z}\right)e^{-sh_{c}}\}=0.$$

Thus, the spectral cloud is not produced by adding artificial scatter to deterministic roots.

The random veering center is

(68) $$\alpha_{\mathrm{cov}}\left(\gamma_{\sigma }\right)=\underset{\alpha \in I_{\alpha }}{\arg\min}\left[\omega_{2}\left(\alpha ;\gamma_{\sigma },A_{z},h_{c}\right)-\omega_{1}\left(\alpha ;\gamma_{\sigma },A_{z},h_{c}\right)\right],$$

and the spectral exchange probability is

(69) $$P_{\mathrm{exch}}\left(\alpha ,\sigma \right)=\mathrm{Pr}\;\left[\mathrm{sign}\left(\alpha -\alpha_{\mathrm{cov}}\left(\gamma_{\sigma }\right)\right)\neq \mathrm{sign}\left(\alpha -\alpha_{\mathrm{cov}}^{\left(0\right)}\right)\right].$$

This is a spectral mode-assignment probability. For each frozen sample, we also compute

(70) mtr(n)(σ)=maxj=1,2Resj(αcloud,γσ(n),Az,hc),

and report the empirical quantiles and $\mathrm{Pr}(m_{\mathrm{tr}}>0)$. This probability is a tracked-root positive-growth probability, not a full-spectrum stochastic instability probability.

**Figure 9:**
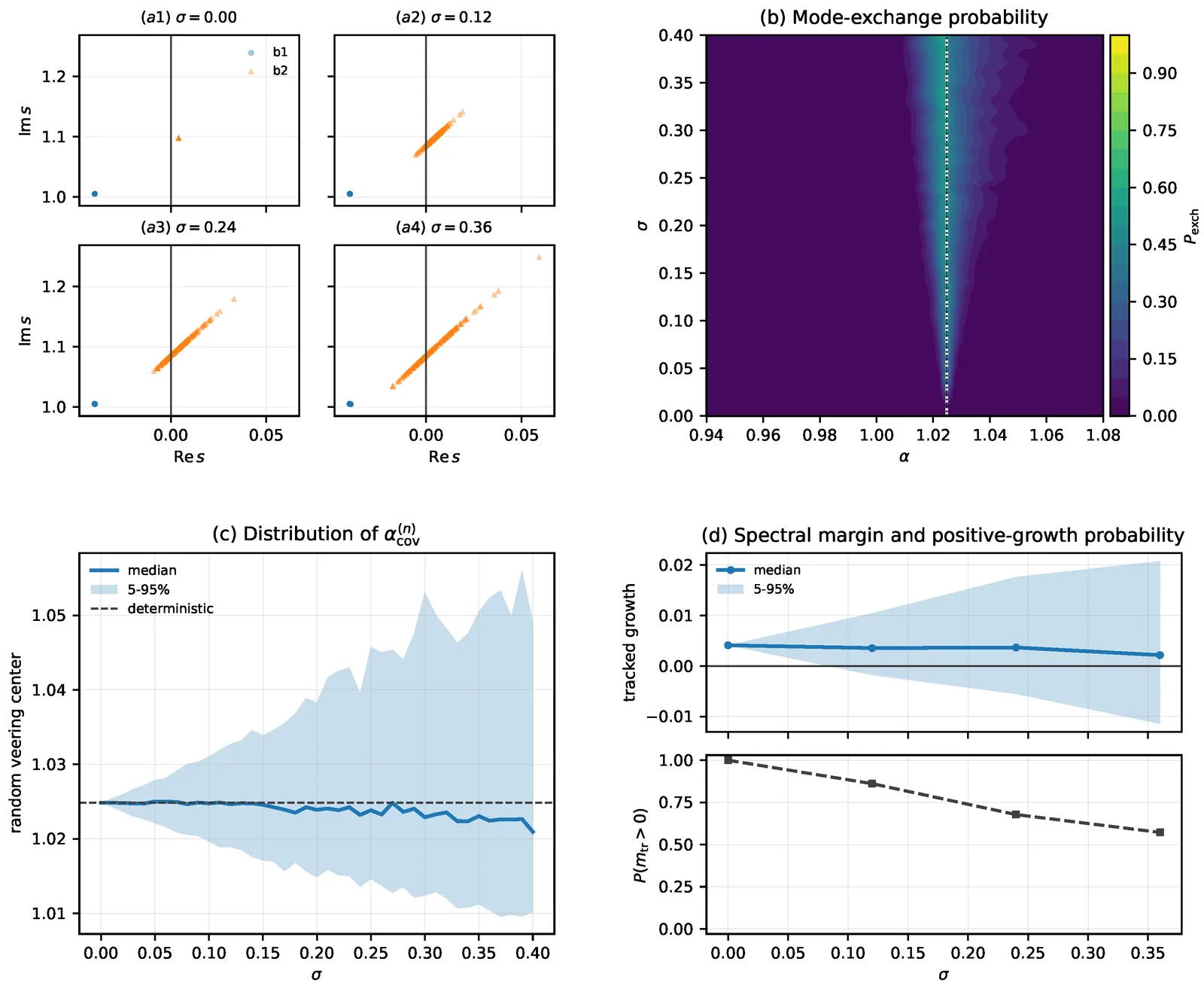
Frozen stochastic spectral uncertainty generated by positive multiplicative coupling modulation. Each frozen realization uses the lognormal multiplier $\gamma_{\sigma }=\exp\left(\sigma \xi -\sigma^{2}/2\right)$ and solves the corresponding characteristic equation for the two veering-related roots. The panels report the spectral cloud, the branch-exchange probability $P_{\mathrm{exch}}\left(\alpha ,\sigma \right)$, the distribution of the random veering center $\alpha_{\mathrm{cov}}^{\left(n\right)}$, and the tracked positive-growth probability $P(m_{\mathrm{tr}}>0)$. The positive-growth probability is branch-tracked and is interpreted as a tracked spectral-margin diagnostic, not as a complete full-spectrum stochastic stability probability. In panel (**b**), the vertical dashed line marks the deterministic veering center $\alpha_{\mathrm{cov}}^{\left(0\right)}$, while the overlaid contour lines indicate constant levels of $P_{\mathrm{exch}}$.

Figure 9 gives the frozen-parameter stochastic counterpart: positive multiplicative uncertainty broadens the complex-root distribution and randomizes the veering center. At σ=0.36, the 5–95% interval of $\alpha_{\mathrm{cov}}^{\left(n\right)}$ is approximately [1.0104,1.0524] around the deterministic value 1.0248 for the nonlinear operating amplitude. The tracked growth-rate statistics at the same noise level have a 5–95% interval of approximately $\left[-1.14\times 10^{-2},2.07\times 10^{-2}\right]$ and $\mathrm{Pr}(m_{\mathrm{tr}}>0)\approx 0.572$. These frozen-sample quantities characterize the static sensitivity of the local veering spectrum to uncertainty in the delayed coupling level. The subsequent OU-driven simulations address a different, trajectory-level question and are used only to examine the dynamic counterpart of this spectral broadening.

**Numerical Finding 2**  (Frozen stochastic spectral broadening)**.** *Within the frozen random-parameter calculation, increasing σ broadens both the complex-root cloud and the distribution of $\alpha_{\mathrm{cov}}^{\left(n\right)}$. The reported $\mathrm{Pr}(m_{\mathrm{tr}}>0)$ is a tracked-root positive-growth probability for the two veering roots.*

### 3.5. Joint Delay–Nonlinearity–Stochastic Interaction

The preceding results characterize the effects of coupling delay, nonlinear amplitude, and stochastic modulation in a sequential manner. To further determine whether their coexistence produces a non-additive interaction, we jointly vary the nonlinear amplitude $A_{z}$ and the stochastic modulation intensity σ at three representative coupling delays, $h_{c}=0.20$, 0.30, and 0.40. The amplitude range is restricted to the weakly nonlinear regime identified by the first-harmonic validity analysis.

For each frozen realization, the sample-dependent veering center is determined from

(71) $$\alpha_{\mathrm{cov}}^{\left(n\right)}=\underset{\alpha \in I_{\alpha }}{\arg\min}\left[\omega_{2}\left(\alpha ;\gamma_{\sigma }^{\left(n\right)},A_{z},h_{c}\right)-\omega_{1}\left(\alpha ;\gamma_{\sigma }^{\left(n\right)},A_{z},h_{c}\right)\right].$$

The corresponding tracked growth indicator at the veering core is defined as

(72) mcore(n)=maxj=1,2Resjαcov(n);γσ(n),Az,hc,

and

(73) $$P_{+}\left(h_{c},A_{z},\sigma \right)=\mathrm{Pr}\left(m_{\mathrm{core}}>0\right)$$

measures the fraction of frozen realizations for which the two tracked veering-related roots enter the positive-growth side of the spectral boundary. As in the preceding frozen-spectrum analysis, $P_{+}$ is used only as a tracked spectral-margin diagnostic and should not be interpreted as a full-spectrum stochastic instability probability.

To distinguish genuine three-factor coupling from a simple superposition of lower-order effects, we introduce the three-way interaction residual:

(74) $$\begin{array}{cc} I_{3}\left[Q\right]\left(h_{c},A_{z},\sigma \right)= & Q\left(h_{c},A_{z},\sigma \right)-Q\left(h_{c},A_{z},0\right)-Q\left(h_{c},0,\sigma \right)-Q\left(0,A_{z},\sigma \right)\\ \; & +Q\left(h_{c},0,0\right)+Q\left(0,A_{z},0\right)+Q\left(0,0,\sigma \right)-Q\left(0,0,0\right), \end{array}$$

where *Q* denotes a selected spectral statistic. For the risk-oriented quantity $P_{+}$, $I_{3}\left[P_{+}\right]>0$ indicates that the combined action increases the positive-growth probability beyond the corresponding lower-order contributions, whereas $I_{3}\left[P_{+}\right]<0$ indicates partial compensation among the three effects.

**Figure 10:**
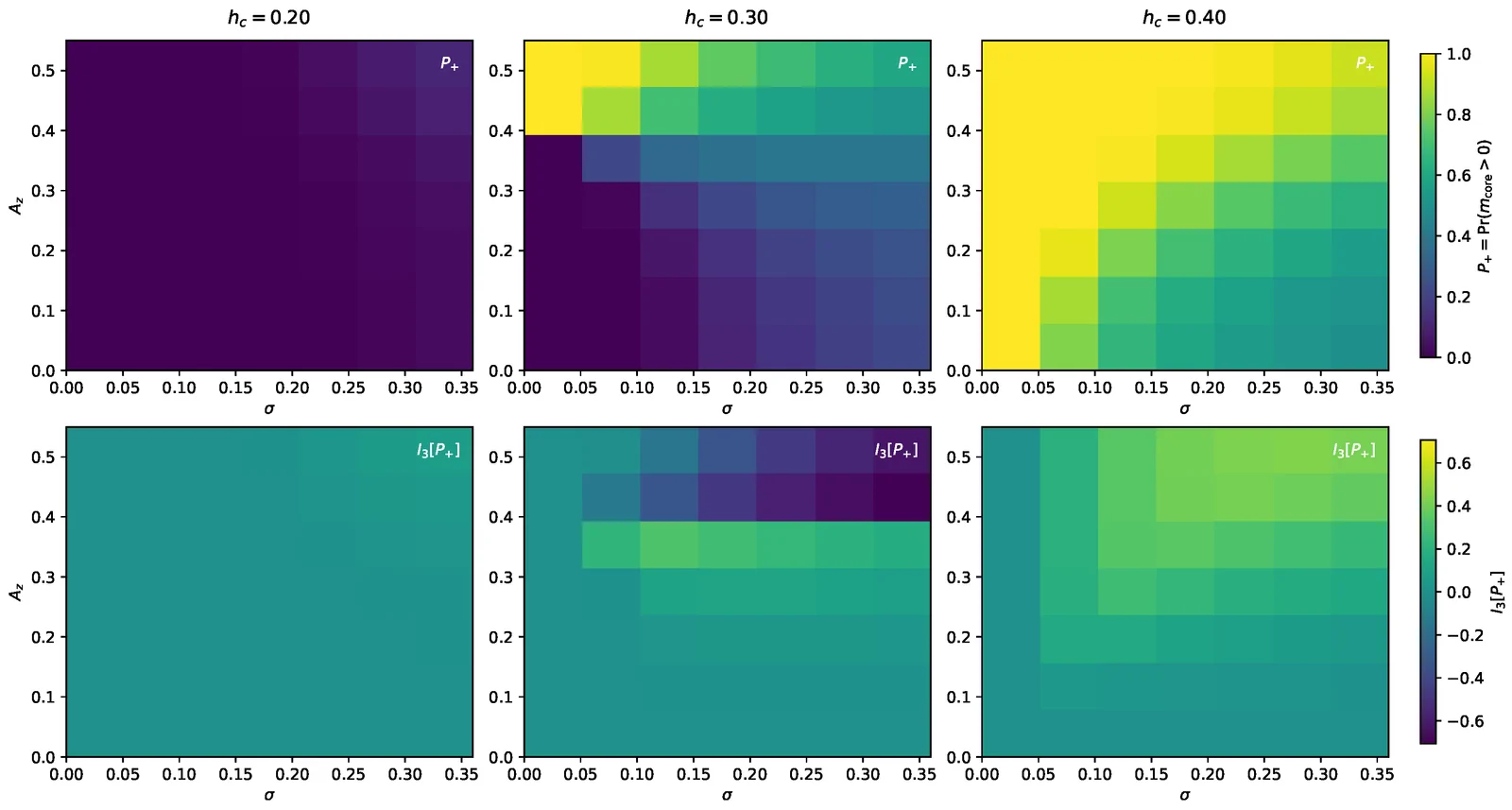
Joint influence of coupling delay, nonlinear amplitude, and stochastic modulation on the tracked spectral-margin probability. The three columns correspond to $h_{c}=0.20$, 0.30, and 0.40, respectively. The upper row shows the positive-growth probability $P_{+}=\mathrm{Pr}\left(m_{\mathrm{core}}>0\right)$, where $m_{\mathrm{core}}$ is the maximum real part of the two tracked veering-related roots evaluated at the sample-dependent veering center. The lower row shows the corresponding three-way interaction residual $I_{3}\left[P_{+}\right]$. Positive values of $I_{3}\left[P_{+}\right]$ indicate non-additive synergistic amplification of the positive-growth probability, whereas negative values indicate partial compensation or antagonistic interaction.

Figure 10 shows that the interaction among delay, nonlinearity, and stochastic modulation is strongly dependent on the location of the underlying deterministic operating point relative to the spectral stability boundary. When the deterministic system remains on the stable side or approaches the boundary, increasing $A_{z}$ reduces the spectral margin and stochastic modulation broadens the root distribution. Their coexistence consequently increases the fraction of realizations entering the positive-growth region, producing a synergistic risk-amplification effect.

A different behavior appears after the deterministic delay–nonlinearity combination has already moved the tracked roots to the positive-growth side. In this regime, stochastic broadening redistributes part of the frozen realizations back toward the negative-growth side, so that the positive-growth probability can decrease with increasing stochastic intensity. The resulting negative interaction residual corresponds to an antagonistic probability response. Thus, whether the joint effect is synergistic or antagonistic is not a fixed property of the three parameters themselves, but depends on the distance and side of the deterministic operating point relative to the spectral stability boundary.

The joint calculations further show that the non-additive interaction is much weaker for mean spectral quantities, including the mean veering location and the mean tracked growth indicator, than for the thresholded probability $P_{+}$. This distinction arises because relatively small changes in the root distribution can produce a large change in probability when the distribution lies close to the boundary $m_{\mathrm{core}}=0$. Therefore, the principal coupled effect of delay, weak nonlinearity, and stochastic modulation is most clearly manifested in the probability of crossing the tracked spectral boundary rather than in a large non-additive displacement of the mean spectrum.

### 3.6. Time-Domain Monte Carlo Evidence

The time-domain simulations use the full nonlinear stochastic DDE:

(75) $$M\ddot{y}\left(t\right)+C\dot{y}\left(t\right)+K_{g}\left(\alpha \right)y\left(t\right)+k_{d}\tau_{0}\gamma \left(t\right)K_{c}y\left(t-h_{c}\right)+\kappa_{3}b{\left[b^{T}y\left(t-h_{c}\right)\right]}^{3}=0,$$

where

(76) $$\gamma \left(t\right)=\exp\left(\sigma \eta \left(t\right)-\frac{\sigma^{2}}{2}\right),\;d\eta \left(t\right)=-\theta \eta \left(t\right)dt+\sqrt{2\theta }\;dW_{t}.$$

The OU process is updated exactly over one time step, and the second-order delayed equation is integrated using the fixed time step reported in Table 1. Although γ(t) has the same stationary one-time lognormal marginal distribution as the frozen multiplier $\gamma_{\sigma }$, the OU formulation retains temporal dependence through

$$E\left[\eta \left(t\right)\eta \left(s\right)\right]=e^{-\theta |t-s|}.$$

Consequently, the response of the full nonlinear DDE depends not only on the instantaneous coupling realization but also on the temporal sequence of fluctuations, the delayed nonlinear dynamics, the initial history, and the finite observation interval. The OU calculation is therefore not a time-dependent implementation of the frozen characteristic-root calculation; it represents a distinct pathwise stochastic problem with the same instantaneous coupling marginal.

The pathwise energy balance explains why delay can produce branch-energy exchange. Define

(77) $$E\left(t\right)=\frac{1}{2}{\dot{y}}^{T}\left(t\right)M\dot{y}\left(t\right)+\frac{1}{2}y^{T}\left(t\right)K_{g}\left(\alpha \right)y\left(t\right).$$

Multiplying the governing equation by ${\dot{y}}^{T}\left(t\right)$, we get

(78) $$\dot{E}\left(t\right)=-{\dot{y}}^{T}C\dot{y}-k_{d}\tau_{0}\gamma \left(t\right)\left[b^{T}\dot{y}\left(t\right)\right]\left[b^{T}y\left(t-h_{c}\right)\right]-\kappa_{3}\left[b^{T}\dot{y}\left(t\right)\right]{\left[b^{T}y\left(t-h_{c}\right)\right]}^{3}.$$

The damping term is dissipative, while the delayed linear and cubic work rates are sign-indefinite, because the current relative velocity is paired with delayed relative displacement. Therefore, stochastic modulation changes the intensity of a nonconservative delayed energy-transfer pathway.

For quantitative comparison of the energy-flow channels, the delayed power is separated into its linear and cubic components. With

$$z\left(t\right)=b^{T}y\left(t\right),\;z_{h}=z\left(t-h_{c}\right),$$

we define

(79) $$\begin{array}{cc} P_{g}\left(t\right) & =-c_{g}{∥\dot{y}\left(t\right)∥}^{2},\\ P_{c}\left(t\right) & =-c_{c}{\dot{z}}^{2}\left(t\right),\\ P_{d,L}\left(t\right) & =-k_{d}\tau_{0}\gamma \left(t\right)z_{h}\dot{z}\left(t\right),\\ P_{d,\mathrm{NL}}\left(t\right) & =-\kappa_{3}z_{h}^{3}\dot{z}\left(t\right). \end{array}$$

Because the delayed-power terms are sign-indefinite, their signed time averages alone may underestimate their dynamical contribution through cancellation between positive and negative work. We therefore evaluate both the signed mean power

(80) $${\bar{P}}_{j}=\frac{1}{T-T_{\mathrm{warm}}}\int_{T_{\mathrm{warm}}}^{T}P_{j}\left(t\right)\;dt,$$

and the normalized absolute-work fraction

(81) $$\chi_{j}=\frac{\int_{T_{\mathrm{warm}}}^{T}\left|P_{j}\left(t\right)\right|\;dt}{\sum_{k}\int_{T_{\mathrm{warm}}}^{T}\left|P_{k}\left(t\right)\right|\;dt}.$$

The former identifies the net direction of energy transfer, whereas the latter measures the relative activity of each energy-flow channel.

**Proposition 4** 
(Pathwise energy identity)**.** *For every differentiable sample path of the OU-driven system, the mechanical energy E(t) satisfies Equation (78). Hence, the only sign-definite in this identity is the viscous damping term. The delayed linear and cubic relative-coordinate work rates are sign-indefinite.*

**Proof.**  Premultiplying the stochastic delayed equation by ${\dot{y}}^{T}\left(t\right)$ gives ${\dot{y}}^{T}M\ddot{y}=d\left({\dot{y}}^{T}M\dot{y}/2\right)/dt$ and ${\dot{y}}^{T}K_{g}\left(\alpha \right)y=d\left(y^{T}K_{g}\left(\alpha \right)y/2\right)/dt$. Moving the damping and delayed force terms to the right-hand side yields Equation (78). The sign-definiteness statement is true, since *C* is positive semidefinite, whereas the products $\left[b^{T}\dot{y}\left(t\right)\right]\left[b^{T}y\left(t-h_{c}\right)\right]$ and $\left[b^{T}\dot{y}\left(t\right)\right]{\left[b^{T}y\left(t-h_{c}\right)\right]}^{3}$ can change sign along a trajectory. □

The delayed-work statistic used to connect the probability map with Equation (78) is

(82) $$W_{d}\left(T\right)=-\int_{T_{0}}^{T}k_{d}\tau_{0}\gamma \left(t\right)\left[b^{T}\dot{y}\left(t\right)\right]\left[b^{T}y\left(t-h_{c}\right)\right]\;dt.$$

**Proposition 5** 
(Finite-time residence observable)**.** *For fixed $T_{\mathrm{warm}}$, T, $R_{\mathrm{th}}$, and $\rho_{\mathrm{th}}$, $P_{\mathrm{TD}}\left(\alpha ,\sigma ;\;R_{\mathrm{th}},\rho_{\mathrm{th}}\right)$ is a well-defined probability on the Monte Carlo ensemble generated by Equation (78) and the OU multiplier. It is a finite-time observable depending on the initial history, observation window, and threshold choices, and it should not be identified with an invariant-measure switching probability.*

To make the threshold dependence explicit, define the instantaneous branch-2 energy share as

(83) $$R_{2}\left(t\right)=\frac{E_{2}\left(t\right)}{E_{1}\left(t\right)+E_{2}\left(t\right)},$$

and, for a prescribed instantaneous threshold $R_{\mathrm{th}}$, define the corresponding residence fraction by

(84) $$\rho_{2}\left(R_{\mathrm{th}}\right)=\frac{1}{T-T_{\mathrm{warm}}}\int_{T_{\mathrm{warm}}}^{T}1_{\left\{R_{2}\left(t\right)>R_{\mathrm{th}}\right\}}\;dt.$$

The finite-time residence probability is therefore more explicitly written as

(85) $$P_{\mathrm{TD}}\left(\alpha ,\sigma ;R_{\mathrm{th}},\rho_{\mathrm{th}}\right)=\mathrm{Pr}\left[\rho_{2}\left(R_{\mathrm{th}}\right)>\rho_{\mathrm{th}}\right].$$

Equation (85) emphasizes that $P_{\mathrm{TD}}$ is an operational, threshold-dependent path observable. Accordingly, its numerical value should always be interpreted together with the prescribed thresholds, initial history, warm-up interval, and finite observation window.

The frozen spectral and time-domain probabilities should therefore not be identified. In particular,

$$\mathrm{Pr}(m_{\mathrm{tr}}>0)$$

and the sample-dependent quantity $P_{+}$ introduced in the joint spectral analysis measure the fraction of frozen coupling realizations for which the tracked veering roots lie on the positive-growth side of the spectral boundary. By contrast,

$$P_{\mathrm{TD}}\left(\alpha ,\sigma \right)=\mathrm{Pr}\left(\rho_{2}>\rho_{\mathrm{th}}\right)$$

is a finite-time path probability based on branch-energy residence in the nonlinear OU-driven DDE. Since the latter depends on temporal correlation, initial history, observation time, and residence thresholds, there is no theoretical requirement that these spectral and time-domain probabilities take the same numerical value.

**Figure 11:**
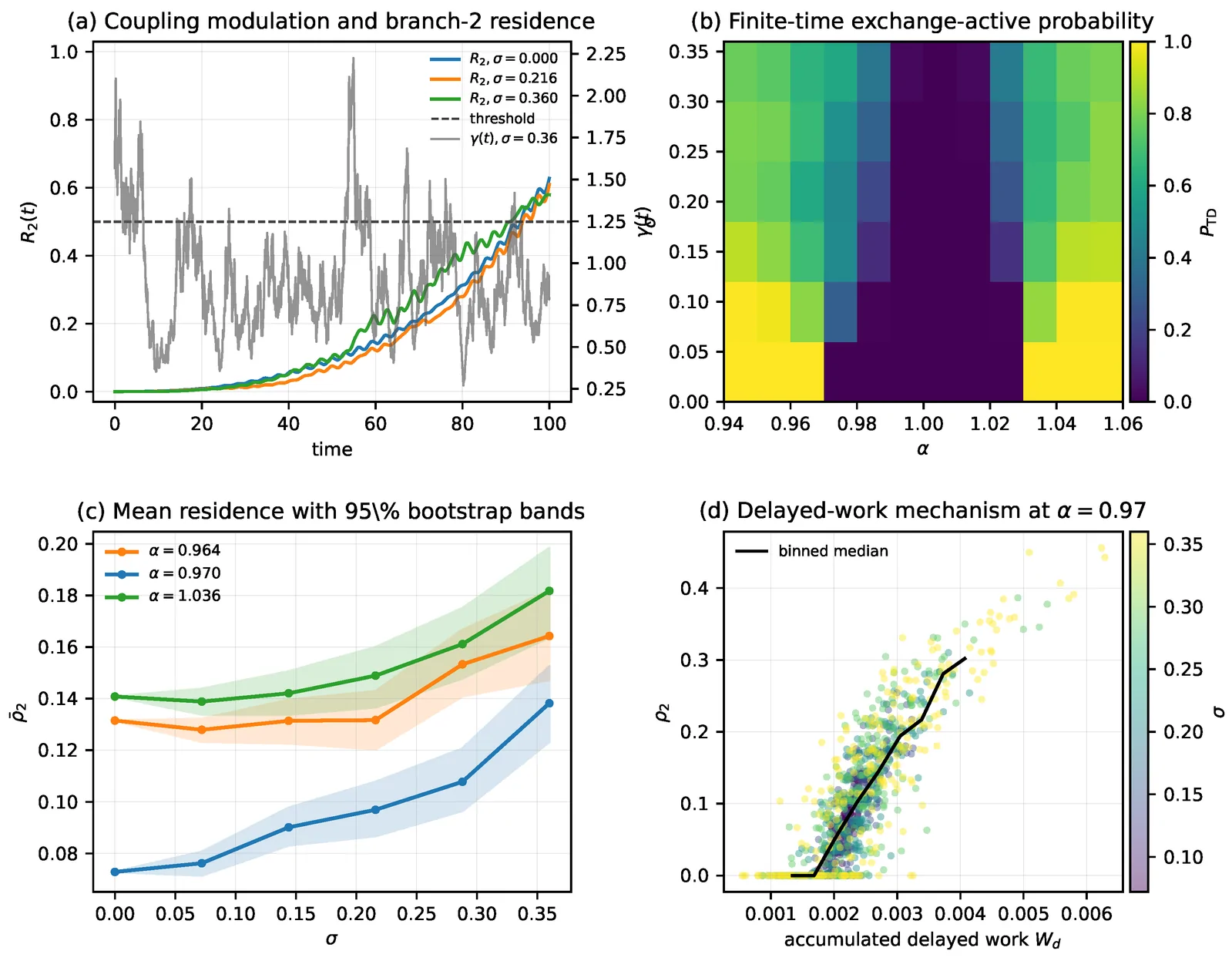
Stochastic time-domain branch residence and delayed-work mechanism. The time-domain simulations use the positive OU-driven multiplier $\gamma \left(t\right)=\exp(\sigma \eta \left(t\right)-\sigma^{2}/2)$ in the delayed relative-coordinate coupling channel, and track the branch-2 energy fraction $R_{2}\left(t\right)$. The residence statistic $\rho_{2}$ and the finite-time event probability $P_{\mathrm{TD}}=P\left(\rho_{2}>\rho_{\mathrm{th}}\right)$ quantify branch-residence activity after the warm-up interval. Panel (**a**) shows representative histories of $R_{2}\left(t\right)$ for σ=0, 0.216, and 0.360, together with the residence threshold and the OU-driven multiplier γ(t) for σ=0.36. Panel (**b**) maps the finite-time event probability $P_{\mathrm{TD}}$ over the (α,σ) plane. Panel (**c**) reports the mean residence fraction together with 95% bootstrap uncertainty bands. Panel (**d**) shows the relation between the accumulated delayed work $W_{d}$ and the residence fraction $\rho_{2}$ at α=0.97, with the points colored by σ and the black curve indicating the binned median. These are finite-time, thresholded residence statistics of free-decay trajectories under stochastic parametric modulation, not stationary switching probabilities.

Figure 11 provides the trajectory-level counterpart of the frozen spectral broadening, rather than a pointwise probabilistic validation of the frozen spectral quantities. The two calculations act through the same positive multiplicative delayed-coupling channel and share the same stationary one-time lognormal marginal law, but only the OU formulation retains temporal correlation and path dependence. The displayed grid is refined to a 12-by-6 scan with α∈[0.94,1.06], including the operating point α=0.97, and σ∈{0,0.072,0.144,0.216,0.288,0.360}. Each grid point uses $N_{\mathrm{MC}}=256$ trajectories. At α=0.97, $P_{\mathrm{TD}}$ changes from 0 at σ=0 to 0.5977 at σ=0.36, with a 95% bootstrap interval [0.5331, 0.6544]. The mean residence fraction increases from approximately 0.0729 to 0.1382, with a 95% bootstrap interval [0.1240, 0.1540] at σ=0.36. Panel (c) reports 95% bootstrap uncertainty bands for ${\bar{\rho }}_{2}$, and the operating-point confidence table is provided in the Supplementary Materials. The response is not treated as a universal noise-enhancement law. Instead, stochastic modulation is interpreted as broadening a finite-time transition region around the veering core. The delayed-work statistics close the mechanism chain by linking the observed residence to the sign-indefinite delayed coupling power. Because $P_{\mathrm{TD}}$ is defined through two operational thresholds, we further examine whether the residence classification changes when these thresholds are varied. A two-dimensional sensitivity scan is performed over

$$0.50\leq R_{\mathrm{th}}\leq 0.99,\;0.10\leq \rho_{\mathrm{th}}\leq 0.95,$$

using 35 uniformly spaced values in each direction. The analysis is repeated for

α=0.8903,0.9303,0.9703,1.0103,1.0503,1.0903,1.1303

and

σ=0,0.12,0.24,0.36.

The purpose of this calculation is not to establish threshold invariance, which is neither expected nor required for $P_{\mathrm{TD}}$, but to identify where the residence classification is saturated and where it is sensitive to the chosen thresholds.

**Figure 12:**
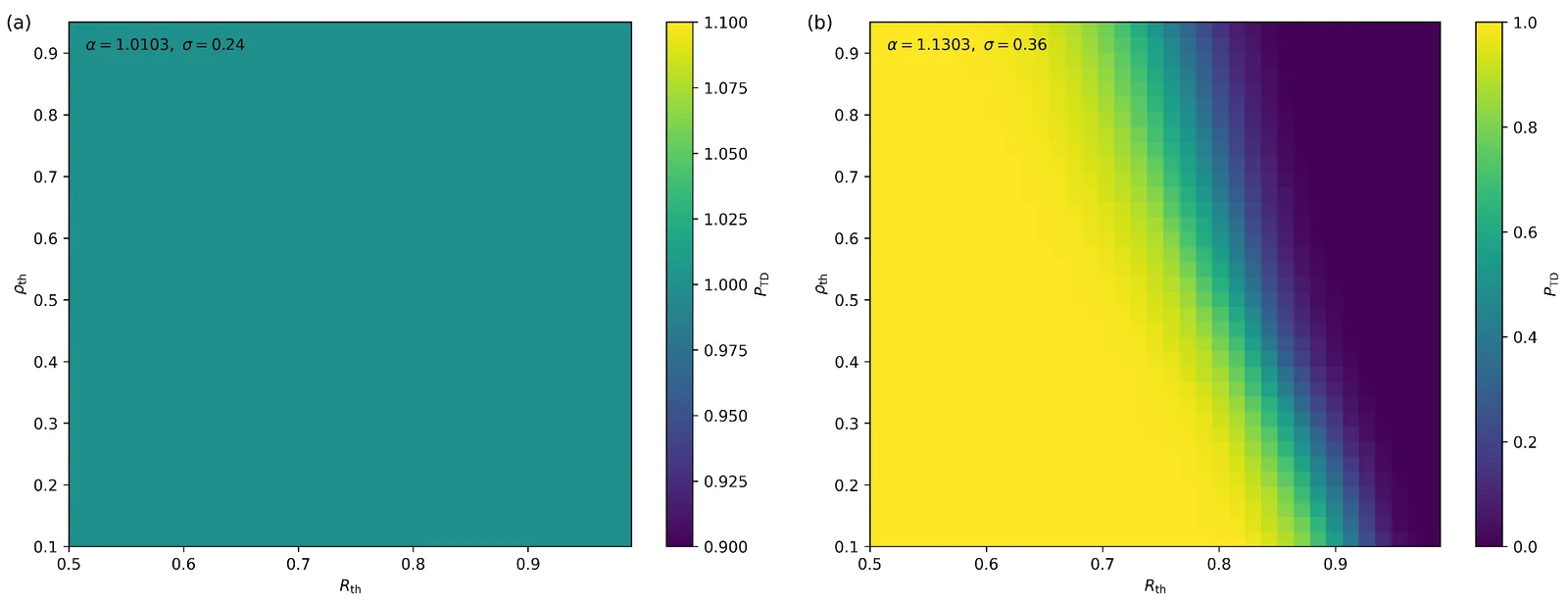
Threshold sensitivity of the finite-time residence probability $P_{\mathrm{TD}}$. (**a**) Saturated residence near the reference veering core (α=1.0103,σ=0.24), where $P_{\mathrm{TD}}=1$ throughout the scanned threshold domain. (**b**) Threshold-sensitive residence away from the core (α=1.1303,σ=0.36), showing a transition from high to low residence probability as $R_{\mathrm{th}}$ and $\rho_{\mathrm{th}}$ are increased. The sensitivity audit uses $N_{\mathrm{MC}}=1000$ trajectories per case, while the remaining time-domain settings are consistent with the baseline OU-driven simulations.

Figure 12 shows that the threshold dependence of $P_{\mathrm{TD}}$ is strongly state dependent. Near the reference veering core,

$$\alpha_{*}=1.0103,$$

the residence observable is saturated. At σ=0.24,

$$P_{\mathrm{TD}}=1$$

throughout the complete scanned threshold domain. Moreover, changing the stochastic intensity from σ=0.12 to 0.36 at the same detuning gives

$$P_{\mathrm{TD}}\left(\sigma =0.36\right)-P_{\mathrm{TD}}\left(\sigma =0.12\right)=0$$

throughout this domain. Hence, sufficiently close to the veering core, the binary residence observable becomes saturated and cannot resolve further changes in stochastic intensity within the tested range.

Away from the veering core, the situation is different. For the representative case

α=1.1303,σ=0.36,

the residence probability spans the full interval

$$0\leq P_{\mathrm{TD}}\leq 1$$

as the two thresholds are varied. Approximately 29.9% of the scanned threshold plane lies in the intermediate range

$$0.05<P_{\mathrm{TD}}<0.95,$$

which forms a clear transition region between high- and low-residence classifications.

These results demonstrate that $P_{\mathrm{TD}}$ should not be interpreted as a threshold-independent system property. Instead, its threshold sensitivity depends on the dynamical location: the observable is effectively threshold insensitive when residence is strongly saturated near the veering core, whereas substantial threshold dependence appears away from the core. Conclusions based on $P_{\mathrm{TD}}$ are therefore restricted to the stated threshold, initial-history, and finite-observation conditions.

Accordingly, the relation between the two stochastic frameworks is mechanistic rather than probabilistic equality: the frozen analysis quantifies static spectral sensitivity to coupling uncertainty, whereas the OU simulation quantifies its finite-time dynamic manifestation.

To quantify the relative branch-energy occupation and the contributions of the individual energy-flow channels, an additional Monte Carlo audit is performed at the representative delay $h_{c}=0.30$. The time-domain parameters are kept consistent with the baseline OU-driven simulations, with Δt=0.02, T=100, and θ=0.40. To reduce the sampling uncertainty of the normalized energy and work fractions, the ensemble size is increased from the baseline value $N_{\mathrm{MC}}=256$ to $N_{\mathrm{MC}}=1000$ trajectories for this quantitative audit.

**Figure 13:**
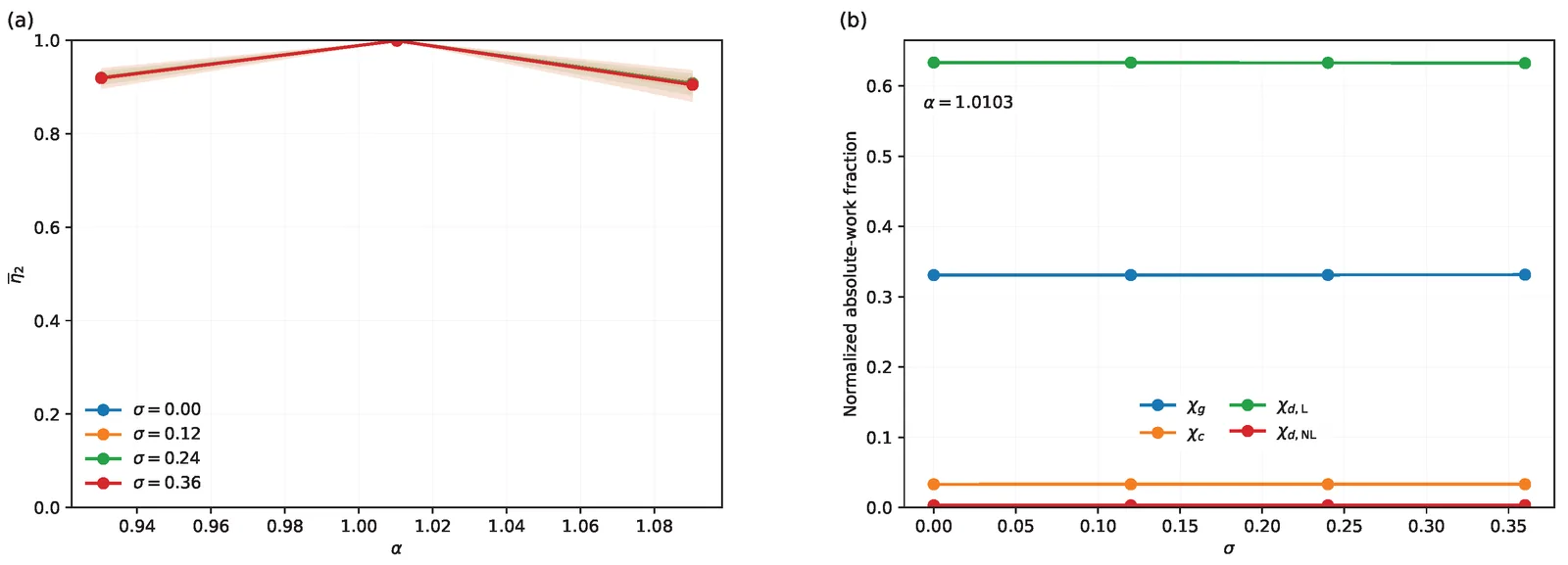
Quantitative energy-contribution analysis for the OU-driven nonlinear delayed system. (**a**) Time-integrated normalized energy fraction of the second reference-modal branch for α=0.9303, 1.0103, and 1.0903 at different stochastic intensities. (**b**) Normalized absolute-work fractions of grounding damping, relative-motion damping, linear delayed coupling, and cubic delayed coupling at the reference veering core $\alpha_{*}=1.0103$. The audit uses $N_{\mathrm{MC}}=1000$ trajectories, while the remaining time-domain parameters are consistent with those of the baseline OU-driven simulations.

Figure 13 provides a quantitative assessment of the branch-energy occupation and pathwise energy-flow channels. For the prescribed initial history and finite-time ensemble, the second reference-modal branch carries

$$0.9049\leq {\bar{\eta }}_{2}\leq 0.9993$$

over the tested combinations

α=0.9303,1.0103,1.0903,σ=0,0.12,0.24,0.36.

Near the reference veering core, $\alpha_{*}=1.0103$, the corresponding branch-energy ratio is approximately

$$1.38\times 10^{3}\leq R_{21}\leq 1.45\times 10^{3},$$

whereas values of approximately 9.9–11.7 are obtained at the two off-core locations. Thus, under the prescribed initial history and finite-time observation conditions, the second reference-modal branch carries the dominant reference-modal energy contribution throughout the examined range.

The absolute-work decomposition provides a separate measure of energy-transfer activity. The linear delayed-coupling channel gives the largest contribution in every tested case,

$$0.5998\leq \chi_{d,L}\leq 0.6333.$$

The grounding-damping contribution is approximately 0.331–0.366, the relative-motion damping contribution is approximately 0.032–0.033, and the cubic delayed term contributes only approximately 0.0022–0.0033. The linear delayed-coupling term is therefore the dominant single pathwise energy-transfer channel within the examined regime.

The signed mean powers further show that the damping channels remain dissipative, whereas the delayed coupling provides a positive net energy-transfer contribution over the finite-time realizations. Moreover, increasing σ from 0 to 0.36 does not change the ordering of the principal energy contributions. This indicates that stochastic modulation changes trajectory-level variability without altering the hierarchy of the dominant energy-flow channels over the tested intensity range.

As a numerical consistency check, the maximum case-averaged relative residual of the reconstructed energy balance is $6.38\times 10^{-5}$, while the maximum 95th-percentile residual is $9.27\times 10^{-5}$.

**Numerical Finding 3**  (Finite-time stochastic branch residence)**.** *For the displayed OU Monte Carlo grid, the event probability $P_{\mathrm{TD}}$ and the distribution of $\rho_{2}$ change with σ in the neighborhood of the veering region. These are finite-time, thresholded, initial-condition-dependent residence statistics. They provide trajectory-level evidence that the frozen spectral broadening has a dynamic counterpart, but they do not constitute a stationary switching law or a stochastic bifurcation theorem.*

### 3.7. Robustness and Synthesis

The Supplementary Material includes checks on time step, sample size, threshold, and first switching for the stochastic time-domain statistics. These checks do not convert the Monte Carlo evidence into a theorem, but they reduce the possibility that Figure 11 is an artifact of a single time step, sample size, or threshold choice. Putting Figure 2, Figure 3, Figure 4, Figure 5, Figure 6, Figure 7, Figure 8, Figure 9, Figure 10 and Figure 11 together, we get the following scoped chain: coupling delay creates a complex-root veering landscape; off-diagonal modal terms carry branch interaction; delayed cubic coupling deforms the veering backbone; positive stochastic coupling modulation broadens the spectral distribution; and time-domain OU simulations show a finite-time residence counterpart governed by sign-indefinite delayed work.

## 4. Discussion

### 4.1. Mechanistic Interpretation

From the preceding lemmas, propositions, and numerical findings, we get a mechanism. In an inertially coupled oscillator, putting a delay in the relative-coordinate coupling first creates a complex-root veering landscape. The coupling delay does not just shift the frequencies. It also changes the rightmost characteristic roots. Hence, it changes the proximity to instability. Then, the cubic coupling warps that same landscape through an amplitude-dependent effective delayed stiffness. Finally, multiplicative stochastic modulation scrambles the effective coupling strength, which leads to spectral clouds and uncertainty in branch exchange. The time-domain Monte Carlo simulations show that these spectral changes have a trajectory-level counterpart in branch-energy residence and first-switching statistics. For vibration applications, an inertia-coupled attachment or internal relative-motion mechanism may exhibit delay-sensitive branch exchange even when the nominal deterministic tuning appears acceptable. Therefore, the results are relevant to frequency-control devices, inertially amplified metamaterials, and vibration absorbers, where sensing, actuation, or internal force transmission introduces an effective coupling lag.

### 4.2. Engineering Implications

The preceding results provide several design implications for vibration systems in which relative-coordinate coupling is affected by delay, weak nonlinearity, and parameter fluctuations. First, the coupling delay should not be treated merely as a small implementation error. The spectral calculations show that varying $h_{c}$ can simultaneously shift the veering center, alter the minimum frequency gap, and change the real parts of the tracked characteristic roots. Consequently, delays associated with sensing, computation, actuation, or internal force transmission should be included explicitly during the tuning of delayed absorbers, inertially coupled attachments, and related frequency-control devices. A design selected solely from the zero-delay model may therefore operate at a different veering location or with a smaller spectral stability margin once the actual coupling lag is introduced.

Second, the delayed cubic stiffness introduces amplitude-dependent tuning. Within the quantified weakly nonlinear range, increasing $A_{z}$ changes the effective delayed stiffness and consequently shifts the veering characteristics. This implies that a tuning condition obtained from infinitesimal-amplitude or purely linear analysis need not remain optimal over the expected operating-amplitude range. For practical parameter selection, the veering location and the associated spectral margin should therefore be checked over the range of response amplitudes expected in operation rather than at a single nominal amplitude.

Third, stochastic coupling modulation mainly broadens the distributions of the veering location and the tracked spectral quantities. Hence, a deterministic parameter set with an acceptable nominal spectral margin may still admit realizations much closer to, or beyond, the positive-growth boundary. This observation suggests that practical designs should retain an uncertainty margin instead of placing the nominal operating point directly adjacent to the spectral stability boundary.

Finally, the joint-parameter analysis shows that delay, nonlinear amplitude, and stochastic modulation should not in general be treated as three independent corrections. Near the spectral stability boundary, their combined influence on the positive-growth probability can be strongly non-additive: stochastic broadening can amplify the risk when the deterministic operating point remains on the stable side, whereas partial compensation can occur after the delay–nonlinearity combination has already shifted the system to the positive-growth side. Robust tuning should therefore be performed jointly over the relevant ranges of $h_{c}$, $A_{z}$, and σ, rather than by optimizing the delay, nonlinear correction, and uncertainty effects separately.

These conclusions are intended as mechanism-level design guidance for delayed relative-motion coupling devices. The dimensional mapping introduced in Section 2 allows the reported nondimensional delay, damping, stiffness, amplitude, and spectral quantities to be translated to a particular structural scale once the reference quantities $M_{0}$, $T_{0}$, and $L_{0}$ are specified. Nevertheless, the present parameter set is not calibrated to a specific engineering device. The results should therefore be used to identify robust tuning trends and sensitive operating regions, while device-specific design predictions require application-dependent dimensional parameters and experimental or literature-based calibration.

### 4.3. Interpretation of the Reference-Modal Coordinates

The fixed-reference modal coordinates should be interpreted as a coordinate representation rather than as a finite-dimensional modal decomposition of the DDE state space. Because the present prototype has two physical generalized coordinates, the transformation based on $\Phi_{*}$ retains both physical coordinates and is therefore not a physical-coordinate truncation. Nevertheless, the delay differential equation remains infinite-dimensional through its history state and its quasi-polynomial characteristic spectrum. The reference basis diagonalizes only the selected conservative operator at $\alpha =\alpha_{*}$. The delayed, damped, and detuned operators are not simultaneously diagonalized, and their off-diagonal terms are retained in the analysis. These terms provide a convenient coordinate-level interpretation of branch interaction and branch-energy exchange. The diagonal-only system used in Figure 6 is therefore only a diagnostic approximation, not the modal form employed for the reported spectral calculations.

### 4.4. Scope and Limitations

The interpretation has several explicit limitations. First, the delay-induced stability boundary of the deterministic linearized DDE is obtained from the analytical imaginary-axis-crossing criterion and independently checked by direct multi-start characteristic-root searches and Chebyshev-collocation calculations. The criterion characterizes the local spectral-stability boundary of the linearized delayed system; it does not constitute a global stability theorem for the nonlinear stochastic model. Second, the nonlinear analysis uses a weakly nonlinear first-harmonic backbone approximation, rather than a full nonlinear bifurcation analysis. Third, the frozen spectral and OU-driven time-domain stochastic frameworks share the same positive unit-mean lognormal one-time marginal distribution, but they retain different temporal information. The frozen formulation treats the multiplier as realization-wise constant and quantifies static changes in the tracked characteristic roots and veering metrics. The OU formulation retains temporal correlation and path dependence and is used to evaluate finite-time branch-residence and delayed-work statistics of the nonlinear DDE. Accordingly, quantities such as $\mathrm{Pr}(m_{\mathrm{tr}}>0)$ or $P_{+}$ should not be identified numerically with $P_{\mathrm{TD}}$; the two frameworks provide complementary static-spectral and dynamic-pathwise views of the same stochastic delayed-coupling mechanism. Fourth, the Monte Carlo residence probability is a finite-time, threshold-dependent path observable rather than an invariant-measure switching probability or a stochastic bifurcation criterion. The threshold-sensitivity analysis shows that this observable can become saturated near the veering core but remains substantially threshold-dependent away from the core. Its numerical value should therefore be interpreted only for the stated thresholds, initial history, and observation window. The threshold-independent normalized reference-modal energy fractions introduced above provide a complementary measure of branch participation. Fifth, the dimensional relations in Equations (17)–(21) provide a consistent mapping from the nondimensional benchmark to physical mass, damping, stiffness, delay, and displacement scales. However, no unique dimensional realization is claimed because the model is not calibrated to a particular experimental structure. Device-specific quantitative predictions therefore require the reference scales and physical coefficients to be identified from the target application. Future work may extend the analysis to higher-dimensional structures, distributed-delay kernels, stronger nonlinear regimes, and coupling fluctuations measured experimentally.

## 5. Conclusions

The study develops a mechanism-oriented investigation of delay-modulated stochastic nonlinear mode veering in an inertially coupled vibration oscillator. At the deterministic limit, the model connects naturally to stiffness–inertial coupled veering systems. The delayed model yields a complex-root veering landscape governed by the quasi-polynomial characteristic equation. For the deterministic linearized DDE, the corresponding delay-stability boundary is determined by an analytical imaginary-axis-crossing criterion and is independently reproduced by the direct multi-start and Chebyshev-collocation calculations. The fixed-reference modal-coordinate representation connects this spectral behavior to branch-energy indicators and retained off-diagonal modal coupling without diagonalizing the full DDE operator. The delayed cubic coupling produces an amplitude-dependent nonlinear backbone deformation, while positive multiplicative stochastic modulation broadens the root distribution and randomizes the veering center. The pathwise energy balance explains why the delayed relative-coordinate work rates act as sign-indefinite energy-transfer channels. Finally, the frozen and OU-driven stochastic formulations are linked through the same positive unit-mean lognormal coupling marginal but retain different temporal information. The frozen calculation quantifies static spectral broadening, whereas the OU Monte Carlo simulations show its trajectory-level counterpart in finite-time residence-based branch-energy exchange. A quantitative energy decomposition further shows that, for the prescribed finite-time ensemble, the second reference-modal branch carries approximately 90.5–99.9% of the reference-modal energy indicator, while the linear delayed-coupling term contributes approximately 60.0–63.3% of the total absolute-work activity and constitutes the largest single pathwise energy-transfer channel. Taken together, these results provide a vibration-mechanics explanation of how delay, nonlinearity, and stochastic coupling fluctuations reshape mode veering in inertially coupled systems. The dimensional back-mapping further allows these nondimensional results to be transferred consistently to different physical structural scales through application-specific choices of $M_{0}$, $T_{0}$, and $L_{0}$, without implying calibration to a unique engineering device.From an engineering perspective, these results indicate that coupling delay, operating amplitude, and stochastic coupling uncertainty should be considered jointly when tuning inertially coupled attachments or delayed vibration-control devices. In particular, the nominal operating point should retain sufficient spectral margin against both amplitude-dependent shifts and uncertainty-induced broadening rather than being selected solely from a deterministic zero-delay model.

**Proposition 6** 
(Scoped conclusion of the mechanism chain)**.** *Within the dimensionless benchmark and the tested parameter window, the evidence points to the following scoped implication. Delayed relative-coordinate coupling gives rise to a complex-root veering structure. Fixed-reference modal coordinates identify the retained off-diagonal terms associated with branch interaction. A first-harmonic treatment of the cubic coupling deforms the veering backbone. Positive lognormal coupling perturbations broaden the tracked spectral cloud. OU time-domain simulations show a corresponding finite-time residence response governed by sign-indefinite delayed work. Beyond the exact algebraic identities stated earlier, this implication rests on empirical-numerical grounds and does not constitute a global stochastic bifurcation theorem.*

## Data Availability

The accompanying reproducibility package contains the code, parameters, generated data, and locked figure files for Figure 2, Figure 3, Figure 4, Figure 5, Figure 6, Figure 7, Figure 8, Figure 9, Figure 10 and Figure 11. The package also contains the updated Supplementary Materials, source scripts, CSV/NPZ data files, and robustness figures for the stochastic time-domain checks.

## Supplementary Materials

The following supporting information can be downloaded at: https://www.mdpi.com/article/10.3390/e28090952/s1, Figure S1: First-switching survival curves at α=0.97 for dynamically evolving OU coupling modulation; Figure S2: Time-step robustness of the time-domain exchange-active probability and mean branch-2 residence fraction; Figure S3: Monte Carlo sample-size sensitivity of the residence-based statistics; Figure S4: Supplementary linear-response evidence near the veering region; Figure S5: Supplementary nonlinear-response evidence; Figure S6: Supplementary stochastic-response evidence; Figure S7: Delay-sensitive veering center, gap, and rightmost boundary; Figure S8: Revised frozen stochastic spectral-cloud visualization; Figure S9: Time-domain stochastic DDE Monte Carlo validation.
