# Supplementary material for: Delay-Modulated Nonlinear Stochastic Mode Veering in Inertially Coupled Vibration Systems
Source: Entropy (Basel). 2026 Aug 24;28(9):952. doi: 10.3390/e28090952 (PMC13604981; doi:10.3390/e28090952)
Supplement: Supplementary file 1 [file entropy-28-00952-s001.zip › figures/Fig3_delay_boundary_revised.pdf]

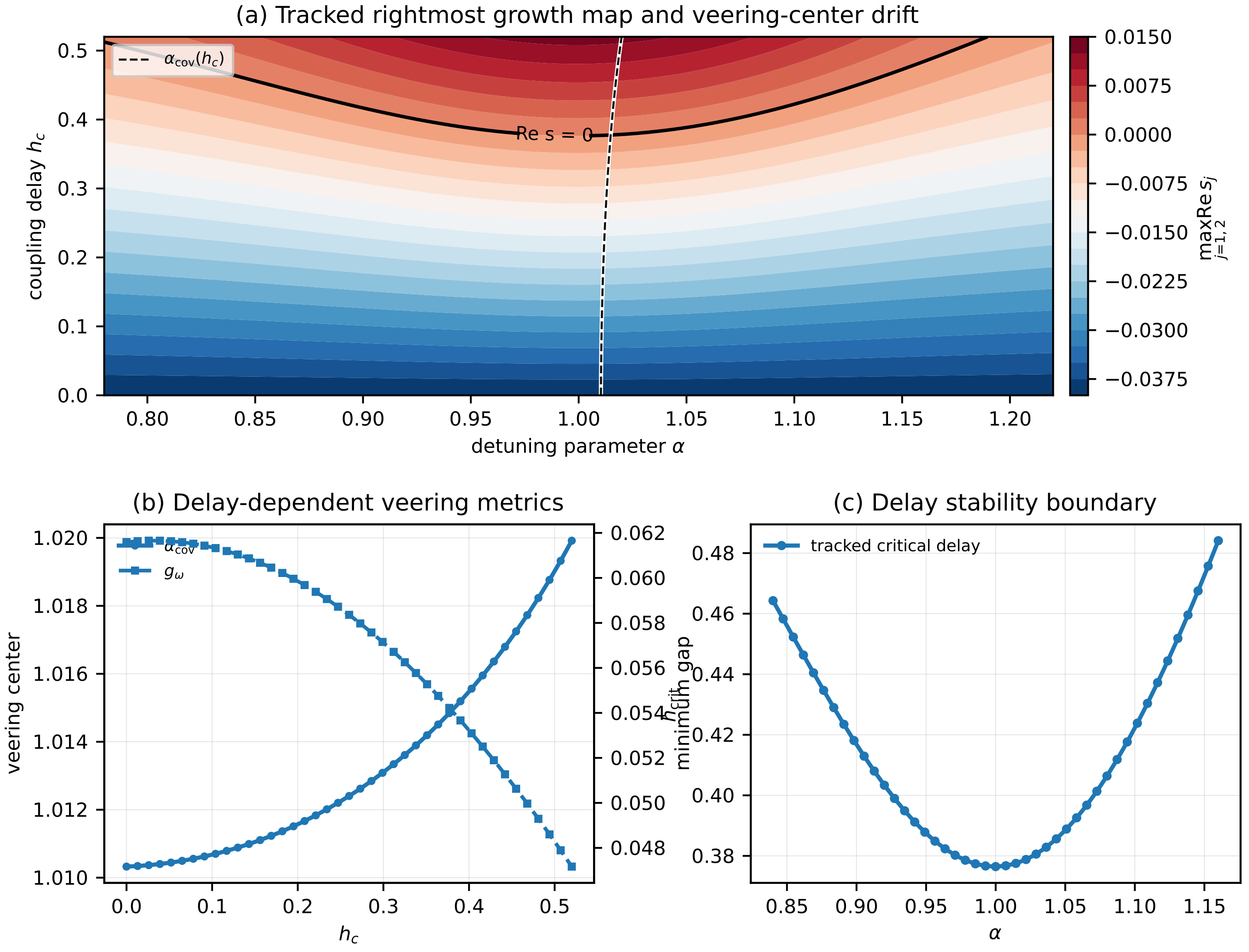

(a) Tracked rightmost growth map and veering-center drift
coupling delay h_c
detuning parameter α
$\alpha_{\mathrm{cov}}(h_c)$
Re s = 0
$\max_{j=1,2} \mathrm{Re}\, s_j$
0.0150
0.0075
0.0000
−0.0075
−0.0150
−0.0225
−0.0300
−0.0375
(b) Delay-dependent veering metrics
veering center
minimum gap
h_c
α_{cov}
g_ω
(c) Delay stability boundary
h_{crit}
α
tracked critical delay
