# Supplementary material for: Delay-Modulated Nonlinear Stochastic Mode Veering in Inertially Coupled Vibration Systems
Source: Entropy (Basel). 2026 Aug 24;28(9):952. doi: 10.3390/e28090952 (PMC13604981; doi:10.3390/e28090952)
Supplement: Supplementary file 1 [file entropy-28-00952-s001.zip › figures/Fig6_stochastic_spectral_revised.pdf]

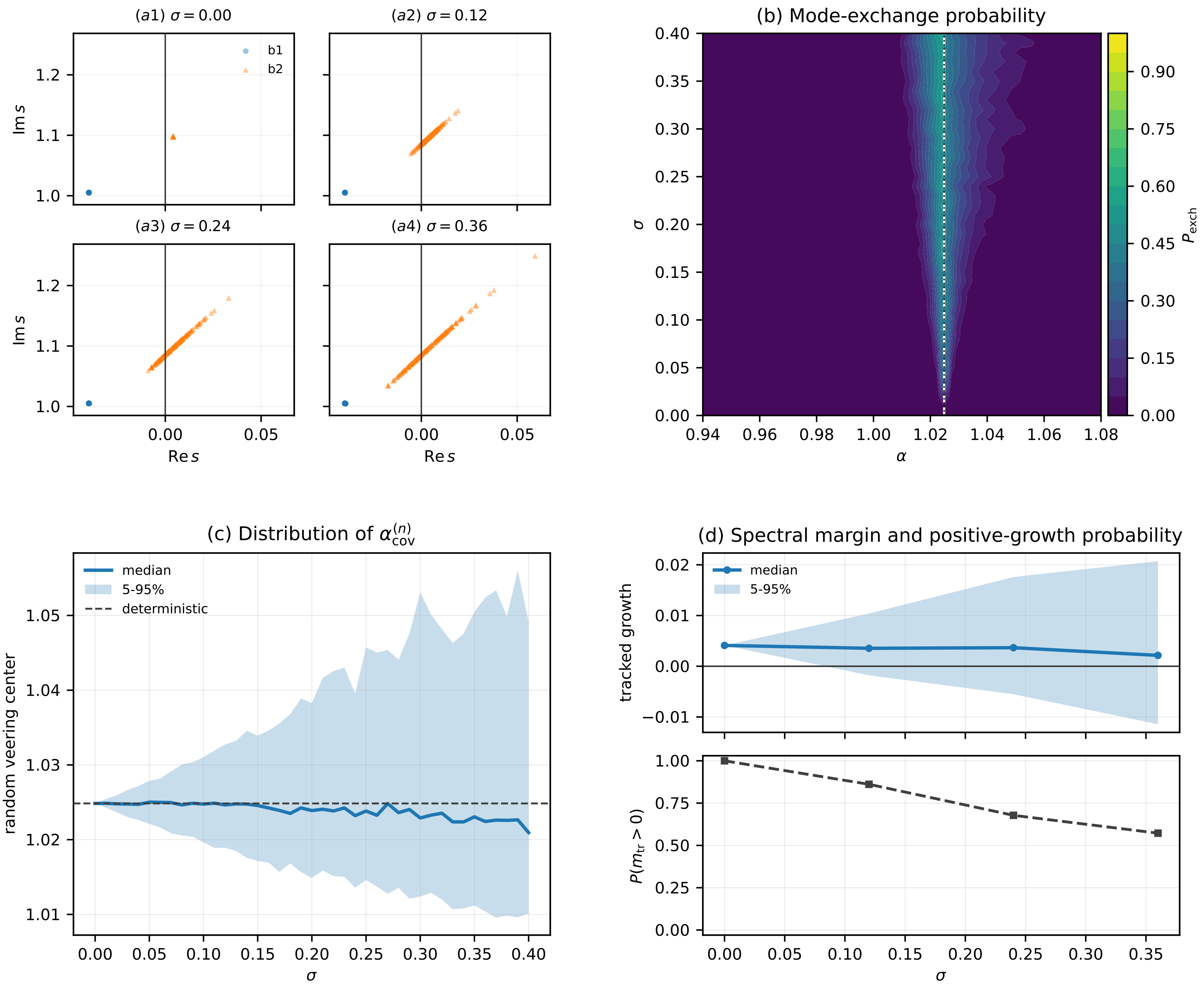

(a1) σ = 0.00
(a2) σ = 0.12
(a3) σ = 0.24
(a4) σ = 0.36
b1
b2
Im s
Re s
(b) Mode-exchange probability
σ
α
P_exch
(c) Distribution of α_cov^(n)
median
5-95%
deterministic
random veering center
σ
(d) Spectral margin and positive-growth probability
median
5-95%
tracked growth
P(m_tr > 0)
σ
