# Supplementary material for: Delay-Modulated Nonlinear Stochastic Mode Veering in Inertially Coupled Vibration Systems
Source: Entropy (Basel). 2026 Aug 24;28(9):952. doi: 10.3390/e28090952 (PMC13604981; doi:10.3390/e28090952)
Supplement: Supplementary file 1 [file entropy-28-00952-s001.zip › figures/Fig7_time_domain_stochastic_revised.pdf]

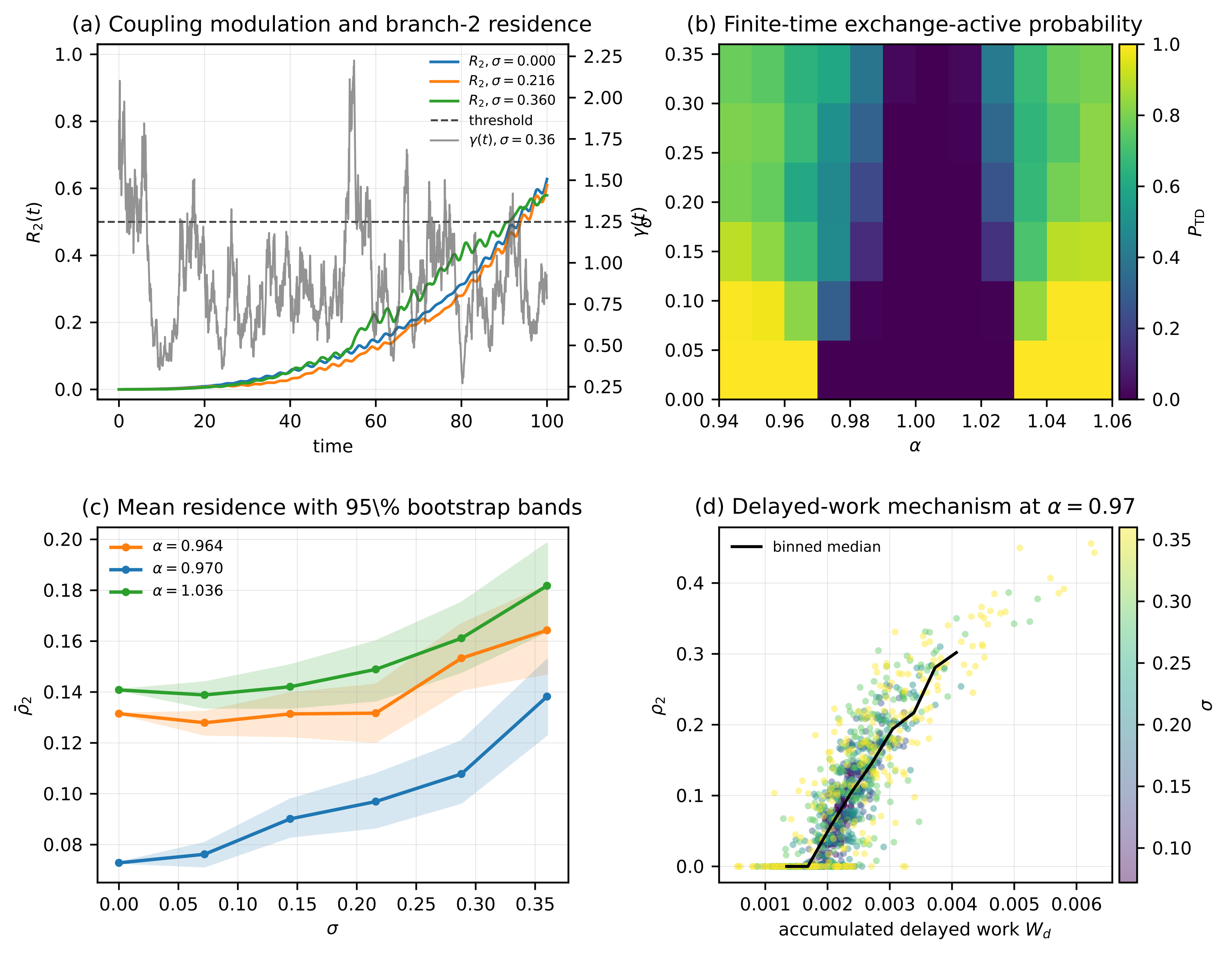

(a) Coupling modulation and branch-2 residence
$R_2, \sigma = 0.000$
$R_2, \sigma = 0.216$
$R_2, \sigma = 0.360$
threshold
$\gamma(t), \sigma = 0.36$
$R_2(t)$
$\gamma(t)$
time
(b) Finite-time exchange-active probability
σ
α
P_{TD}
(c) Mean residence with 95\% bootstrap bands
$\alpha = 0.964$
$\alpha = 0.970$
$\alpha = 1.036$
$\bar{\rho}_2$
σ
(d) Delayed-work mechanism at $\alpha = 0.97$
binned median
ρ_2
accumulated delayed work W_d
σ
