# Supplementary material for: Delay-Modulated Nonlinear Stochastic Mode Veering in Inertially Coupled Vibration Systems
Source: Entropy (Basel). 2026 Aug 24;28(9):952. doi: 10.3390/e28090952 (PMC13604981; doi:10.3390/e28090952)
Supplement: Supplementary file 1 [file entropy-28-00952-s001.zip › figures/first_switch_survival_curves.pdf]

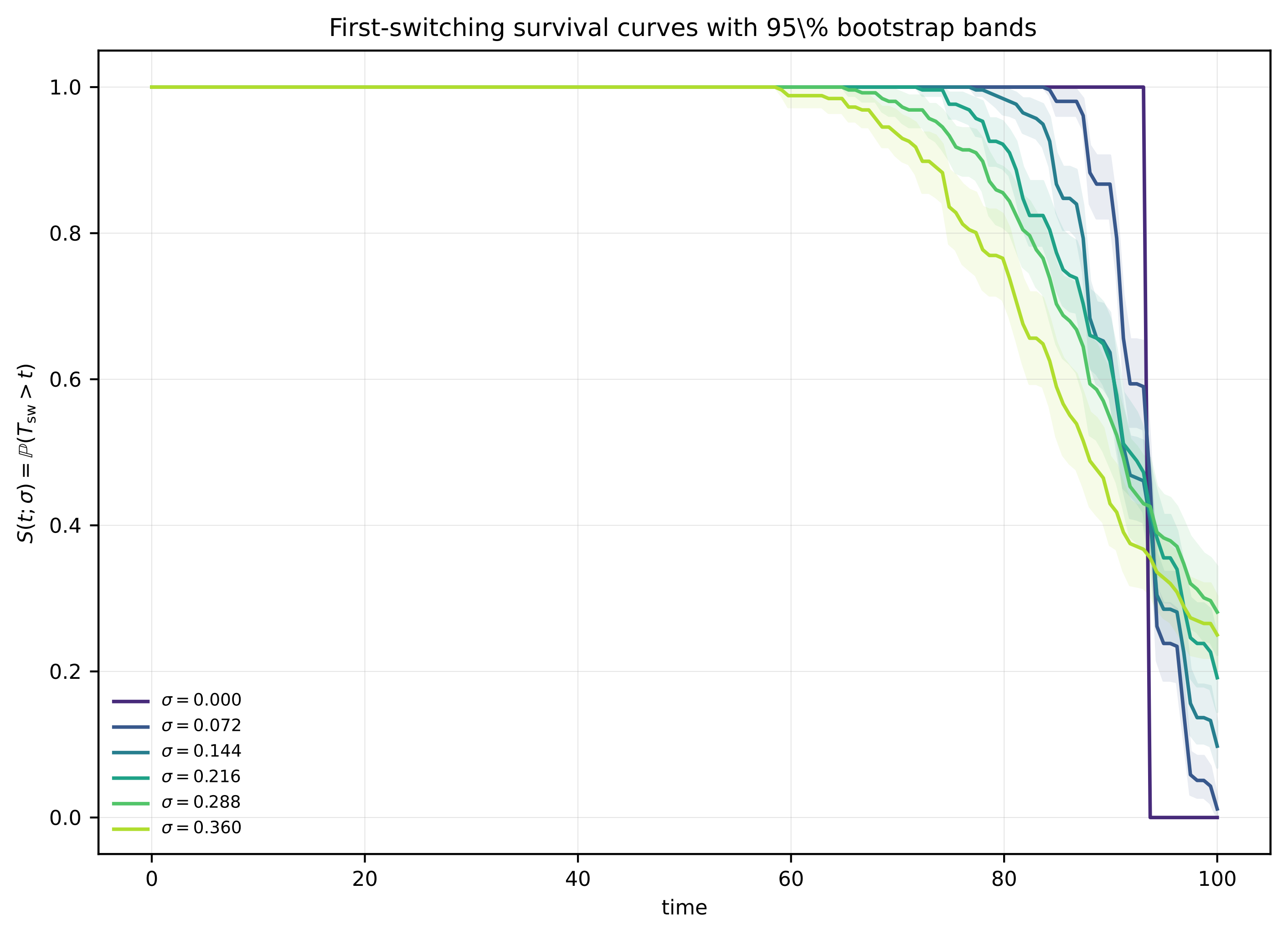

First-switching survival curves with 95\% bootstrap bands
$S(t;\sigma) = \mathbb{P}(T_{sw} > t)$
time
0.0
0.2
0.4
0.6
0.8
1.0
0
20
40
60
80
100
$\sigma = 0.000$
$\sigma = 0.072$
$\sigma = 0.144$
$\sigma = 0.216$
$\sigma = 0.288$
$\sigma = 0.360$
