# Supplementary material for: Delay-Modulated Nonlinear Stochastic Mode Veering in Inertially Coupled Vibration Systems
Source: Entropy (Basel). 2026 Aug 24;28(9):952. doi: 10.3390/e28090952 (PMC13604981; doi:10.3390/e28090952)
Supplement: Supplementary file 1 [file entropy-28-00952-s001.zip › figures/robustness_dt_convergence.pdf]

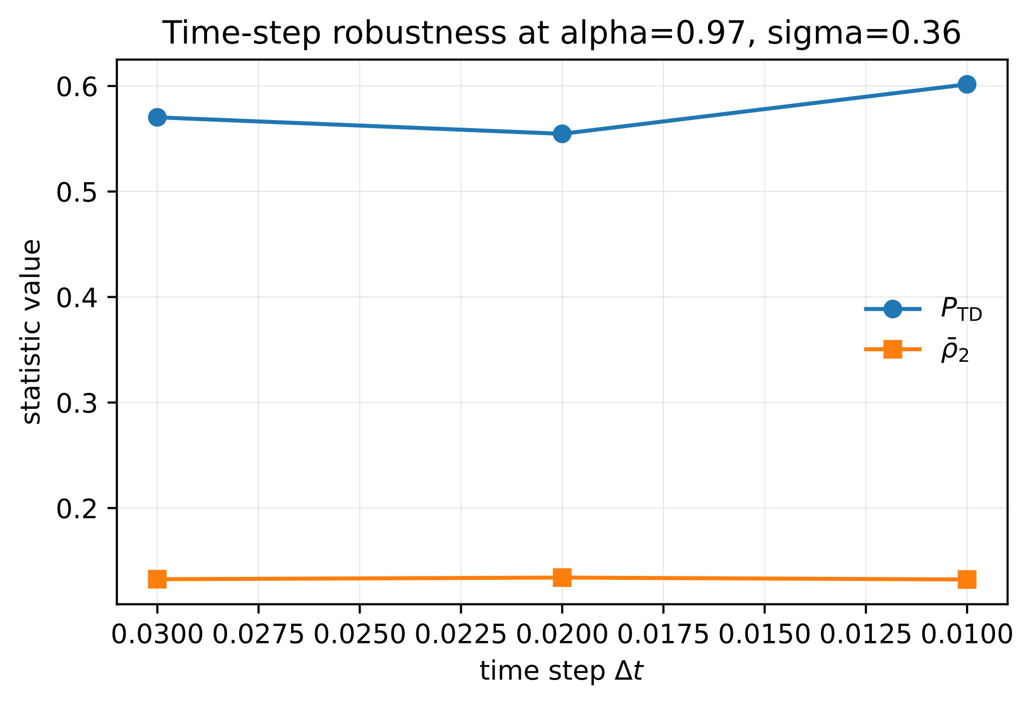
Time-step robustness at alpha=0.97, sigma=0.36
statistic value
0.2
0.3
0.4
0.5
0.6
0.0300
0.0275
0.0250
0.0225
0.0200
0.0175
0.0150
0.0125
0.0100
time step Δt
P_{TD}
$\bar{\rho}_2$
