# Supplementary material for: Delay-Modulated Nonlinear Stochastic Mode Veering in Inertially Coupled Vibration Systems
Source: Entropy (Basel). 2026 Aug 24;28(9):952. doi: 10.3390/e28090952 (PMC13604981; doi:10.3390/e28090952)
Supplement: Supplementary file 1 [file entropy-28-00952-s001.zip › figures/Supp_linear_response_evidence.pdf]

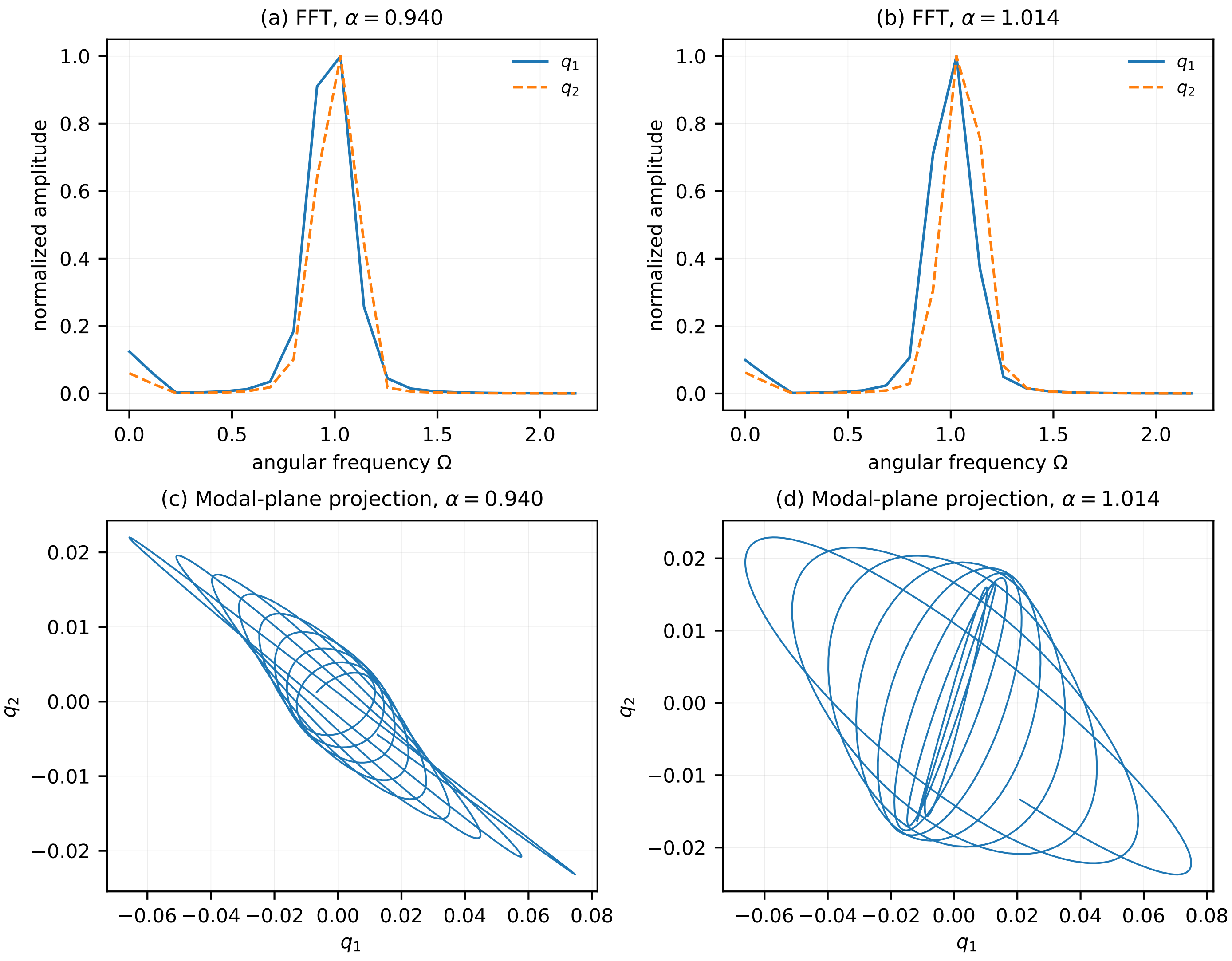

(a) FFT, $\alpha = 0.940$
(b) FFT, $\alpha = 1.014$
normalized amplitude
angular frequency Ω
q_1
q_2
0.0
0.2
0.4
0.6
0.8
1.0
0.5
1.5
2.0
(c) Modal-plane projection, $\alpha = 0.940$
(d) Modal-plane projection, $\alpha = 1.014$
−0.06
−0.04
−0.02
0.00
0.02
0.04
0.06
0.08
−0.01
0.01
