# Supplementary material for: Delay-Modulated Nonlinear Stochastic Mode Veering in Inertially Coupled Vibration Systems
Source: Entropy (Basel). 2026 Aug 24;28(9):952. doi: 10.3390/e28090952 (PMC13604981; doi:10.3390/e28090952)
Supplement: Supplementary file 1 [file entropy-28-00952-s001.zip › figures/Supp_nonlinear_response_evidence.pdf]

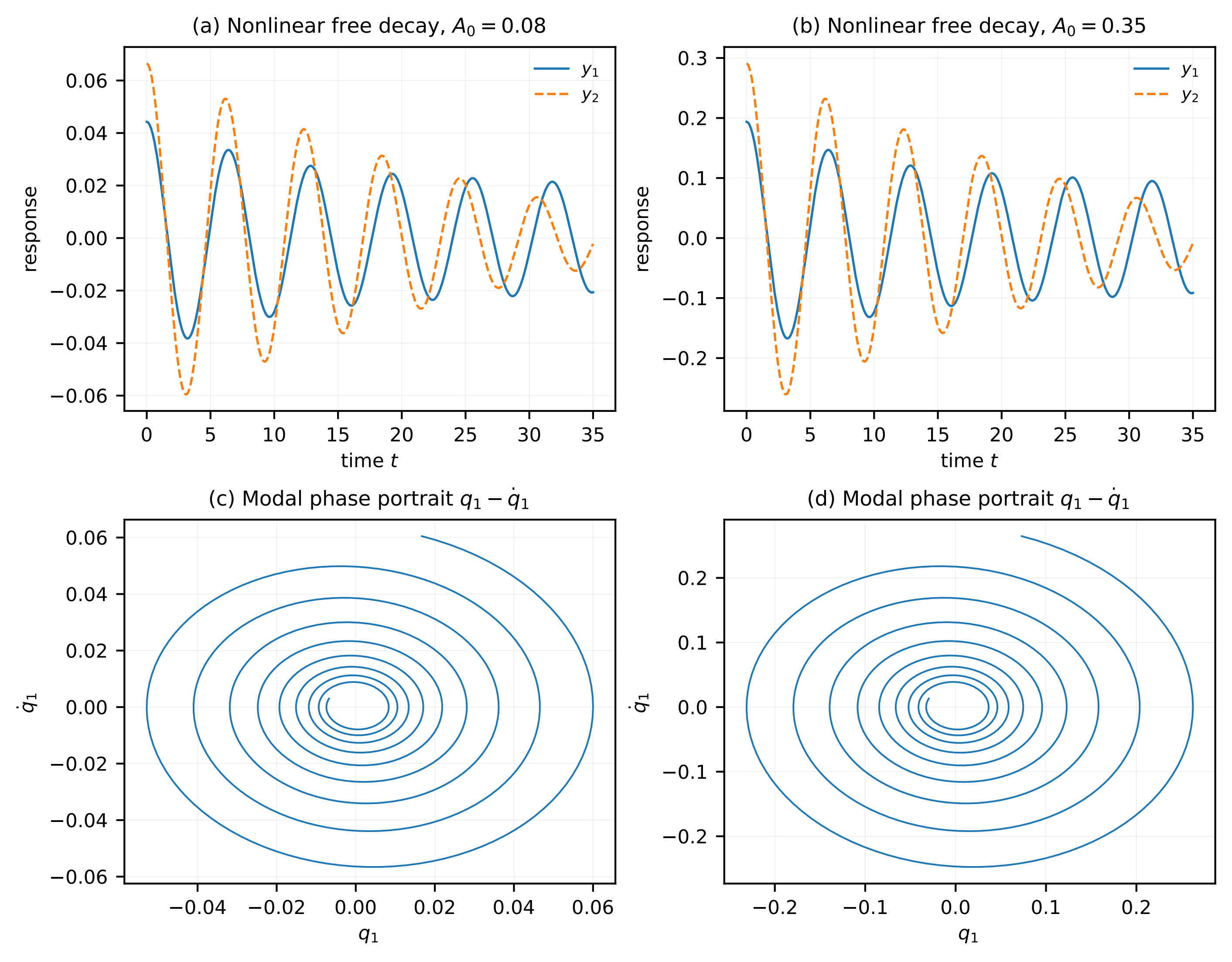
(a) Nonlinear free decay, $A_0 = 0.08$
(b) Nonlinear free decay, $A_0 = 0.35$
(c) Modal phase portrait $q_1 - \dot{q}_1$
(d) Modal phase portrait $q_1 - \dot{q}_1$
response
time t
y_1
y_2
q_1
$\dot{q}_1$
0.06
0.04
0.02
0.00
−0.02
−0.04
−0.06
0.3
0.2
0.1
0.0
−0.1
−0.2
0
5
10
15
20
25
30
35
