# Supplementary material for: Delay-Modulated Nonlinear Stochastic Mode Veering in Inertially Coupled Vibration Systems
Source: Entropy (Basel). 2026 Aug 24;28(9):952. doi: 10.3390/e28090952 (PMC13604981; doi:10.3390/e28090952)
Supplement: Supplementary file 1 [file entropy-28-00952-s001.zip › figures/Supp_stochastic_response_evidence.pdf]

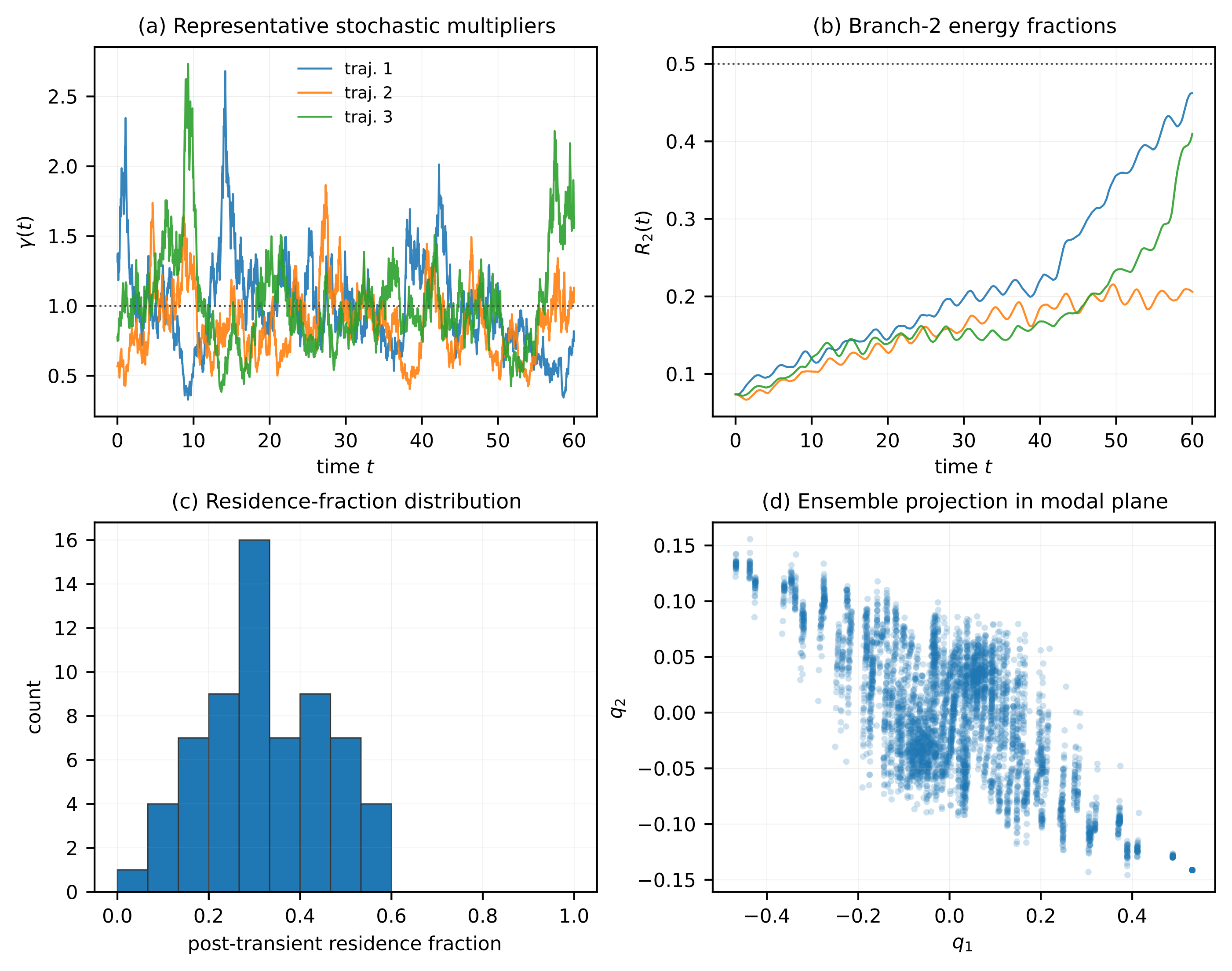

(a) Representative stochastic multipliers
traj. 1
traj. 2
traj. 3
$\gamma(t)$
time t
(b) Branch-2 energy fractions
$R_2(t)$
time t
(c) Residence-fraction distribution
count
post-transient residence fraction
(d) Ensemble projection in modal plane
q_2
q_1
