# Supplementary material for: Delay-Modulated Nonlinear Stochastic Mode Veering in Inertially Coupled Vibration Systems
Source: Entropy (Basel). 2026 Aug 24;28(9):952. doi: 10.3390/e28090952 (PMC13604981; doi:10.3390/e28090952)
Supplement: Supplementary file 1 [file entropy-28-00952-s001.zip › supplementary.pdf]

Supplementary Material: Delay-Modulated Nonlinear Stochastic Mode Veering in Inertially Coupled Vibration Systems

Lili Zhang Zikun Han Qiubao Wang

1 Unified model and notation

The physical-coordinate model used in the main text is

$$M(\rho)\ddot{y}(t) + C\dot{y}(t) + K_g(\alpha)y(t) + k_d\tau(t)K_c y(t-h_c) + \kappa_3 b[b^T y(t-h_c)]^3 = 0.$$

Here $b = (-1,1)^T$, $K_c = bb^T$, and $z(t) = b^T y(t)$ is the relative displacement. The multiplicative stochastic coupling is

$$\tau(t) = \tau_0 \exp\left(\sigma\eta(t) - \frac{\sigma^2}{2}\right).$$

This choice keeps the coupling positive. The exponential factor has unit stationary mean, so that $\mathbb{E}[\tau(t)] = \tau_0$ under stationary Gaussian sampling.

2 Deterministic inertial-coupling limit

The deterministic baseline is obtained by setting

$$h_c = 0, \qquad \kappa_3 = 0, \qquad \sigma = 0.$$

The governing equation then reduces to

$$M(\rho)\ddot{y} + C\dot{y} + [K_g(\alpha) + k_d\tau_0 K_c]\, y = 0.$$

Suppressing damping gives the conservative eigenvalue problem

$$\det\left[-\omega^2 M(\rho) + K_g(\alpha) + k_d\tau_0 K_c\right] = 0.$$

With

$$\tau = \frac{k_d\tau_0}{k},$$

the deterministic stiffness matrix is

$$K_{\text{det}}(\alpha,\tau) = k\begin{bmatrix} 1+\tau & -\tau \\ -\tau & \alpha+\tau \end{bmatrix}.$$

The inertia matrix is

$$M(\rho) = mI + \rho bb^T = \begin{bmatrix} m+\rho & -\rho \\ -\rho & m+\rho \end{bmatrix}.$$

Therefore the current model contains the conservative inertial-coupled two-coordinate veering problem as a limiting case. The delay, cubic, and stochastic terms in the main text should be read as unfoldings of this deterministic baseline.

3 Newtonian component-wise verification

The Lagrange–d'Alembert derivation in the main text can be checked directly in component form. Using

$$M(\rho) = \begin{bmatrix} m+\rho & -\rho \\ -\rho & m+\rho \end{bmatrix}, \qquad C = \begin{bmatrix} c_g + c_c & -c_c \\ -c_c & c_g + c_c \end{bmatrix},$$

and

$$z_h = y_2(t-h_c) - y_1(t-h_c),$$

the governing equation is equivalent to

$$(m+\rho)\ddot{y}_1 - \rho\ddot{y}_2 + c_g\dot{y}_1 + c_c(\dot{y}_1 - \dot{y}_2) + ky_1 - k_d\tau(t)z_h - \kappa_3 z_h^3 = 0, \tag{1}$$

$$-\rho\ddot{y}_1 + (m+\rho)\ddot{y}_2 + c_g\dot{y}_2 + c_c(\dot{y}_2 - \dot{y}_1) + k\alpha y_2 + k_d\tau(t)z_h + \kappa_3 z_h^3 = 0. \tag{2}$$

These two equations verify the signs of the inertial coupling, relative damping, delayed linear coupling, and delayed cubic coupling. For example, if $z_h > 0$, the delayed coupling terms pull y_1 toward y_2 and y_2 toward y_1, as expected for a restoring relative coupling. They also confirm that $y = 0$ remains an equilibrium under multiplicative stochastic modulation.

4 Linear delayed characteristic equation and branch definitions

The deterministic linear backbone used for Figs. 2–3 is

$$M\ddot{y}(t) + C\dot{y}(t) + K_g(\alpha)y(t) + K_d y(t-h_c) = 0, \qquad K_d = k_d\tau_0 K_c.$$

With $x = (y^T, \dot{y}^T)^T$, it is written as

$$\dot{x}(t) = A_0(\alpha)x(t) + A_1 x(t-h_c),$$

where

$$A_0(\alpha) = \begin{bmatrix} 0 & I \\ -M^{-1}K_g(\alpha) & -M^{-1}C \end{bmatrix}, \qquad A_1 = \begin{bmatrix} 0 & 0 \\ -M^{-1}K_d & 0 \end{bmatrix}.$$

The characteristic matrix is

$$\mathcal{D}(s;\alpha,h_c) = sI - A_0(\alpha) - A_1 e^{-sh_c},$$

and the characteristic equation is

$$\Delta(s;\alpha,h_c) = \det \mathcal{D}(s;\alpha,h_c) = 0.$$

Equivalently,

$$\det\left[s^2 M + sC + K_g(\alpha) + K_d e^{-sh_c}\right] = 0.$$

The two tracked roots are the two positive-imaginary roots nearest the veering interaction,

$$s_j(\alpha,h_c) = \mu_j(\alpha,h_c) + i\omega_j(\alpha,h_c), \qquad j = 1,2,$$

ordered by $\omega_1 \le \omega_2$. The veering center and gap are computed as

$$\alpha_{\mathrm{cov}}(h_c) = \arg\min_{\alpha}[\omega_2(\alpha,h_c) - \omega_1(\alpha,h_c)],$$

$$g_\omega(h_c) = \min_{\alpha}[\omega_2(\alpha,h_c) - \omega_1(\alpha,h_c)].$$

The minimizer is refined by a local quadratic interpolation around the smallest grid value, so the reported α_{cov} is not merely the nearest grid point.

Root-continuation procedure

For the first value of α, the positive-imaginary roots of the corresponding non-delayed or weakly delayed first-order system are used as initial guesses. At the next parameter value, the previously converged roots are used as predictors. For each root, the real two-dimensional nonlinear system

$$\operatorname{Re}\Delta(s;\alpha,h_c)=0,\qquad \operatorname{Im}\Delta(s;\alpha,h_c)=0$$

is solved for $s=\mu+i\omega$. A root is accepted only when the nonlinear solver converges and the residual $|\Delta(s;\alpha,h_c)|$ is below the numerical tolerance. The largest residual in the main-text Figure 2 calculation is approximately 1.16×10^{-14}, and the largest residual in the main-text Figure 3 delay-metric calculation is approximately 1.63×10^{-14}.

Representative numerical values

The delay scan gives

h_c	0	0.10	0.20	0.30	0.40	0.52
α_{cov}	1.0103	1.0107	1.0116	1.0131	1.0155	1.0199
g_ω	0.0616	0.0614	0.0599	0.0571	0.0532	0.0472
$\max\operatorname{Re}s$	−0.0400	−0.0291	−0.0183	−0.0077	0.0023	0.0136

The tracked critical-delay curve has its minimum near

$$\alpha=1.0,\qquad h_{\text{crit}}\approx0.3765,\qquad \omega_{\text{crit}}\approx1.0537.$$

5 Rightmost-spectrum validation

The stability indicator for the linear DDE is the spectral abscissa

$$a(\alpha,h_c)=\sup\{\operatorname{Re}s:\Delta(s;\alpha,h_c)=0\}.$$

A tracked veering-related branch can be used as a candidate rightmost branch, but this alone is not sufficient to claim stability. Therefore, the analytical critical-delay boundary reported in main-text Figure 3 was independently checked by two numerical procedures.

Direct multi-start quasi-polynomial search

The first check solves

$$\operatorname{Re}\Delta(s;\alpha,h_c)=0,\qquad \operatorname{Im}\Delta(s;\alpha,h_c)=0$$

from a grid of initial guesses in the complex plane. The roots are clustered to remove duplicates, and the root with largest real part is selected as the direct-search rightmost root. At the validation points, the maximum difference between the direct-search rightmost root and the tracked rightmost root is approximately 1.02×10^{-15}, with no stability-sign mismatch.

Chebyshev-collocation generator approximation

The second check approximates the infinitesimal generator of the retarded DDE using Chebyshev collocation on the delay interval. The resulting finite-dimensional matrix has eigenvalues that approximate the rightmost DDE spectrum. The collocation root nearest to the tracked rightmost root differs by at most 3.76×10^{-13} in the tested validation set, and the stability classification agrees with the tracked branch at all validation points.

Scope of validation

The analytical imaginary-axis-crossing criterion determines the local critical-delay boundary of the deterministic linearized DDE. Within the tested parameter window, the direct multi-start quasi-polynomial search and the Chebyshev-collocation generator approximation independently reproduce this boundary and identify the same rightmost-root classification. This agreement provides numerical validation of the analytical spectral boundary in the investigated parameter range. It should not be interpreted as a global stability proof for arbitrary delays, parameter values, or for the nonlinear stochastic system.

6 Fixed-reference modal-coordinate representation and branch-energy diagnostic

6.1 Coordinate equivalence for the two-coordinate prototype

For the present two-coordinate physical prototype, the fixed-reference modal transformation is algebraically exact at the level of the two physical-coordinate equations and should not be described as a lossy reduced-order approximation. Let α_* be the reference detuning at the veering core and define

$$K_*(\alpha_*) = K_g(\alpha_*) + K_d.$$

The reference modes are obtained from

$$K_*(\alpha_*)\phi_{j*} = \omega_{j*}^2 M \phi_{j*}, \qquad j = 1, 2,$$

with mass normalization

$$\Phi_*^T M \Phi_* = I, \qquad \Phi_*^T K_*(\alpha_*)\Phi_* = \mathrm{diag}(\omega_{1*}^2, \omega_{2*}^2).$$

Since $\Phi_* \in \mathbb{R}^{2\times 2}$ is nonsingular,

$$y(t) = \Phi_* q(t), \qquad q(t) = \Phi_*^T M y(t)$$

is an invertible coordinate transformation. No physical coordinate is discarded. However, because the delay differential equation remains infinite-dimensional through its history state, this transformation should not be interpreted as a complete modal decomposition of the DDE state space or as a diagonalization of the delay operator.

The reference detuning is computed from the conservative veering core:

$$\alpha_* = \arg\min_{\alpha\in\mathcal{I}_\alpha} \left[\omega_2^0(\alpha) - \omega_1^0(\alpha)\right],$$

where $\omega_j^0(\alpha)$ are obtained from

$$[K_g(\alpha) + K_d]\phi = \omega^2 M \phi.$$

Thus α_*, Φ_*, and ω_{j*} are determined by the original physical matrices M, $K_g(\alpha)$, and K_d, not by a separate fitting procedure.

The modal equation is

$$M_q\ddot{q}(t) + C_q\dot{q}(t) + K_q(\alpha)q(t) + K_{d,q}q(t - h_c) = 0,$$

where

$$M_q = \Phi_*^T M \Phi_* = I, \qquad C_q = \Phi_*^T C \Phi_*,$$
$$K_q(\alpha) = \Phi_*^T K_g(\alpha)\Phi_*, \qquad K_{d,q} = \Phi_*^T K_d \Phi_*.$$

In component form, if $\Phi_* = (\phi_{ri})$, then

$$[K_q(\alpha)]_{ij} = k\phi_{1i}\phi_{1j} + k\alpha\phi_{2i}\phi_{2j},$$

$$[K_{d,q}]_{ij} = k_d\tau_0(\phi_{2i} - \phi_{1i})(\phi_{2j} - \phi_{1j}),$$

and

$$[C_q]_{ij} = c_g(\phi_{1i}\phi_{1j} + \phi_{2i}\phi_{2j}) + c_c(\phi_{2i} - \phi_{1i})(\phi_{2j} - \phi_{1j}).$$

These formulas give the explicit dependence of all modal matrices on the original physical parameters and on the computed basis Φ_*.

The modal characteristic equation is

$$\det\left[s^2 M_q + sC_q + K_q(\alpha) + K_{d,q}e^{-sh_c}\right] = 0.$$

Because the transformation is invertible, this equation has the same roots as the physical-coordinate characteristic equation. The exponential delay factor and the associated infinite characteristic spectrum are retained unchanged.

6.2 Role of the diagonal-only comparison

The diagonal-only curves in main-text Figure 6 are not used as the working model. They are a diagnostic approximation obtained by deleting off-diagonal modal coupling terms. Their failure to reproduce the critical-delay curve shows that veering is controlled by coupled modal dynamics, not by two independent scalar modal equations. In the numerical verification, the physical-coordinate and exact modal-coordinate roots agree to round-off accuracy, whereas the diagonal-only approximation produces a finite error in the critical-delay curve.

6.3 Numerical modal quantities used in main-text Figure 6

The actual numerical quantities used in the main-text Figure 6 computation are:

$$\alpha_* = 1.010324863, \qquad \omega_* = \begin{bmatrix} 1.002482744 \\ 1.064028490 \end{bmatrix}.$$

The mass-normalized modal basis is

$$\Phi_* = \begin{bmatrix} 0.732688 & 0.646308 \\ 0.680464 & -0.70108 \end{bmatrix}.$$

The corresponding modal matrices are

$$M_q = \begin{bmatrix} 1 & -1.87048e-16 \\ -9.62684e-17 & 1 \end{bmatrix}, \qquad C_q = \begin{bmatrix} 0.08 & -1.74648e-17 \\ -1.10749e-17 & 0.08 \end{bmatrix},$$

$$K_q(\alpha_*) = \begin{bmatrix} 1.00464 & -0.00844385 \\ -0.00844385 & 0.914302 \end{bmatrix}, \qquad K_{d,q} = \begin{bmatrix} 0.000327276 & 0.00844385 \\ 0.00844385 & 0.217855 \end{bmatrix}.$$

Finally,

$$K_q(\alpha_*) + K_{d,q} = \begin{bmatrix} 1.00497 & -5.77778e-17 \\ -1.20184e-16 & 1.13216 \end{bmatrix},$$

which is diagonal to round-off error, as required by the reference normalization.

6.4 Branch-energy indicator

For time-domain simulations, modal branch residence is quantified by a reference energy indicator. For $q(t)$ and $\dot{q}(t)$, with

$$q(t) = \Phi_*^T M y(t), \qquad \dot{q}(t) = \Phi_*^T M \dot{y}(t),$$

define

$$E_j(t) = \frac{1}{2}\dot{q}_j^2(t) + \frac{1}{2}\omega_{j*}^2 q_j^2(t), \qquad j = 1, 2.$$

The corresponding branch-energy fraction is

$$R_j(t) = \frac{E_j(t)}{E_1(t) + E_2(t) + \varepsilon_E}, \qquad j = 1, 2.$$

The small ε_E is used only for numerical regularization near the zero state. In the nondimensional simulations, this is implemented by replacing the denominator with $\max(E_1 + E_2, 10^{-30})$, and residence statistics are accumulated only when the total reference modal energy remains above the prescribed numerical floor. Since the actual system contains damping, delay, nonlinearity, and stochastic modulation, E_j is not a conserved Hamiltonian energy. It is a diagnostic quantity that measures how much of the instantaneous response is projected onto each reference modal branch.

7 First-harmonic nonlinear backbone

7.1 Modal cubic tensor

The nonlinear force acts through the same relative coordinate as the delayed linear coupling. With

$$z_h = b^T y(t - h_c) = b^T \Phi_* q(t - h_c) = r^T q(t - h_c), \qquad r = \Phi_*^T b,$$

the physical cubic force becomes

$$\kappa_3 b z_h^3 \quad \longrightarrow \quad \kappa_3 \Phi_*^T b \, [b^T \Phi_* q(t - h_c)]^3 = \kappa_3 r [r^T q(t - h_c)]^3$$

in modal coordinates. Expanding this term gives

$$[\kappa_3 r (r^T q_h)^3]_i = \sum_{j,k,\ell=1}^{2} \Gamma_{ijk\ell} q_j(t - h_c) q_k(t - h_c) q_\ell(t - h_c),$$

where

$$\Gamma_{ijk\ell} = \kappa_3 r_i r_j r_k r_\ell.$$

Therefore the modal cubic tensor is computed directly from κ_3, b, and Φ_*.

7.2 First-harmonic reduction

The backbone approximation starts from the single-harmonic relative-displacement assumption

$$z_h = A_z \cos \Omega(t - h_c).$$

Then

$$z_h^3 = A_z^3 \cos^3 \Omega(t - h_c) = \frac{3}{4} A_z^3 \cos \Omega(t - h_c) + \frac{1}{4} A_z^3 \cos 3\Omega(t - h_c).$$

Since

$$z_h = A_z \cos \Omega(t - h_c),$$

the fundamental component is

$$z_h^3 \approx \frac{3}{4} A_z^2 z_h.$$

The neglected term is the third harmonic

$$\frac{1}{4} A_z^3 \cos 3\Omega(t - h_c).$$

To quantify the amplitude range over which the first-harmonic approximation is used for quantitative interpretation, the retained fundamental-force amplitude and the neglected third-harmonic amplitude are defined as

$$F_1 = \left(k_d \tau_0 + \frac{3}{4} \kappa_3 A_z^2 \right) A_z, \qquad F_3 = \frac{1}{4} \kappa_3 A_z^3.$$

The neglected-harmonic ratio is therefore

$$\varepsilon_3(A_z) = \frac{F_3}{F_1} = \frac{\kappa_3 A_z^2/4}{k_d\tau_0 + 3\kappa_3 A_z^2/4}.$$

A complementary cycle-RMS relative force error is

$$\varepsilon_{\mathrm{RMS}}(A_z) = \frac{F_3}{\sqrt{F_1^2 + F_3^2}}.$$

For $k_d = 0.12$, $\tau_0 = 1.0$, and $\kappa_3 = 0.20$, the neglected-harmonic ratio is approximately 3.37% at $A_z = 0.30$, 9.15% at $A_z = 0.55$, 10.34% at $A_z = 0.60$, and 20.07% at $A_z = 1.10$. The 10% level is reached at approximately $A_z = 0.586$. Accordingly, the quantitative first-harmonic spectral results are restricted to

$$0 \le A_z \le 0.55.$$

The 10% level is an operational truncation-error criterion rather than a nonlinear-stability threshold. Thus the approximation is valid only as a weakly nonlinear first-harmonic backbone. It is not a direct simulation of the complete nonlinear delay differential equation.

7.3 Amplitude-dependent delayed stiffness

Substitution into the physical delayed coupling gives

$$\kappa_3 b z_h^3 \approx \frac{3}{4}\kappa_3 A_z^2 b z_h = \frac{3}{4}\kappa_3 A_z^2 K_c y(t-h_c).$$

Therefore

$$K_{d,\mathrm{eff}}(A_z) = \left(k_d\tau_0 + \frac{3}{4}\kappa_3 A_z^2\right) K_c.$$

The corresponding characteristic equation is

$$\det\left[s^2 M + sC + K_g(\alpha) + K_{d,\mathrm{eff}}(A_z)e^{-sh_c}\right] = 0.$$

For each prescribed A_z, the same root-continuation procedure used in the linear analysis is applied to compute $\alpha_{\mathrm{cov}}(A_z)$, $g_\omega(A_z)$, and the critical-delay curve.

7.4 Numerical values used in main-text Figure 8

The main-text Figure 8 calculation uses

$$\kappa_3 = 0.20, \qquad h_c = 0.30, \qquad A_z \in [0, 0.55].$$

The effective delayed coupling coefficient is

$$k_{\mathrm{eff}}(A_z) = 0.1200 + 0.1500A_z^2.$$

Representative values are:

A_z	$k_{\mathrm{eff}}(A_z)$	α_{cov}	g_ω
0.00	0.1200	1.013105	0.057121
0.30	0.1335	1.016260	0.067930
0.55	0.1654	1.024844	0.092837

7.5 Scope

This first-harmonic backbone calculation explains how cubic delayed coupling deforms the linear delay-sensitive veering structure. It should not be described as a full nonlinear bifurcation analysis, nor as a time-domain stochastic simulation.

8 Frozen stochastic spectral-cloud computation

8.1 Frozen lognormal coupling multiplier

The frozen stochastic spectral analysis uses a stationary snapshot of the coupling modulation:

$$\gamma_\sigma = \exp\left(\sigma\xi - \frac{\sigma^2}{2}\right), \qquad \xi \sim \mathcal{N}(0,1).$$

This construction satisfies

$$\gamma_\sigma > 0$$

and

$$\mathbb{E}\gamma_\sigma = e^{-\sigma^2/2}\mathbb{E}e^{\sigma\xi} = e^{-\sigma^2/2}e^{\sigma^2/2} = 1.$$

Thus the stochastic samples preserve the mean coupling level while excluding negative stiffness realizations.

8.2 Frozen stochastic characteristic equation

The effective delayed stiffness for a frozen sample is

$$K_{d,\mathrm{eff}}(\gamma_\sigma, A_z) = \left(k_d\tau_0\gamma_\sigma + \frac{3}{4}\kappa_3 A_z^2\right) K_c.$$

In the numerical runs, $\tau_0 = 1$, but the full notation is kept for consistency with the dynamic stochastic model. For each realization, the veering-related roots are computed from

$$\det\left[s^2 M + sC + K_g(\alpha) + K_{d,\mathrm{eff}}(\gamma_\sigma, A_z)e^{-sh_c}\right] = 0.$$

The spectral cloud therefore consists of characteristic roots of a random-parameter delay system. It is not produced by adding artificial noise to deterministic roots.

8.3 Random veering center and spectral exchange probability

For a frozen multiplier γ_σ, the random veering center is

$$\alpha_{\mathrm{cov}}(\gamma_\sigma) = \arg\min_{\alpha\in\mathcal{I}_\alpha}[\omega_2(\alpha;\gamma_\sigma, A_z, h_c) - \omega_1(\alpha;\gamma_\sigma, A_z, h_c)].$$

Let

$$\alpha^0_{\mathrm{cov}} = \alpha_{\mathrm{cov}}(1)$$

be the deterministic veering center. The spectral exchange probability used in main-text Figure 9 is

$$P_{\mathrm{exch}}(\alpha,\sigma) = \mathbb{P}\left[\mathrm{sign}(\alpha - \alpha_{\mathrm{cov}}(\gamma_\sigma)) \neq \mathrm{sign}(\alpha - \alpha^0_{\mathrm{cov}})\right].$$

This is a spectral mode-assignment probability: it measures whether the stochastic veering center crosses the deterministic side of the operating detuning. It is a spectral side-crossing probability, not a time-domain switching probability.

8.4 Growth-rate quantiles and tracked positive-growth probability

For each frozen sample, define

$$m^{(n)}_{\mathrm{tr}}(\sigma) = \max_{j=1,2}\mathrm{Re}\, s_j(\alpha_{\mathrm{cloud}}, \gamma^{(n)}_\sigma, A_z, h_c).$$

The plotted growth-rate band contains the empirical 5%, 50%, and 95% quantiles of $m^{(n)}_{\mathrm{tr}}$. The tracked positive-growth probability is

$$\mathbb{P}(m_{\mathrm{tr}} > 0).$$

8.5 Numerical values used in main-text Figure 9

The main-text Figure 9 computation uses

$$A_z = 0.55, \qquad h_c = 0.30, \qquad \alpha_{\text{cloud}} = 1.025.$$

The spectral clouds are displayed for

$$\sigma \in \{0, 0.12, 0.24, 0.36\},$$

with 260 frozen samples per displayed σ. The probability surface uses 2.0×10^4 frozen samples per σ. The maximum characteristic-equation residual is approximately

$$1.31 \times 10^{-14},$$

and no root solve fails.

Representative growth-rate statistics are

σ	$q_{0.05}$	$q_{0.50}$	$q_{0.95}$	$\mathbb{P}(m_{\text{tr}} > 0)$
0.00	0.004104	0.004104	0.004104	1.000
0.12	−0.001784	0.003541	0.010409	0.861
0.24	−0.005519	0.003657	0.017603	0.678
0.36	−0.011436	0.002145	0.020720	0.572

The deterministic nonlinear veering center is

$$\alpha_{\text{cov}}^{(0)} \approx 1.0248.$$

At $\sigma = 0.36$, the empirical 5–95% interval of the random veering center is approximately

$$[1.0104,\ 1.0524].$$

The corresponding tracked-growth 5–95% interval is approximately

$$[-1.14 \times 10^{-2},\ 2.07 \times 10^{-2}],$$

with

$$\mathbb{P}(m_{\text{tr}} > 0) \approx 0.572.$$

8.6 Scope

This section is a frozen-noise spectral analysis. The OU process is used here only through its stationary Gaussian marginal distribution. The dynamically evolving OU-driven time-domain stochastic DDE is analyzed separately in the Monte Carlo simulations.

9 Pathwise energy balance for the stochastic nonlinear delayed system

9.1 Reference mechanical energy

For the nonlinear stochastic delay equation

$$M\ddot{y} + C\dot{y} + K_g(\alpha)y + k_d\tau_0\gamma(t)K_c y(t-h_c) + \kappa_3 b[b^T y(t-h_c)]^3 = 0,$$

define the reference mechanical energy

$$\mathcal{E}(t) = \frac{1}{2}\dot{y}^T(t)M\dot{y}(t) + \frac{1}{2}y^T(t)K_g(\alpha)y(t).$$

Because M and K_g are symmetric and constant,

$$\dot{\mathcal{E}}(t) = \dot{y}^T M\ddot{y} + \dot{y}^T K_g(\alpha)y.$$

Substituting the equation of motion gives

$$\dot{\mathcal{E}}(t) = -\dot{y}^T C\dot{y} - k_d\tau_0\gamma(t)\dot{y}^T K_c y(t-h_c) - \kappa_3\dot{y}^T b[b^T y(t-h_c)]^3.$$

Since $K_c = bb^T$,

$$\dot{y}^T K_c y(t-h_c) = [b^T\dot{y}(t)][b^T y(t-h_c)].$$

Therefore

$$\dot{\mathcal{E}}(t) = -\dot{y}^T C\dot{y} - k_d\tau_0\gamma(t)[b^T\dot{y}(t)][b^T y(t-h_c)] - \kappa_3[b^T\dot{y}(t)][b^T y(t-h_c)]^3.$$

9.2 Interpretation of delayed work rates

The damping term $-\dot{y}^T C\dot{y}$ is non-positive when C is positive semidefinite. The two delayed relative-coordinate work-rate terms are sign-indefinite:

$$-k_d\tau_0\gamma(t)[b^T\dot{y}(t)][b^T y(t-h_c)]$$

and

$$-\kappa_3[b^T\dot{y}(t)][b^T y(t-h_c)]^3.$$

This is the pathwise mechanism by which delay enables energy exchange. The present relative velocity is multiplied by a delayed relative displacement, so the term cannot generally be written as the derivative of an instantaneous potential. The positive OU multiplier changes the intensity of the linear delayed work rate without changing the nominal mean coupling level, and the cubic delayed term amplifies the same relative-coordinate channel at larger delayed amplitudes.

9.3 Modal branch-energy balance

Let

$$y = \Phi_* q, \qquad q_h = q(t-h_c), \qquad r = \Phi_*^T b, \qquad K_{c,q} = \Phi_*^T K_c \Phi_*.$$

The modal equation can be written as

$$\ddot{q} + C_q\dot{q} + K_q(\alpha)q + k_d\tau_0\gamma(t)K_{c,q}q_h + \kappa_3 r[r^T q_h]^3 = 0.$$

The reference modal energy is

$$E_j(t) = \frac{1}{2}\dot{q}_j^2(t) + \frac{1}{2}\omega_{j*}^2 q_j^2(t).$$

Hence

$$\dot{E}_j(t) = \dot{q}_j(t)[\ddot{q}_j(t) + \omega_{j*}^2 q_j(t)].$$

Using the modal equation gives

$$\dot{E}_j = -\dot{q}_j(C_q\dot{q})_j - \dot{q}_j([K_q(\alpha) - \Omega_*^2]q)_j - \dot{q}_j(k_d\tau_0\gamma(t)K_{c,q}q_h)_j - \dot{q}_j(\kappa_3 r[r^T q_h]^3)_j.$$

The four terms represent modal damping, detuning from the reference modal stiffness, delayed stochastic relative-coupling work, and nonlinear delayed relative-coupling work.

9.4 Branch fraction and first-passage statistics

Let

$$E_\Sigma(t) = E_1(t) + E_2(t).$$

Away from the numerical zero-energy regularization, the branch fraction satisfies

$$\dot{R}_2 = \frac{\dot{E}_2 E_\Sigma - E_2(\dot{E}_1 + \dot{E}_2)}{E_\Sigma^2}.$$

Therefore $R_2(t)$ is not merely an empirical postprocessing variable; it is a diagnostic projection of the modal branch-energy balance.

The first-switching time used in the Monte Carlo analysis is a first-passage statistic:

$$T_{\mathrm{sw}} = \inf\{t \geq T_{\mathrm{warm}} : R_2(t) > R_{\mathrm{th}}\}.$$

The survival function is

$$S(t; \alpha, \sigma) = \mathbb{P}(T_{\mathrm{sw}} > t),$$

with non-switched trajectories treated as right-censored at the final simulation time. The residence-based probability

$$P_{\mathrm{TD}}(\alpha, \sigma) = \mathbb{P}(\rho_2 > \rho_{\mathrm{th}})$$

and the survival statistic $S(t; \alpha, \sigma)$ describe different finite-time consequences of the same branch-energy dynamics.

9.5 Scope of the energy mechanism

The pathwise identities above do not constitute a stochastic bifurcation theorem. They explain how delay, cubic coupling, and positive multiplicative noise enter the energy-transfer channels that are subsequently quantified by Monte Carlo residence and first-passage statistics.

10 Time-domain Monte Carlo simulation

10.1 Dynamic stochastic delay model

The time-domain validation uses the nonlinear stochastic delay equation

$$M\ddot{y} + C\dot{y} + K_g(\alpha)y + k_d\tau_0\gamma(t)K_c y(t - h_c) + \kappa_3 b[b^T y(t - h_c)]^3 = 0,$$

where

$$\gamma(t) = \exp(\sigma\eta(t) - \sigma^2/2).$$

The OU process is

$$d\eta = -\theta\eta\, dt + \sqrt{2\theta}\, dW_t,$$

whose stationary distribution is $\mathcal{N}(0, 1)$. Therefore $\gamma(t) > 0$ and has unit stationary mean.

10.2 Exact OU update

The OU process is advanced by the exact one-step update

$$\eta_{n+1} = e^{-\theta\Delta t}\eta_n + \sqrt{1 - e^{-2\theta\Delta t}}\xi_n, \qquad \xi_n \sim \mathcal{N}(0, 1).$$

The initial η_0 is sampled from the stationary distribution. This avoids a transient bias in the stochastic multiplier.

10.3 Delay integration

Writing $v = \dot{y}$, the acceleration is computed from

$$a_n = -M^{-1}\left[Cv_n + K_g(\alpha)y_n + k_d\tau_0\gamma_n K_c y_{n-h} + \kappa_3 b(b^T y_{n-h})^3\right].$$

The numerical update is

$$v_{n+1} = v_n + \Delta t\, a_n, \qquad y_{n+1} = y_n + \Delta t\, v_{n+1}.$$

In the reported simulations,

$$h_c = 0.30, \qquad \Delta t = 0.02,$$

so the delay is exactly 15 time steps. The delayed state is therefore read from a circular history buffer. The initial history is constant on $[-h_c, 0]$.

10.4 Modal branch-energy statistics

For each trajectory, modal coordinates are computed from the same reference projection used in the modal analysis. The branch-energy fraction is

$$R_2(t) = \frac{E_2(t)}{\max(E_1(t) + E_2(t), 10^{-30})}.$$

A branch-2 residence event is defined by

$$R_2(t) > R_{\text{th}}, \qquad R_{\text{th}} = 0.5.$$

After discarding the transient $T_{\text{warm}} = 10$, the branch-2 residence fraction is

$$\rho_2 = \frac{1}{N_{\text{valid}}} \sum_{t_n \geq T_{\text{warm}}} \mathbf{1}_{\{R_2(t_n) > 0.5\}},$$

where only samples above the numerical energy floor are counted. A trajectory is classified as exchange-active if

$$\rho_2 > \rho_{\text{th}}, \qquad \rho_{\text{th}} = 0.10.$$

The time-domain exchange probability is the Monte Carlo estimate

$$P_{\text{TD}}(\alpha, \sigma) = \frac{1}{N_{\text{MC}}} \sum_{m=1}^{N_{\text{MC}}} \mathbf{1}_{\{\rho_2^{(m)} > \rho_{\text{th}}\}}.$$

The first-switching time is

$$T_{\text{sw}} = \inf\{t_n \geq T_{\text{warm}} : R_2(t_n) > 0.5\}.$$

The switching count is the number of changes in the binary threshold state after the transient.

10.5 Numerical settings and representative values

The simulations use

$$T = 100, \quad \Delta t = 0.02, \quad T_{\text{warm}} = 10, \quad \theta = 0.40, \quad A_{\text{init}} = 0.55, \quad N_{\text{MC}} = 256.$$

The parameter grid is

$$\alpha \in \{0.94, 0.952, 0.964, 0.970, 0.976, 0.988, 1.000,$$
$$1.012, 1.024, 1.036, 1.048, 1.060\},$$
$$\sigma \in \{0, 0.072, 0.144, 0.216, 0.288, 0.360\}.$$

At the operating point $\alpha = 0.97$, the aggregate statistics computed from the refined main-text Figure 11 trajectory ensemble are

σ	$\bar{\rho}_2$	P_{TD}	$P(T_{\mathrm{sw}}$ observed)	median T_{sw}
0.000	0.0729	0.0000	1.0000	93.48
0.072	0.0762	0.3008	0.9883	93.48
0.144	0.0901	0.4727	0.9023	90.76
0.216	0.0969	0.4648	0.8086	90.20
0.288	0.1078	0.5000	0.7188	87.46
0.360	0.1382	0.5977	0.7500	84.30

These values show that stochastic coupling modulation broadens the finite-time residence distribution near the chosen operating point. The response is not strictly monotone in σ, so the interpretation should remain a finite-time transition-broadening effect rather than a universal noise-enhancement law.

10.6 Operating-point bootstrap confidence intervals

The following table reports 95% bootstrap confidence intervals at $\alpha = 0.97$ for the refined $N_{\mathrm{MC}} = 256$ ensemble:

σ	P_{TD} [95% CI]	$\bar{\rho}_2$ [95% CI]
0.000	0.0000 [0.0000, 0.0000]	0.0729 [0.0729, 0.0729]
0.072	0.3008 [0.2422, 0.3594]	0.0762 [0.0718, 0.0812]
0.144	0.4727 [0.4141, 0.5352]	0.0901 [0.0824, 0.0978]
0.216	0.4648 [0.4062, 0.5273]	0.0969 [0.0879, 0.1065]
0.288	0.5000 [0.4414, 0.5625]	0.1078 [0.0960, 0.1194]
0.360	0.5977 [0.5331, 0.6544]	0.1382 [0.1240, 0.1540]

The intervals are empirical bootstrap intervals computed from trajectory-level residence statistics. They quantify sampling uncertainty only and do not remove the dependence on the chosen time horizon, initial history, and thresholds.

10.7 Relation to the frozen spectral cloud

Main-text Figures 9 and 11 address complementary questions. Main-text Figure 9 uses frozen random multipliers to quantify spectral uncertainty of the characteristic roots and the veering center. Main-text Figure 11 integrates the nonlinear stochastic delay equation with a time-evolving OU multiplier and measures branch-energy residence in trajectories. Thus, main-text Figure 11 is a trajectory-level counterpart to main-text Figure 9, not a repetition of the same calculation.

10.8 Scope

The time-domain analysis provides Monte Carlo evidence of branch-energy exchange under dynamically evolving multiplicative coupling noise. It does not prove a stochastic bifurcation theorem, and the reported probabilities depend on the simulation horizon, initial condition, threshold values, and sample size.

11 Robustness of stochastic time-domain statistics

This section reports the robustness checks supporting the residence-based Monte Carlo statistics in main-text Figure 11. All checks use the representative operating point $\alpha = 0.97$, $\sigma = 0.36$, unless otherwise stated.

11.1 Time-step convergence

The delay-compatible time steps are chosen so that $h_c/\Delta t$ is an integer. With $h_c = 0.30$, the tested values $\Delta t = 0.03, 0.02, 0.01$ correspond to $10, 15, 30$ delay steps. The results are:

Δt	$h_c/\Delta t$	P_{TD}	$\bar{\rho}_2$	median T_{sw}
0.03	10	0.5703	0.1326	85.41
0.02	15	0.5977	0.1382	84.30
0.01	30	0.6016	0.1323	84.28

The probability and residence fraction stay at the same order across the tested time steps.

11.2 Monte Carlo sample-size sensitivity

At $\Delta t = 0.02$, the sample-size sensitivity is:

N_{MC}	P_{TD}	$\bar{\rho}_2$	median T_{sw}
32	0.7188	0.1769	79.52
64	0.7188	0.1722	81.42
128	0.5469	0.1311	83.98
256	0.5977	0.1382	84.30

The smallest ensembles are more variable, while $N_{\mathrm{MC}} = 128$ and 256 give comparable residence-based estimates.

11.3 Threshold sensitivity

To remain consistent with the threshold-sensitivity audit reported in main-text Figure 12, the finite-time residence probability is evaluated over

$$0.50 \le R_{\mathrm{th}} \le 0.99, \qquad 0.10 \le \rho_{\mathrm{th}} \le 0.95,$$

using 35 uniformly spaced values in each direction. The analysis is repeated for

$$\alpha = 0.8903,\ 0.9303,\ 0.9703,\ 1.0103,\ 1.0503,\ 1.0903,\ 1.1303$$

and

$$\sigma = 0,\ 0.12,\ 0.24,\ 0.36.$$

The sensitivity audit uses $N_{\mathrm{MC}} = 1000$ trajectories per case, while the remaining time-domain settings are consistent with the baseline OU-driven simulations.

Near the reference veering core, at

$$\alpha = 1.0103, \qquad \sigma = 0.24,$$

the residence observable is saturated, with

$$P_{\mathrm{TD}} = 1$$

throughout the complete scanned threshold domain. At the same detuning, changing the stochastic intensity from $\sigma = 0.12$ to 0.36 produces no change in the binary residence probability throughout this domain.

Away from the veering core, the threshold dependence becomes pronounced. For the representative case

$$\alpha = 1.1303, \qquad \sigma = 0.36,$$

the residence probability spans the full interval

$$0 \le P_{\mathrm{TD}} \le 1$$

as the two thresholds are varied. Approximately 29.9% of the scanned threshold plane lies in the intermediate range

$$0.05 < P_{\mathrm{TD}} < 0.95.$$

These results confirm that P_{TD} is a finite-time, threshold-dependent observable whose sensitivity depends strongly on the dynamical location relative to the veering core.

11.4 First-switching survival curves

The first-switching survival function is defined as

$$S(t;\sigma) = \mathbb{P}(T_{\mathrm{sw}} > t).$$

The following aggregate statistics are recomputed from the refined main-text Figure 11. trajectory ensemble with $N_{\mathrm{MC}} = 256$:

σ	P_{TD}	$\bar{\rho}_2$	$P(T_{\mathrm{sw}}$ observed)	median T_{sw}
0.000	0.0000	0.0729	1.0000	93.48
0.072	0.3008	0.0762	0.9883	93.48
0.144	0.4727	0.0901	0.9023	90.76
0.216	0.4648	0.0969	0.8086	90.20
0.288	0.5000	0.1078	0.7188	87.46
0.360	0.5977	0.1382	0.7500	84.30

The corresponding survival curves are shown in Fig. S1, where shaded regions denote 95% bootstrap uncertainty bands. Non-observed first-switching events are treated as right-censored at the simulation horizon.

12 Additional response-level evidence

Main-text Figure 11 provides the primary response-level connection between complex-root veering and time-domain modal participation, while main-text Figures 12 and 13 provide complementary threshold-sensitivity and quantitative energy-contribution audits. This section provides additional supporting figures for the three distinct phenomena considered in the paper: linear veering, nonlinear amplitude dependence, and stochastic branch-energy redistribution. These figures are supplementary diagnostics and do not replace the root-based veering definitions or the rightmost-spectrum validation in the main text. The response figures are generated from the same delayed equations used in the manuscript; the additional time-step audit for the main response figure is provided with the reproducibility files.

13 Claim-scope table

Topic	Safe wording	Avoid
Delay-boundary stability	Analytical imaginary-axis-crossing boundary independently validated numerically in the tested parameter window	Global stability proof of the complete DDE spectrum or of the nonlinear stochastic system
Modal representation	Fixed-reference coordinate representation retaining both physical coordinates	Lossy physical-coordinate truncation or complete modal diagonalization of the infinite-dimensional DDE
Nonlinear backbone	Weakly nonlinear first-harmonic backbone approximation	Complete nonlinear bifurcation analysis

Frozen stochastic spectra	Frozen-noise stochastic spectral analysis	Time-domain stochastic simulation
Time-domain stochastic result	Monte Carlo evidence of trajectory-level branch-energy exchange	Proof of stationary stochastic switching or stochastic bifurcation

14 Supplementary figures

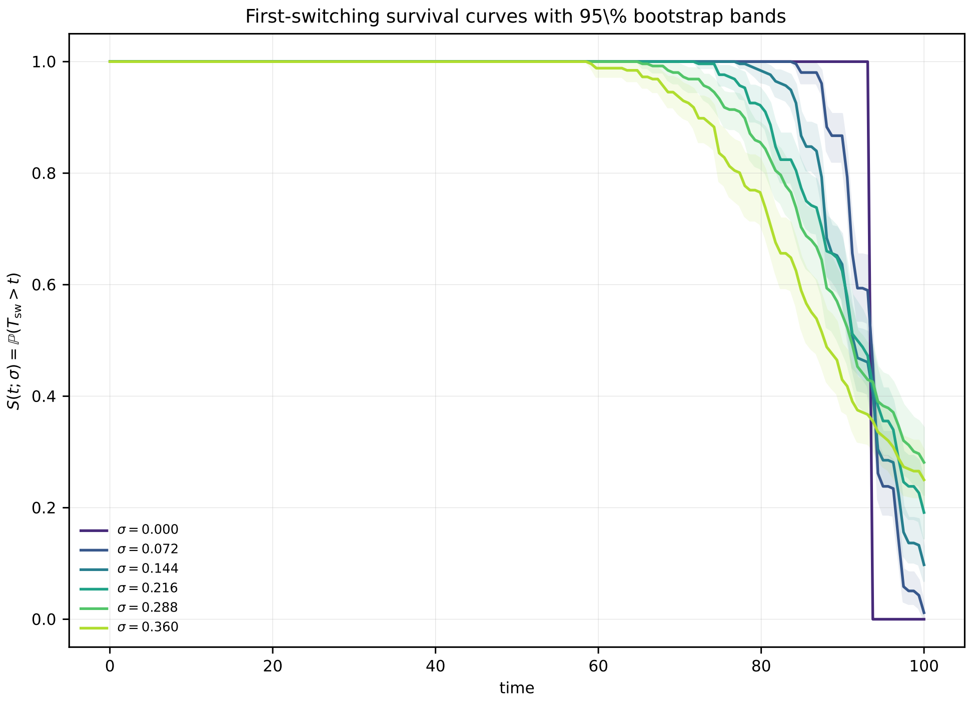

Figure S1: First-switching survival curves at $\alpha = 0.97$ for dynamically evolving OU coupling modulation. Shaded bands denote pointwise 95% bootstrap uncertainty intervals.

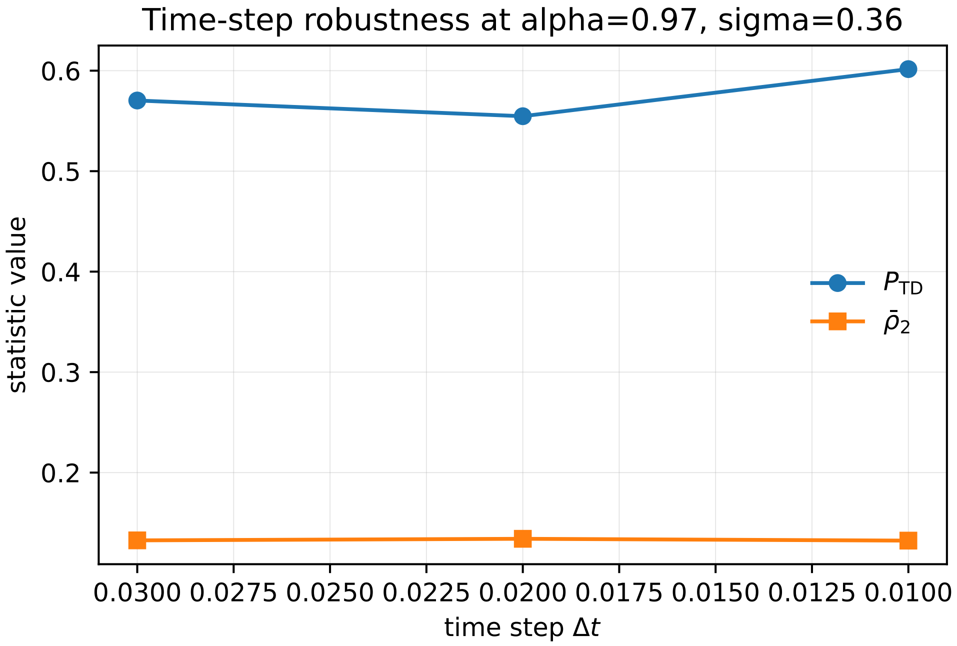

Figure S2: Time-step robustness of the time-domain exchange-active probability and mean branch-2 residence fraction.

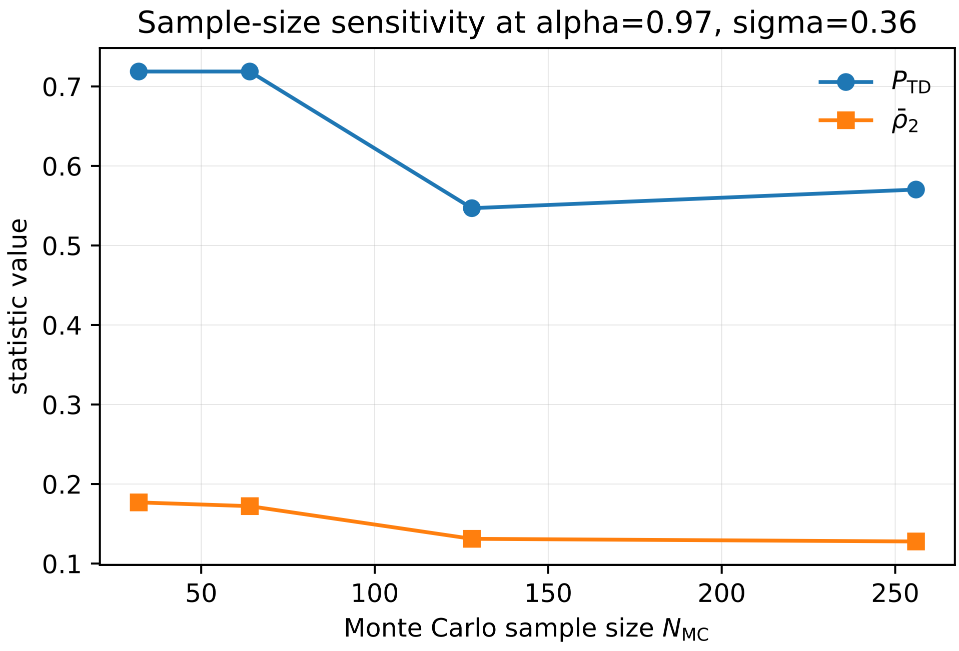

Figure S3: Monte Carlo sample-size sensitivity of the residence-based statistics.

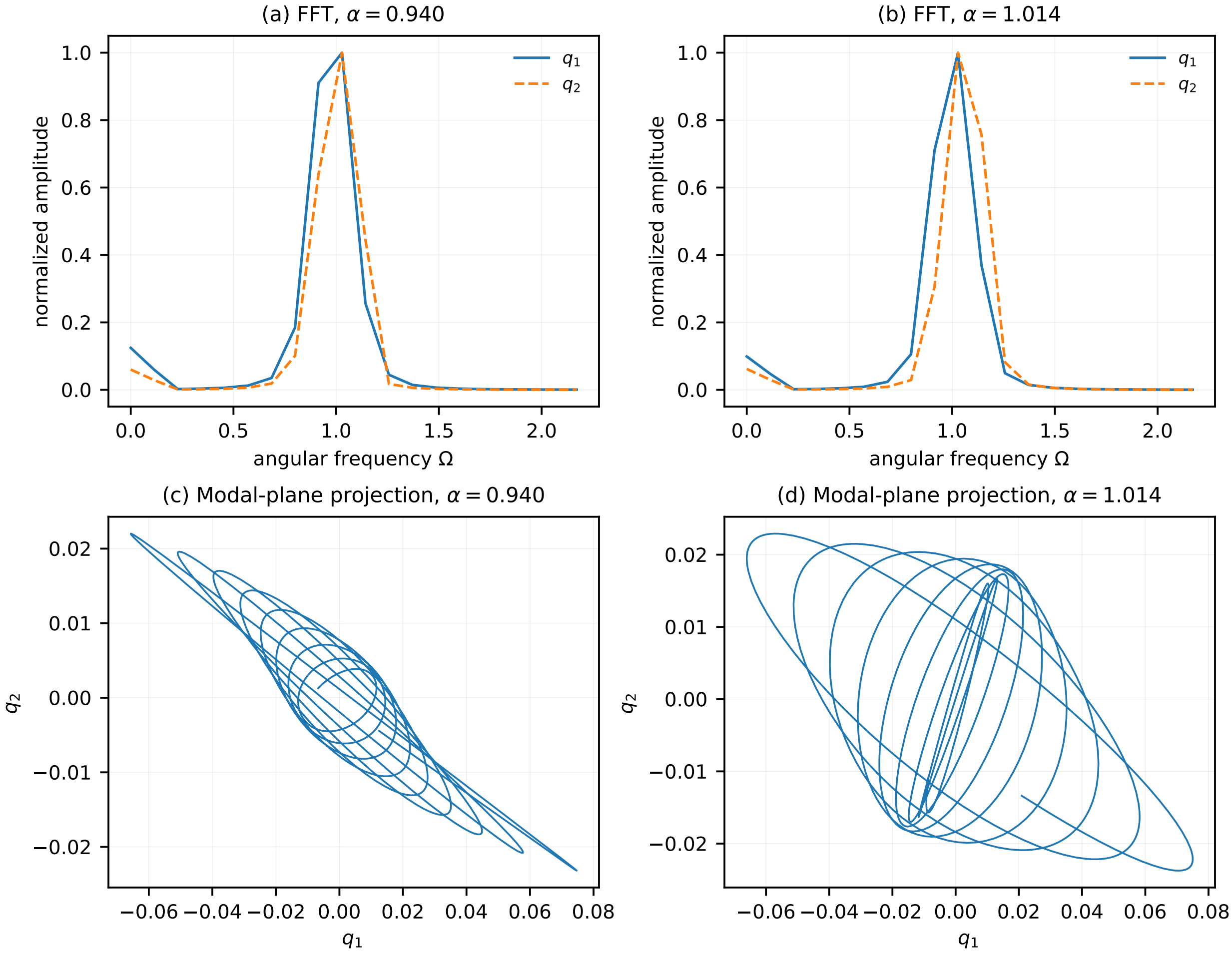

Figure S4: Supplementary linear-response evidence near the veering region. Panels (a)–(b) compare angular-frequency spectra of the modal coordinates away from and near the veering center, while panels (c)–(d) show the corresponding modal-plane projections.

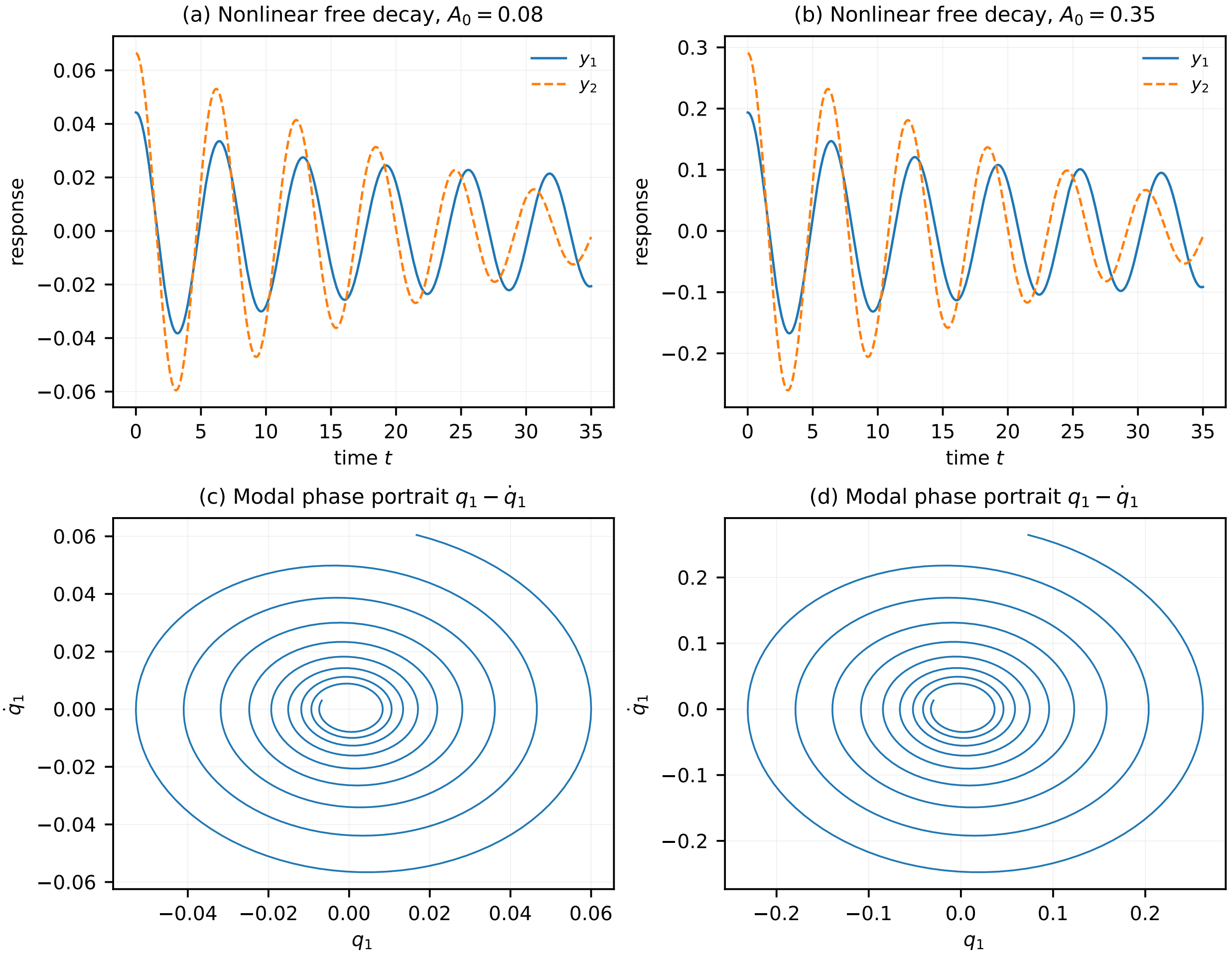

Figure S5: Supplementary nonlinear-response evidence. Low- and high-amplitude deterministic free decays are shown together with modal phase portraits, illustrating the amplitude dependence associated with the delayed cubic relative-coordinate term.

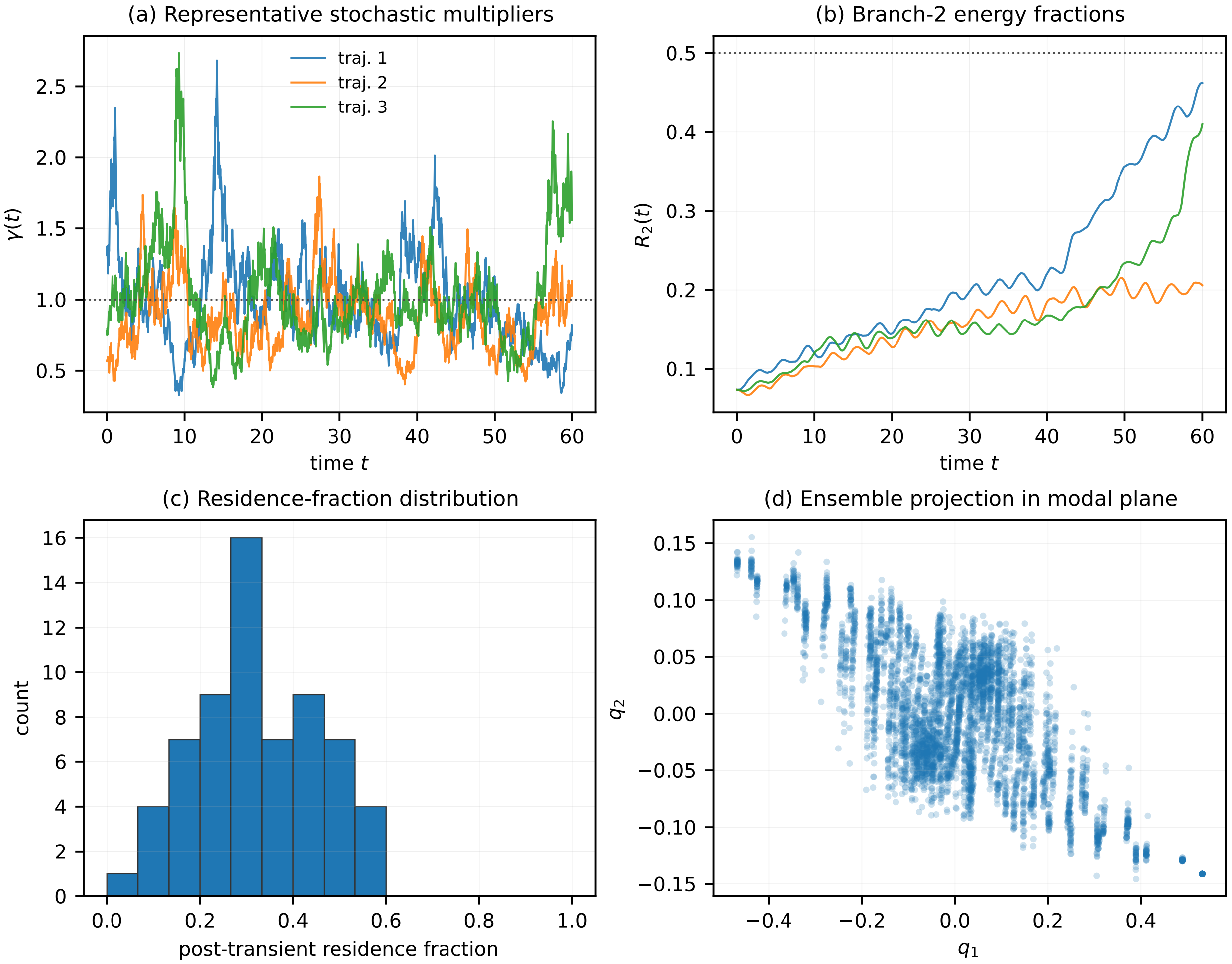

Figure S6: Supplementary stochastic-response evidence. Representative positive stochastic multipliers, branch-energy fractions, residence-fraction statistics, and the modal-plane ensemble projection provide additional pathwise support for the stochastic branch-energy redistribution discussed in the main text.

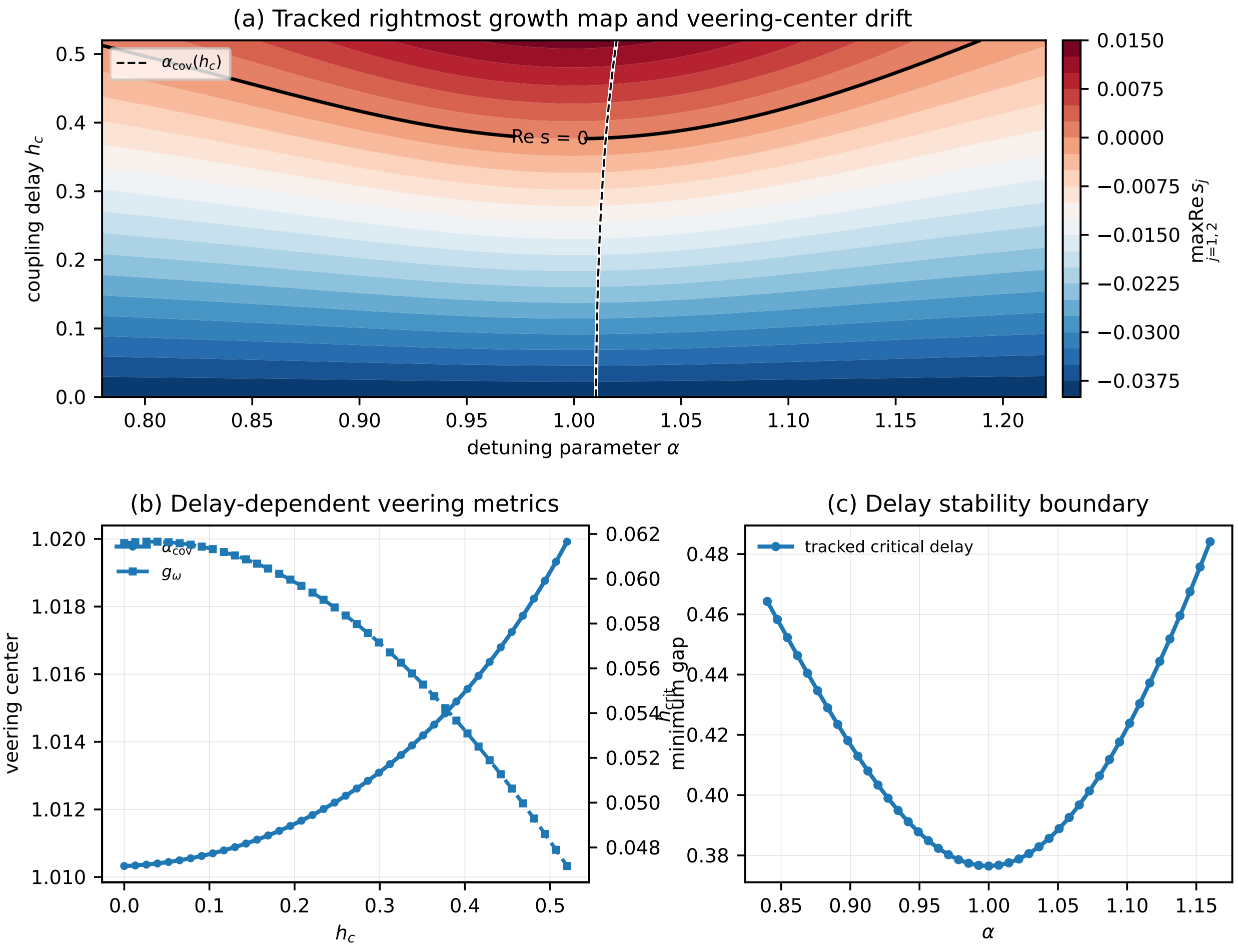

Figure S7: Delay-sensitive veering center, gap, and rightmost boundary.

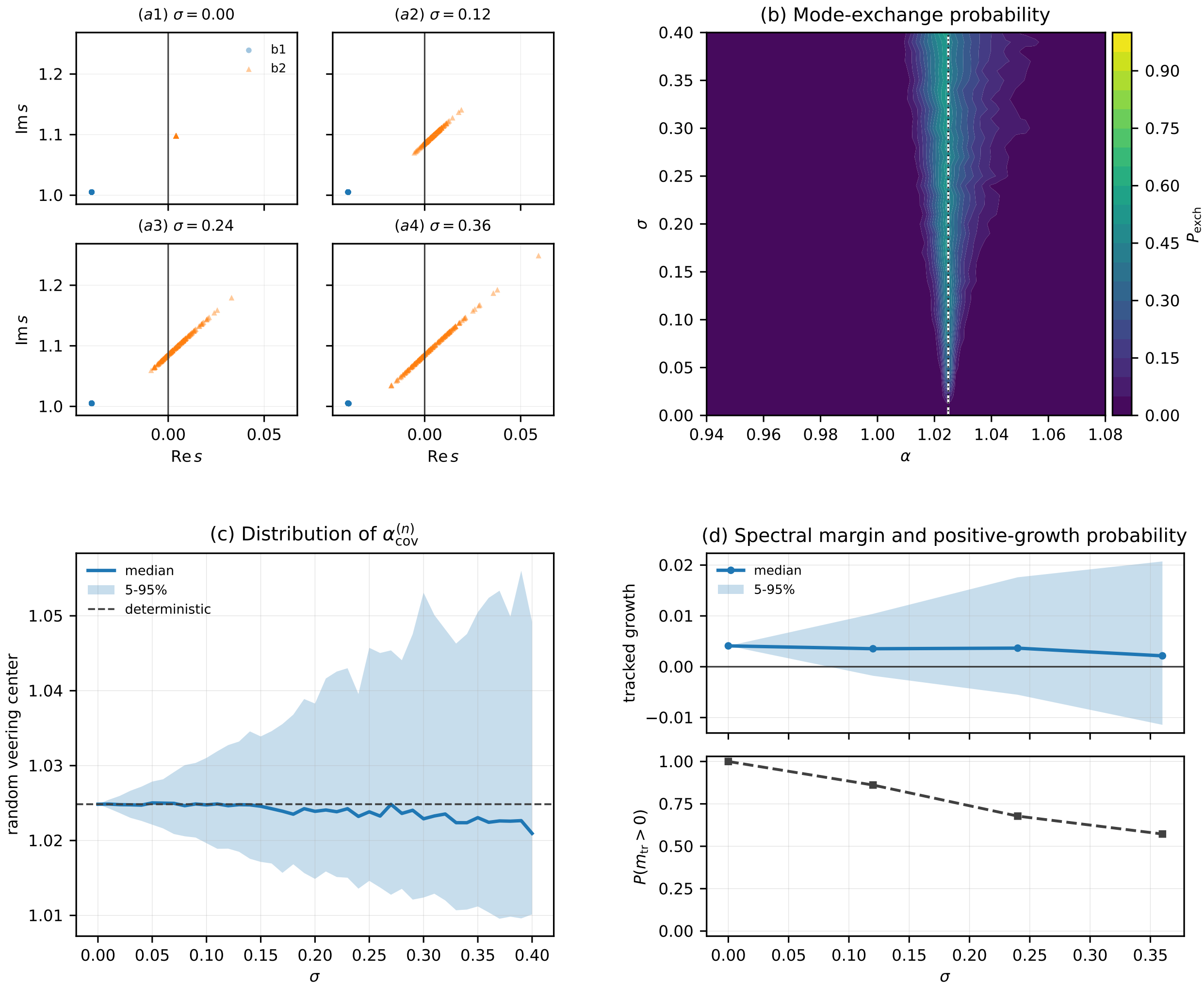

Figure S8: Revised frozen stochastic spectral-cloud visualization.

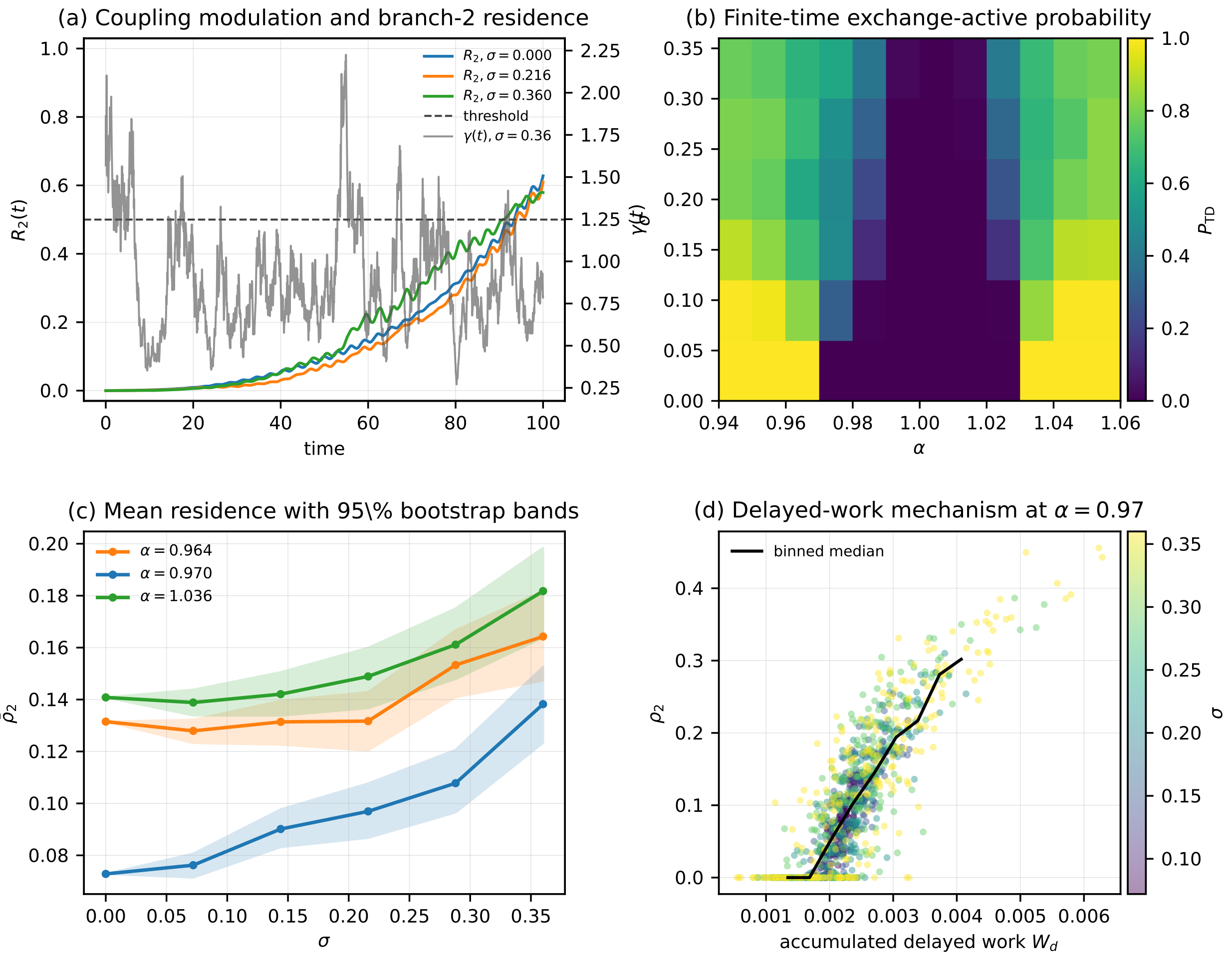

Figure S9: Time-domain stochastic DDE Monte Carlo validation.
